# Childhood Predictors of Dispositional Forgivingness in Adulthood: A Cross-National Analysis with 22 Countries

**DOI:** 10.1007/s11482-025-10451-z

**Published:** 2025-05-07

**Authors:** Richard G. Cowden, Everett L. Worthington Jr., Dorota Weziak-Bialowolska, George Yancey, Charlotte V. O. Witvliet, Koichiro Shiba, R. Noah Padgett, Matt Bradshaw, Byron R. Johnson, Tyler J. VanderWeele

**Affiliations:** 1 Human Flourishing Program, Institute for Quantitative Social Science, Harvard University, Cambridge, MA USA; 2 Department of Epidemiology, Harvard T.H. Chan School of Public Health, Boston, MA USA; 3 Department of Psychology, Virginia Commonwealth University, Richmond, VA USA; 4 Department of Quantitative Methods & Information Technology, Kozminski University, Warsaw, Poland; 5 Department of Sociology, Baylor University, Waco, TX USA; 6 Psychology Department, Hope College, Holland, MI USA; 7 Department of Epidemiology, Boston University School of Public Health, Boston, MA USA; 8 Institute for Studies of Religion, Baylor University, Waco, TX USA

**Keywords:** Child development, Forgiveness, Life course, Population wellbeing, Global flourishing study

## Abstract

A robust body of empirical evidence suggests that forgiveness of others is positively related to individual wellbeing. However, less empirical work has been done to identify the factors that may help children not only forgive better during childhood but also develop into adults who practice forgiveness more consistently. To support a population health agenda aimed at the promotion of forgiveness, further research is needed to identify potential determinants of forgiveness. In this preregistered study, we used the first wave of nationally representative data from 22 countries included in the Global Flourishing Study (*N* = 202,898) to explore associations of 13 individual characteristics and retrospectively assessed childhood factors with dispositional forgivingness in adulthood. We estimated country-level modified Poisson models in which forgivingness was regressed on all candidate predictors, and then aggregated results for the 11 predictors that were common across countries using a random effects meta-analysis. Risk ratios from the meta-analyses showed that a combination of individual characteristics (e.g., older birth cohort, female gender), early life conditions or experiences (e.g., more frequent religious service attendance, better health, more secure family financial status), and social circumstances or influences when growing up (e.g., higher quality maternal and paternal relationships) were associated with a higher likelihood of forgivingness in adulthood. Associations were somewhat heterogeneous across the countries. Our findings suggest that childhood may be important in shaping forgivingness in adulthood and provide some potential foci for population-level interventions.

**Supplementary Information:**

The online version contains supplementary material available at 10.1007/s11482-025-10451-z.

Forgiveness of others is a virtue that has deep roots in collective human history, and is a central part of many religious/spiritual traditions and cultures around the world (McCullough et al., 2005). Although definitions of forgiveness vary, social scientists mostly agree that forgiveness is a process characterized by a prosocial change in one’s thoughts, feelings, motivations, or behaviors toward a transgressor for an interpersonal transgression that has caused one hurt or harm (Toussaint et al., 2023; Witvliet, 2020). The term *dispositional forgivingness* reflects the tendency to respond to interpersonal hurts or offenses with forgiveness across time and situations (Cowden et al., 2023).

Evidence from lab experiments, longitudinal studies, and randomized controlled trials suggests that forgiveness is related to improved health and wellbeing across mental, physical, social, and religious/spiritual dimensions of human life (Cook et al., 2022; Cowden, 2024; Toussaint et al., 2023). Because people are often hurt or wronged by others and the benefits of forgiveness to health and wellbeing may be quite substantial, forgiveness has been identified as a public health issue with important implications for population wellbeing (VanderWeele, 2018). A growing number of studies have applied a public health lens to the promotion of forgiveness. Recent evidence indicates that forgiveness can be effectively promoted in community settings through self-directed workbooks (Ho et al., 2024) and highly engaging awareness-raising campaigns providing information and skills training (Bechara et al., 2024).

Although public health interventions are central to promoting forgiveness at the population level, more basic epidemiologic research aimed at identifying determinants of forgiveness can complement these efforts by revealing potential foci for intervention. Several interventions have sought to help children learn to practice forgiveness (for a meta-analysis, see Rapp et al., 2022). However, the interventions to date have merely sought to test their efficacy without targeting specific childhood predictors of forgiveness. Little is known about what factors might help children not only forgive better when they are children but also grow into adults who might practice forgiveness more consistently. To our knowledge, no prior study has applied a life-course perspective to identify childhood predictors of adult forgiveness. Such evidence would (1) provide insight into at-risk subpopulations that might benefit from early-life interventions, (2) establish possible targets for intervention with non-at-risk children, and (3) contribute to identifying factors that could be targeted by parents, teachers, or people who work with children to promote childhood growth in practicing the virtue of forgiveness. Furthermore, for adults who want to forgive, such research evidence could illuminate which childhood features most potently predict forgivingness, thereby enhancing self-awareness about possible areas to address. In the present study, we use the first wave of nationally representative data from 22 countries in the Global Flourishing Study (GFS) to explore several individual characteristics and recalled childhood factors as potential predictors of forgivingness in adulthood.

## Epidemiology of Forgivingness in Adulthood: A Socioecological Life-Course Lens

Empirical research has converged on a view that forgiveness is an intrapersonal experience (it occurs within the victim’s ‘own skin’) that unfolds within the broader socioecological system in which the person is embedded (Sandage et al., 2020; Worthington & Cowden, 2017). Applying a socioecological life-course lens, a person’s disposition to forgive others is influenced by earlier formative events and experiences that have unfolded within a multilayered system of interconnected immediate and extended environments (Tomlinson et al., 2021). The current study focuses on the socioecological context of childhood, given that childhood is the earliest period of postnatal development and exposure to risk (e.g., parental death or divorce) and protective (e.g., healthy parent-child attachment) factors during childhood can have important implications for wellbeing later in life (Auersperg et al., 2019; Han et al., 2023).

Although there are different ways to conceptualize the socioecology of child development, socioecological systems theory (Bronfenbrenner, 2005) partitions the socioecological system of human development into four theoretical layers (micro, meso, exo, and macrosystem) and a temporal layer (the chronosystem). This lens is useful for exploring the notion that forgiveness can be shaped by a broad range of childhood factors nested within contextual layers of proximal or distal influence. The microsystem includes people with whom the child interacts (e.g., parents, teachers, peers, youth sport coaches) in the immediate environments of daily life (e.g., at home, school). Individuals who are part of the child’s microsystem might hurt or offend the child, producing unforgiveness, but they might also model forgiveness (or grudge-holding) to the child (Ermer et al., 2022). The mesosystem involves interactions between different elements in the microsystem (e.g., positive parent-teacher interactions might support aspects of the child’s intellectual and social development that could contribute to nurturing a disposition to forgive). The exosystem refers to contexts that do not contain the child but may influence the child indirectly by impinging on the microsystem (e.g., a parent’s conflict with a colleague might ‘poison’ the home environment). The macrosystem refers to cultural influences on the life and development of the child (e.g., political polarization can affect parents, parent-teacher interactions, or workplace dynamics). Cultural differences in the way people in the various interlocking systems deal with conflict could affect ongoing hurts and produce unforgiveness in the child. Finally, the chronosystem considers how a child’s socioecology intersects with time (e.g., children who begin middle school after forgiveness education has been integrated into the curriculum might have different experiences with forgiveness than children who completed middle school before this change was made to the curriculum). While the various layers of a child’s socioecology are interconnected and can influence each other in intricate ways, researchers and interventionists usually prioritize highly proximal layers (e.g., microsystem) because factors within those layers generally tend to have more of a direct influence on the individual (Eriksson et al., 2018; Tomlinson et al., 2021). The present study takes a similar approach by attending to candidate childhood predictors within the microsystem, but we engage with broader layers of the socioecological system to contextualize our findings.

At present, existing empirical evidence concerning potential predictors of forgiveness is mostly restricted to individual-level factors situated within a particular phase of human development (largely adulthood). For instance, a number of cross-sectional studies with adult samples have shown that female gender, older age, and greater religious/spiritual engagement (e.g., religious service attendance) are related to higher scores on measures of forgiveness (for meta-analyses, see Davis et al., 2013; Fehr et al., 2010; Miller et al., 2008). However, some studies have examined associations of childhood events, experiences, or influences with forgiveness in adulthood. One strand of evidence comes from research on adverse childhood experiences (e.g., abuse, neglect, bullying). Some randomized controlled trials with adult survivors of child maltreatment have shown that unforgiveness can understandably continue into adulthood and that survivors who participate in forgiveness interventions can experience sustained forgiveness levels posttreatment (Freedman & Enright, 2020). In observational studies that have explored the link between exposure to adverse childhood events and forgiveness in adulthood, findings have been somewhat mixed. For example, one cross-sectional study with a sample of *N* = 360 Malaysian adult males receiving inpatient treatment for substance use reported a very large negative association between experiencing maltreatment in childhood and forgivingness (Mey et al., 2022), whereas another found little evidence of an association between a history of childhood bullying and forgivingness in a sample of *N* = 301 adult Americans receiving outpatient medical treatment (Sansone et al., 2014).

On occasion, empirical research has also addressed positive childhood influences on forgiveness. In a cross-sectional study with *N* = 113 undergraduate students in the United States, Romig and Veenstra (1998) reported evidence suggesting that those who successfully resolved early stages of psychosocial development (e.g., trust versus mistrust) tended to score higher on forgiveness. Other studies have broadened beyond specific events or experiences to explore the connection between social influences during childhood and forgiveness in adulthood (Akl & Mullet, 2010; Aloia & Pederson, 2021). To illustrate, in a cross-sectional study of *N* = 182 French adults, Akl and Mullet (2010) examined the associations of several retrospectively recalled dimensions of family forgivingness while growing up with forgivingness in adulthood. For several dimensions of family forgivingness (e.g., tendency for the family to practice unconditional forgiveness), those who had more positive memories about forgivingness in their family reported more favorable scores on selected dimensions of their own forgivingness (e.g., less likely to harbor resentment).

While the available evidence provides some foundational support for the idea that early life experiences can have important implications for forgiveness in adulthood, there are important gaps in the empirical literature. First, most prior studies have addressed one or a small set of childhood factors (typically those at the level of the individual). To build a more comprehensive picture of childhood factors that predict forgiveness in adulthood, there is a need for additional research addressing factors within other layers of the broader socioecological system (e.g., microsystem). Research along these lines resonates with a life-course epidemiological approach to forgiveness (Kuh et al., 2003), which would improve our understanding of the factors that ought to be prioritized in childhood intervention work centered on forgiveness and help guide decisions about how to allocate resources to promote healthy child development. Second, existing evidence is largely confined to single-shot studies that have focused on population segments (e.g., university students, medical patients). Notwithstanding the value of research on specific subpopulations, sample variation can add to the complexities of comparing results across studies, and it remains unclear whether the findings from previous studies on childhood predictors of forgiveness in adulthood can be generalized more broadly. Without evidence at the population level, it can be difficult for public health authorities and policymakers to make well-informed decisions about how to support population wellbeing through childhood interventions. Third, the emerging wave of “global wellbeing science” (Lomas, 2022, p. 264) points to the need for evidence that is more inclusive and globally representative of the human population (Wong & Cowden, 2022). Research with geographically and culturally diverse populations can shed light on what is similar and distinct about people who are situated within different socioecological systems, including childhood factors that predict forgiveness in adulthood.

## The Present Study

In this preregistered study, we aim to address some of these gaps in knowledge by employing a life course epidemiological approach to explore candidate predictors of forgivingness later in life. More specifically, we use nationally representative data from 22 countries that were included in the first wave of the GFS to examine 13 candidate predictors of forgivingness in adulthood, ranging from individual characteristics (e.g., year of birth, gender) to early life conditions or experiences (e.g., child abuse, religious service attendance) and social circumstances or influences (e.g., family structure, relationships with parents) within the microsystem of a child’s socioecology. Among the predictors included, we expected that some factors would show evidence of associations with forgivingness in adulthood across all countries. Given the diversity of sociocultural contexts that characterize the 22 countries, we anticipated that there would be some cross-national variation in the pattern of associations between the candidate predictors and forgivingness in adulthood.

## Methods

The methodological details described below have been adapted from VanderWeele et al. (in press). Further methodological information is available elsewhere (Crabtree et al., 2021; Johnson et al., 2024; Lomas et al., in press; Padgett et al., in press; Padgett et al., 2025; Padgett et al., 2024; Ritter et al., 2024). The research questions, variables, and analytic plan for the current study were preregistered prior to accessing data, with only slight subsequent modification to the initial preregistered regression analyses due to multicollinearity (https://osf.io/4qbaj/).

### Study Sample

Wave 1 of the GFS included nationally representative samples from 22 geographically and culturally diverse countries: Argentina, Australia, Brazil, Egypt, Germany, Hong Kong (Special Administrative Region of China), India, Indonesia, Israel, Japan, Kenya, Mexico, Nigeria, the Philippines, Poland, South Africa, Spain, Sweden, Tanzania, Türkiye, the United Kingdom, and the United States (*N* = 202,898). The countries were selected to (1) maximize coverage of the world’s population, (2) ensure geographic, cultural, and religious diversity, and (3) prioritize feasibility in line with existing data collection infrastructure. Data collection was conducted by Gallup Inc. Data for Wave 1 was collected primarily during 2023, although some countries began data collection in 2022 and exact dates of data collection varied to some extent by country (Ritter et al., 2024). Plans are in place to collect four additional waves of annual panel data on the participants from 2024 to 2027. The GFS survey centers on salient aspects of wellbeing, such as happiness, health, meaning, character, relationships, and financial stability (VanderWeele, 2017), along with other sociodemographic, social, economic, political, religious/spiritual, personality, childhood, and community variables. Gallup translated the GFS survey into multiple languages following the TRAPD (translation, review, adjudication, pretesting, and documentation) model for cross-cultural survey research (Padgett et al., 2025). Extensive details about the translation, cognitive interviewing, and piloting testing phases of the GFS can be found elsewhere (Cowden et al., 2025; Crabtree et al., 2021; Lomas et al., in press; Padgett et al., 2025).

### Sampling Design

The precise sampling design that was used to ensure samples were nationally representative varied by country (Padgett et al., 2025; Ritter et al., 2024). In most countries, local field partners were guided in implementing a probability-based face-to-face or telephone methodology to recruit panel members. Recruitment involved an intake survey that mainly gathered basic sociodemographics and information for recontacting participants. Shortly following recruitment, participants received invitations to participate in the annual survey via phone or online. Three major sampling frames were used for recruitment in the GFS, namely a probability-based sample, a nonprobability-based sample, or a combination of the two. Post-stratification and nonresponse adjustments were carried out within each country separately using census data or a reliable secondary source. Additional information about the sampling design used in Wave 1 of the GFS is available in Padgett et al. (2025) and Ritter et al. (2024)

## Measures

### Predictor Variables

Given our principal interest in the potential effects of more proximal factors within a child’s socioecological system, all retrospectively recalled childhood factors available in Wave 1 of the GFS were initially considered. Due to multicollinearity concerns, four of the retrospectively recalled childhood factors were excluded: (1) feeling loved by mother, (2) feeling loved by father, (3) religious service attendance of mother, and (4) religious service attendance of father. Individual characteristics (e.g., birth year, gender) were restricted to those we could reasonably assume were not instrumental variables (i.e., mediators). Further details about the decision-making involved in selecting the 13 candidate predictors included in the current study are available in Padgett et al. (in press).

Quality of relationship with mother when growing up was assessed with the question: “Please think about your relationship with your mother when you were growing up. In general, would you say that relationship was very good, somewhat good, somewhat bad, or very bad?” Responses were dichotomized to very bad/somewhat bad versus very good/somewhat good. An analogous variable was used for the quality of relationship with father when growing up. Both maternal and paternal relationship quality were dichotomized (i.e., very bad/somewhat bad versus very good/somewhat good) in the regression analyses to help address multicollinearity issues. “Does not apply” was treated as a dichotomous control variable for respondents who did not have a mother or father due to death or absence. Family structure around age 12 was assessed with: “Were your parents married to each other when you were around 12 years old?” Responses included married, divorced, never married, and one or both had died. Subjective financial status of family around age 12 was measured with: “Which one of these phrases comes closest to your own feelings about your family’s household income when you were growing up, such as when you were around 12 years old?” Responses were lived comfortably, got by, found it difficult, and found it very difficult. Childhood abuse when growing up was assessed with yes/no responses to: “Were you ever physically or sexually abused when you were growing up?” Similarly, participants provided a yes/no response to whether they felt like an outsider in their family when growing up: “When you were growing up, did you feel like an outsider in your family?” Self-rated health when growing up was assessed by: “In general, how was your health when you were growing up? Was it excellent, very good, good, fair, or poor?” Immigration status was assessed with: “Were you born in this country, or not?” Frequency of religious service attendance around age 12 was assessed with: “How often did you attend religious services or worship at a temple, mosque, shrine, church, or other religious building when you were around 12 years old?” Responses were at least once/week, 1–3 times/month, less than once/month, or never. Gender was assessed as male, female, or other. Year of birth (age) was classified as 1998–2005 (18–24 years), 1993–1998 (25–29 years), 1983–1993 (30–39 years), 1973–1983 (40–49 years), 1963–1973 (50–59 years), 1953–1963 (60–69 years), 1943–1953 (70–79 years), and 1943 or earlier (80 years or older).

Religious affiliation at age 12 was also assessed in all countries, but there were considerable cross-country differences in the response categories endorsed by participants because some religious affiliations are only applicable in certain countries and not others. Religious affiliation response category options included Christianity, Islam, Hinduism, Buddhism, Judaism, Sikhism, Baha’i, Jainism, Shinto, Taoism, Confucianism, Primal/animist/folk religion, Spiritism, Umbanda, Candomblé, and other African-derived religions, Chinese folk/traditional religion, some other religion, or no religion/atheist/agnostic. When more than 5% of a within-country sample endorsed the no religion/atheist/agnostic category, this was used as the reference category in the country-specific analyses; otherwise, the most prominent religious group was used. Additionally, all religious affiliation categories endorsed by less than 3% of a within-country sample were collapsed into a single religious affiliation category. Racial/ethnic identity was assessed in most (18/22) countries, and response categories varied across countries. Country-specific analyses used a binary racial/ethnic identity variable based on whether an individual was in the most prominent group in the sample versus a minority group. Additional details about measurement of the predictors can be found in the GFS Codebook (https://osf.io/cg76b*).*

### Outcome Variable

The measure of dispositional forgivingness in the GFS survey comes from the Forgiveness-Short Form measure that is part of the Brief Multidimensional Measure of Religiousness/Spirituality (Fetzer Institute, 1999). The Forgiveness-Short Form contains three items, one for each of three types of forgiveness: forgiveness of others, self-forgiveness, and divine forgiveness. The original forgiveness of others item (i.e., “I have forgiven those who hurt me”; response options: ‘Never,’ ‘Seldom,’ ‘Often,’ ‘Always or almost always’) has been used in previous research (e.g., Hirsch et al., 2011), and other studies have employed variations of this item as well (e.g., Long et al., 2020). To strengthen the cross-cultural equivalence of the original item for use in the GFS survey, slight modifications were made to the phrasing and response categories based on the results of cognitive interviews that were performed during the GFS survey development process (Cowden et al., 2025; Crabtree et al., 2021; Lomas et al., in press; Padgett et al., 2025). The version of the item used in the GFS survey is: “How often have you forgiven those who have hurt you?” (response options: ‘Never,’ ‘Rarely,’ ‘Often,’ ‘Always’). Following prior work using Wave 1 GFS data (e.g., Cowden et al., in press), in our primary analysis we dichotomized responses to this variable into (0) never/rarely and (1) often/always. We also perform a sensitivity analysis with an alternative dichotomization of (0) never/rarely/often and (1) always.

### Statistical Analysis

Unless otherwise specified, all analyses were preregistered and performed using R 4.2.2 (R Core Team, 2024). All code to reproduce analyses is openly available in an online repository (Padgett et al., 2024).

Descriptive statistics, weighted to be nationally representative within country, were estimated for each category of the candidate predictors. A weighted modified Poisson regression model (with complex survey-adjusted standard errors) was fit within each of the 22 countries separately, with the outcome of forgivingness regressed on all 13 candidate predictors simultaneously. When available, religious affiliation and racial/ethnic identity were also included as control variables. We conducted a global test of association between each candidate predictor and the outcome in each country. For each candidate predictor, we calculated *E*-values to evaluate the sensitivity of results to unmeasured confounding. An *E*-value is the minimum strength of the association an unmeasured confounder must have with both the predictor and the outcome, above and beyond all measured covariates, for an unmeasured confounder to explain away an association (VanderWeele & Ding, 2017). The lowest *E*-value is 1, with higher values indicating that stronger unmeasured confounding would be needed to explain away the observed association.

The *metafor* package (Viechtbauer, 2010) was used to perform random effects meta-analyses of the regression coefficients (Borenstein et al., 2010; Hunter & Schmidt, 2000) along with 95% confidence intervals (CIs), estimated proportions of effects across countries with effect sizes smaller than 0.90 and larger than 1.10, and *I*^2^ for evidence concerning variation within a given category across countries (Mathur & VanderWeele, 2020). Further details about the meta-analytic methodology that was used can be found in Padgett et al. (in press). Coefficients for religious affiliation and racial/ethnic identity were not included in the meta-analyses because these variables were not measured consistently across all countries. A pooled *p*-value (Wilson, 2019) was used across countries to report whether there is evidence of an association in at least one country. Bonferroni corrected *p*-value thresholds are provided based on the number of candidate predictors that were included in the meta-analyses: *p* = 0.05/11 = 0.0045 (VanderWeele & Mathur, 2019). *E*-values were estimated for the regression coefficients included in the meta-analyses.

As a supplementary analysis, we conducted population weighted meta-analyses of the same regression coefficients that were included in the random effects meta-analyses. We also performed a post-hoc sensitivity analysis in which we repeated the random effects analyses using an alternative dichotomization of forgivingness (i.e., never/rarely/often versus always).

### Missing Data

Missing data on all variables were imputed using multivariate imputation by chained equations, with five imputed datasets produced (Sterne et al., 2009; van Buuren, 2018). To account for variation in the assessment of certain variables across countries (e.g., religious affiliation, racial/ethnic identity), the imputation process was conducted separately in each country. This within-country imputation approach ensured that the imputation models accurately reflected country-specific contexts and assessment methods. Sampling weights were included in the imputation models to account for specific variable missingness that may have been related to the probability of inclusion in the study.

### Accounting for Complex Sampling Design

Wave 1 of the GFS used different sampling schemes across countries based on availability of existing panels and recruitment needs (Ritter et al., 2024). All analyses accounted for the complex survey design components by including weights, primary sampling units, and strata. Additional methodological details, including the approach used to account for the complex sampling design, can be found elsewhere (Padgett et al., in press; Padgett et al., 2025).

## Results

### Descriptive Statistics

The distribution of descriptive statistics in the sample is presented in Table 1. Countries with the largest samples included the United States (19%), Japan (10%), and Sweden (7.4%), while the smallest samples were from Türkiye (0.7%), South Africa (1.3%), and Hong Kong (1.5%). The gender distribution was roughly balanced between females (51%) and males (49%). The entire adult lifespan (18 to ≥ 80 years old) is represented in the sample, with the cohort that had the largest percentage of participants (20%) born in 1983–1993 (current age of 30–39 years). Most participants were born in the country where data was collected (94%). A majority of participants reported that their parents were married when they were about 12 years of age (75%), they had a very good relationship with their mother (63%) and father (53%) when growing up, their family either got by financially or lived comfortably when they were around age 12 (76%), they had very good or excellent health while growing up (64%), and they attended religious services at least once a month when they were around age 12 (57%). A minority of participants indicated that they experienced abuse (14%) or felt like an outsider in their family when growing up (14%).

**Table 1:** Nationally representative descriptive statistics of the observed sample (*N* = 202,898)

Characteristic	*n* (%)
**Relationship with mother when growing up**	
Very good	127,836 (63%)
Somewhat good	52,439 (26%)
Somewhat bad	11,060 (5.5%)
Very bad	4,642 (2.3%)
Does not apply	5,965 (2.9%)
Missing	956 (0.5%)
**Relationship with father when growing up**	
Very good	107,742 (53%)
Somewhat good	55,714 (27%)
Somewhat bad	15,807 (7.8%)
Very bad	8,278 (4.1%)
Does not apply	13,985 (6.9%)
Missing	1,372 (0.7%)
**Family structure around age 12**	
Parents were married	152,001 (75%)
Parents were divorced	17,726 (8.7%)
Parents were never married	15,534 (7.7%)
One or both parents had died	7,794 (3.8%)
Missing	9,843 (4.9%)
**Subjective financial status of family around age 12**	
Lived comfortably	70,861 (35%)
Got by	82,905 (41%)
Found it difficult	35,852 (18%)
Found it very difficult	12,606 (6.2%)
Missing	674 (0.3%)
**Experienced abuse when growing up**	
Yes	29,139 (14%)
No	167,279 (82%)
Missing	6,479 (3.2%)
**Felt like an outsider in the family when growing up**	
Yes	28,732 (14%)
No	170,577 (84%)
Missing	3,589 (1.8%)
**Self-rated health when growing up**	
Excellent	67,121 (33%)
Very good	63,086 (31%)
Good	47,378 (23%)
Fair	19,877 (9.8%)
Poor	4,906 (2.4%)
Missing	530 (0.3%)
**Immigration status**	
Born in this country	190,998 (94%)
Born in another country	9,791 (4.8%)
Missing	2,110 (1.0%)
**Frequency of religious service attendance around age 12**	
≥ 1/week	83,237 (41%)
1–3/month	33,308 (16%)
< 1/month	36,928 (18%)
Never	47,445 (23%)
Missing	1,980 (1.0%)
**Year of birth**	
1998–2005 (current age: 18–24 years)	27,007 (13%)
1993–1998 (current age: 25–29 years)	20,700 (10%)
1983–1993 (current age: 30–39 years)	40,256 (20%)
1973–1983 (current age: 40–49 years)	34,464 (17%)
1963–1973 (current age: 50–59 years)	31,793 (16%)
1953–1963 (current age: 60–69 years)	27,763 (14%)
1943–1953 (current age: 70–79 years)	16,776 (8.3%)
1943 or earlier (current age: 80 + years)	4,119 (2.0%)
Missing	20 (< 0.1%)
**Gender**	
Male	98,411 (49%)
Female	103,488 (51%)
Other	602 (0.3%)
Missing	397 (0.2%)
**Country**	
Argentina	6,724 (3.3%)
Australia	3,844 (1.9%)
Brazil	13,204 (6.5%)
Egypt	4,729 (2.3%)
Germany	9,506 (4.7%)
Hong Kong (S.A.R. of China)	3,012 (1.5%)
India	12,765 (6.3%)
Indonesia	6,992 (3.4%)
Israel	3,669 (1.8%)
Japan	20,543 (10%)
Kenya	11,389 (5.6%)
Mexico	5,776 (2.8%)
Nigeria	6,827 (3.4%)
Philippines	5,292 (2.6%)
Poland	10,389 (5.1%)
South Africa	2,651 (1.3%)
Spain	6,290 (3.1%)
Sweden	15,068 (7.4%)
Tanzania	9,075 (4.5%)
Türkiye	1,473 (0.7%)
United Kingdom	5,368 (2.6%)
United States	38,312 (19%)

*Note*. S.A.R.: Special Administrative Region.

Nationally representative descriptive statistics for each country are reported in Tables S1a-S22a, with some variability observed across the countries. For example, the percentage of individuals who attended religious services at least once/week around age 12 was considerably higher in Indonesia and Kenya (Tables S8a and S11a) than in Japan and Sweden (Tables S10a and S18a). Rates of reported childhood abuse were also much lower in Indonesia and Poland (Tables S8a and S15a) compared to Australia and the United States (Tables S2a and S22a). As these examples indicate, early life experiences, conditions, and influences are at least somewhat idiosyncratic across the countries.

### Cross-Country Predictors of Forgivingness in Adulthood

Results of the random effects meta-analyses are reported in Table 2, with country-specific results presented in Tables S1b-S22b. Pooling across the countries, 95% CIs for the effect estimates provided evidence of an association with forgivingness in adulthood for seven of the candidate predictors (though effect sizes were generally very small). There was a higher likelihood of forgivingness in adulthood among individuals who reported a very good/somewhat good relationship with their mother (RR = 1.06, 95% CI: 1.03, 1.08) and father (RR = 1.03, 95% CI: 1.01, 1.05) when growing up relative to those who reported a very bad/somewhat bad maternal/paternal relationship. Relative to individuals who characterized their family as one that got by financially when they were around age 12, there was a higher likelihood of forgivingness in adulthood among those who reported that their family lived comfortably around age 12 (RR = 1.01, 95% CI: 1.00, 1.02). There was a higher likelihood of forgivingness among individuals who attended religious services at least once/week (RR = 1.11, 95% CI: 1.08, 1.14), 1–3 times/month (RR = 1.10, 95% CI: 1.07, 1.13), and less than once/month (RR = 1.06, 95% CI: 1.04, 1.08) around age 12 compared to those who never attended. Relative to individuals who reported having good health while growing up, there was a higher likelihood of forgivingness in adulthood among those who reported very good (RR = 1.02, 95% CI: 1.01, 1.03) or excellent health (RR = 1.03, 95% CI: 1.01, 1.05). The likelihood of forgivingness in adulthood was higher among females relative to males (RR = 1.02, 95% CI: 1.00, 1.04). There was somewhat of a monotonic association between year of birth and forgivingness in adulthood, with the oldest cohort (born in 1943 or earlier) evidencing the strongest positive association with forgivingness (RR = 1.12, 95% CI: 1.04, 1.21) relative to the youngest cohort (born in 1998 to 2005). There was little evidence suggesting that family structure around age 12, experiencing abuse when growing up, feeling like an outsider in one’s family when growing up, or immigration status were associated with forgivingness in adulthood when pooled across the countries, though statistical uncertainty in some of the estimates is notable.

**Table 2:** Random effects meta-analyses for associations of candidate predictors with forgivingness in adulthood

Variable	RR (95% CI)	Estimated proportion of effects by threshold		*I* ^2^	Global *p*-value
		< 0.90	> 1.10		
**Relationship with mother when growing up**					< 0.001
Ref: Very bad/somewhat bad	-	-	-	-	
Very good/somewhat good	1.06 (1.03, 1.08)	0.00	0.05	32.5	
**Relationship with father when growing up**					< 0.001
Ref: Very bad/somewhat bad	-	-	-	-	
Very good/somewhat good	1.03 (1.01, 1.05)	0.00	0.05	44.5	
**Family structure around age 12**					< 0.001
Ref: Parents were married	-	-	-	-	
Parents were divorced	1.00 (0.98, 1.02)	0.00	0.00	38.2	
Parents were never married	0.99 (0.97, 1.00)	0.00	0.00	12.0	
One or both parents had died	1.01 (0.98, 1.04)	0.00	0.09	67.3	
**Subjective financial status of family around age 12**					< 0.001
Ref: Got by	-	-	-	-	
Lived comfortably	1.01 (1.00, 1.02)	0.00	0.00	22.5	
Found it difficult	1.00 (0.99, 1.01)	0.00	0.00	< 0.1ǂ	
Found it very difficult	1.00 (0.98, 1.01)	0.00	0.00	< 0.1ǂ	
**Experienced abuse when growing up** ^a^					< 0.001
Ref: No	-	-	-	-	
Yes	0.99 (0.98, 1.01)	0.00	0.00	54.4	
**Felt like an outsider in the family when growing up**					< 0.001
Ref: No	-	-	-	-	
Yes	0.99 (0.97, 1.00)	0.00	0.00	54.8	
**Self-rated health when growing up**					< 0.001
Ref: Good	-	-	-	-	
Excellent	1.03 (1.01, 1.05)	0.00	0.05	68.2	
Very good	1.02 (1.01, 1.03)	0.00	0.00	43.7	
Fair	0.99 (0.96, 1.01)	0.00	0.00	60.6	
Poor	1.01 (0.97, 1.05)	0.00	0.09	63.9	
**Immigration status**					< 0.001
Ref: Born in this country	-	-	-	-	
Born in another country	1.02 (0.99, 1.04)	0.00	0.00	43.8	
**Frequency of religious service attendance around age 12**					< 0.001
Ref: Never	-	-	-	-	
≥ 1/week	1.11 (1.08, 1.14)	0.00	0.68	68.5	
1–3/month	1.10 (1.07, 1.13)	0.00	0.41	72.8	
< 1/month	1.06 (1.04, 1.08)	0.00	0.09	43.4	
**Year of birth**					< 0.001
Ref: 1998–2005 (current age: 18–24 years)	-	-	-	-	
1993–1998 (current age: 25–29 years)	1.03 (1.01, 1.04)	0.00	0.00	43.7	
1983–1993 (current age: 30–39 years)	1.03 (1.01, 1.05)	0.00	0.00	55.8	
1973–1983 (current age: 40–49 years)	1.04 (1.02, 1.08)	0.00	0.18	80.6	
1963–1973 (current age: 50–59 years)	1.05 (1.02, 1.08)	0.00	0.18	77.1	
1953–1963 (current age: 60–69 years)	1.07 (1.02, 1.12)	0.00	0.32	87.4	
1943–1953 (current age: 70–79 years)	1.08 (1.03, 1.14)	0.05	0.36	84.0	
1943 or earlier (current age: 80 + years)	1.12 (1.04, 1.21)	0.00	0.45	83.5	
**Gender**					< 0.001
Ref: Male	-	-	-	-	
Female	1.02 (1.00, 1.04)	0.00	0.05	86.4	
Other	0.27 (0.05, 1.53)	0.56	0.28	100.0	

*Note*. ^a^Meta-analysis for experienced abuse when growing up only includes results for 21 countries because this question was not used in Israel. All *p*-values passed the Bonferroni-corrected threshold of *p* < 0.0045 (*p* = 0.05/11).

CI: confidence interval, RR: risk ratio. Estimated proportion of effects by threshold: The estimated proportion of effects across the implied distribution of effects sizes within the countries that are either less than a risk ratio of 0.90 or above a risk ratio of 1.10. *I*^2^: An estimate of the percent of variability in effect sizes that cannot be attributed to sampling variability.

Global *p*-value: A test of the null hypothesis that there is no association between the predictor and forgivingness in adulthood in all the countries.

Additional heterogeneity metrics of the effects are provided in Figures S1-S27, along with the associated Q-statistics for the heterogeneity in the effects on the log(RR) scale. ǂEstimate of heterogeneity is likely unstable (see Figures S7 and S8 for forest plots that provide more detail on heterogeneity).

Results of the *E*-value sensitivity analysis for the random effects meta-analyses can be found in Table 3. *E*-values corresponding with the effect estimates for the various candidate predictors were almost all below 1.50 (ranging from 1.01 to 1.49), with the exception being the ‘other’ gender category that had an *E*-value of 6.82 but the estimate had a very wide confidence interval (the *E*-value for the confidence interval limit was 1.00). Besides the ‘other’ gender category, *E*-values for the confidence interval limits ranged from 1.00 to 1.38. For associations that excluded the null, *E*-values for the effect estimate (1.12 to 1.49) and confidence interval limit (1.05 to 1.38) suggested that some of these associations might be moderately robust to potential unmeasured confounding. For example, to explain away the observed association between attending religious services at least once/week around age 12 and forgivingness in adulthood, an unmeasured confounder that was associated with both variables by risk ratios of 1.46-fold each (above and beyond the other predictors adjusted for in the model) could do so, but weaker joint confounder associations could not. For the limit of the confidence interval, unmeasured confounder risk ratio associations of 1.38 for weekly or more frequent religious service attendance as well for forgivingness in adulthood could shift the confidence interval to include the null, but weaker joint confounder associations could not.

**Table 3:** Sensitivity of meta-analyzed candidate predictors to unmeasured confounding

Variable	*E*-values^a^	
	Effect estimate^b^	95% CI^c^
**Relationship with mother when growing up**		
Ref: Very bad/somewhat bad	-	-
Very good/somewhat good	1.30	1.22
**Relationship with father when growing up**		
Ref: Very bad/somewhat bad	-	-
Very good/somewhat good	1.22	1.13
**Family structure around age 12**		
Ref: Parents were married	-	-
Parents were divorced	1.01	1.00
Parents were never married	1.12	1.00
One or both parents had died	1.10	1.00
**Subjective financial status of family around age 12**		
Ref: Got by	-	-
Lived comfortably	1.12	1.05
Found it difficult	1.04	1.00
Found it very difficult	1.07	1.00
**Experienced abuse when growing up**		
Ref: No	-	-
Yes	1.08	1.00
**Felt like an outsider in the family when growing up**		
Ref: No	-	-
Yes	1.14	1.00
**Self-rated health when growing up**		
Ref: Good	-	-
Excellent	1.22	1.14
Very good	1.16	1.09
Fair	1.13	1.00
Poor	1.10	1.00
**Immigration status**		
Ref: Born in this country	-	-
Born in another country	1.16	1.00
**Frequency of religious service attendance around age 12**		
Ref: Never	-	-
≥ 1/week	1.46	1.38
1–3/month	1.43	1.33
< 1/month	1.31	1.24
**Year of birth**		
Ref: 1998–2005 (current age: 18–24 years)	-	-
1993–1998 (current age: 25–29 years)	1.19	1.09
1983–1993 (current age: 30–39 years)	1.20	1.11
1973–1983 (current age: 40–49 years)	1.26	1.14
1963–1973 (current age: 50–59 years)	1.27	1.15
1953–1963 (current age: 60–69 years)	1.35	1.18
1943–1953 (current age: 70–79 years)	1.38	1.20
1943 or earlier (current age: 80 + years)	1.49	1.26
**Gender**		
Ref: Male	-	-
Female	1.17	1.06
Other	6.82	1.00

*Note*. ^a^The formula for calculating *E*-values can be found in VanderWeele and Ding (2017). ^b^The *E*-value for the effect estimate is the minimum strength of association (on the risk ratio scale) that an unmeasured confounder would need to have with both the predictor and the outcome to entirely explain away the observed association between them, conditional on the measured covariates. ^c^The *E*-value for the limit of the 95% confidence interval closest to the null denotes the minimum strength of association (on the risk ratio scale) that an unmeasured confounder would need to have with both the predictor and the outcome to shift the confidence interval to include the null value, conditional on the measured covariates.

CI: confidence interval.

When we repeated the meta-analyses using a population weighted approach in which each country’s results were weighted by population size in 2023 (see Table S23), the results were comparable to those observed for the random effects meta-analyses and there were generally negligible differences in the magnitude of effect sizes (e.g., a risk ratio of 1.05 versus 1.03 for quality of paternal relationship when growing up). The pattern of results was also similar when the random effects analyses were replicated after dichotomizing the outcome of forgivingness into (0) never/rarely/often and (1) always (see Table S24), although effect sizes for some categories of certain predictors (e.g., health when growing up, subjective financial status of family around age 12, frequency of religious service attendance around age 12) evidenced somewhat stronger associations with higher forgivingness in adulthood when this alternative coding of forgivingness was used (e.g., a risk ratio of 1.18 versus 1.03 for excellent health when growing up).

### Similarities and Differences Across Countries

In addition to the meta-analytic pooled estimates of the associations, Table 2 also provides evidence of cross-national variation in the associations between the candidate predictors and forgivingness in adulthood. The metrics reported on (proportion of effects by thresholds, *I*^2^, global *p*-values) each provide evidence of heterogeneity, and a more detailed description of the heterogeneity of these effects can be found in the forest plots displaying country-specific effect estimates and 95% CIs for each category of the candidate predictors (see Figures S1-S27). There was some evidence of cross-national heterogeneity in associations for all candidate predictors, although heterogeneity was greater for some predictors compared to others. For example, associations for feeling like an outsider in one’s family when growing up with forgivingness in adulthood were generally more heterogeneous across countries (*I*² = 54.8) than associations for quality of relationship with mother when growing up (*I*² = 32.5). Cross-national heterogeneity in associations also varied across categories of each predictor themselves. To illustrate, for frequency of religious service attendance at age 12 (reference category of never), the association between attending religious services at least once/week and forgivingness in adulthood was somewhat more heterogeneous across countries compared to the association for attending less than once/month (proportions below RR = 0.90 and above RR = 1.10 were 0% and 68% versus 0% and 9%, respectively). These findings suggest that the potential effects of the candidate predictors on forgivingness in adulthood may vary to some extent across sociocultural contexts.

All global *p*-values in Table 2 were below the Bonferroni-corrected significance threshold, indicating that each candidate predictor was associated with forgivingness in adulthood in at least one of the countries. The country-specific results for candidate predictors of forgivingness are presented in Tables S1b-S22b; country-specific effect estimates for each candidate predictor category are also displayed visually using forest plots, with countries ordered on the y-axis by magnitude of association (see Figures S1-S27). We did not find evidence of a completely universal pattern of associations between the candidate predictors and forgivingness in adulthood. However, many of the predictors for which there was evidence of association with higher forgivingness in the random effects meta-analyses also showed evidence of association in this direction in a range of countries. For example, country-specific results for attending religious services at least once/week (see Figure S16) and 1–3 times/month around age 12 (see Figure S17) showed a positive association with forgivingness (95% CIs did not include the null) in most countries (14/22 and 12/22, respectively), which were among the strongest positive predictors of forgivingness when estimates were pooled across countries in the random effects meta-analyses. However, even in cases where the pattern of associations was more consistent across countries, effect estimates were often somewhat varied. For instance, risk ratios for the association between attending religious services at least once/week and forgivingness in adulthood ranged from RR = 0.99 to 1.31. There were only a few countries where some predictors showed evidence of a negative association (95% CIs did not include the null) with forgivingness in adulthood; the predictor for which this occurred in the largest number of countries (3/22) was feeling like an outsider in the family while growing up (see Figure S10).

While most predictors were generally consistent in their direction of associations with forgivingness in adulthood across countries, the pattern of associations for some predictors was more mixed. For example, 95% CIs for effect estimates indicated that feeling like an outsider in one’s family when growing up was associated with a lower likelihood of forgivingness in 3/22 countries and a higher likelihood of forgivingness in 3/22 countries (see Figure S10). In addition to feeling like an outsider in one’s family when growing up, there were other country-specific instances in which we found evidence of an association between a candidate predictor and forgivingness in adulthood that was not observed in the pooled random effects meta-analyses. To illustrate, while the 95% CI for the association between parents were divorced (versus married) around age 12 and forgivingness in adulthood included the null when estimates were pooled across countries, there was evidence (95% CIs did not include the null) of a negative association with forgivingness in 2/22 countries and a positive association in 2/22 countries (see Figure S3). There were also some cases where evidence of a positive association found in the pooled random effects meta-analyses was in the negative direction for certain countries. One example of this is the birth cohort of being born from 1973 to 1983 (age 40–49 years), as there were 2/22 countries where the 95% CI for its negative association with forgivingness in adulthood did not include the null (see Figure S21).

Country-specific models also estimated the associations of religious affiliation at age 12 and racial/ethnic identity (when available), which were not meta-analyzed because these variables were assessed differently across countries (see Tables S1b-S22b). There were 8/22 countries where 95% CIs for effect estimates provided evidence of associations between one or more categories of childhood religious affiliation and forgivingness in adulthood. Of these eight countries, in the six where no religion/atheist/agnostic at age 12 served as the reference category, there were five countries (Germany, Japan, Spain, Sweden, the United Kingdom) where the likelihood of forgivingness in adulthood was higher among individuals who self-identified at age 12 with a dominant religion in that country (e.g., Buddhism, Christianity, Islam), two countries (Germany, Tanzania) where the likelihood of forgivingness was higher among those in the ‘other’ religious affiliation category, and one country (Sweden) where the likelihood of forgivingness was lower among those in the ‘other’ religious affiliation category. For the other two countries, there was a higher likelihood of forgivingness among those affiliated with Islam at age 12 relative to those in the Hindu religious affiliation category in India and a lower likelihood of forgivingness among those in the Islam or ‘other’ religious affiliation categories at age 12 relative to those in the Judaism category in Israel. There were 4/18 countries in which 95% CIs for the effect estimates supported associations between racial/ethnic identity and forgivingness in adulthood. In one of those countries (India), racial/ethnic minority status was associated with a higher likelihood of forgivingness; it was associated with a lower likelihood of forgivingness in adulthood in the other three countries (Kenya, Mexico, the United States).

## Discussion

The present study leveraged data from 22 geographically and culturally diverse countries to explore associations of 13 individual characteristics and recalled childhood factors with forgivingness in adulthood. Pooled effect estimates across countries suggested that a combination of individual characteristics (older birth cohort, female gender), early life conditions or experiences (more frequent religious service attendance, better health, more secure family financial status), and social circumstances or influences (better maternal and paternal relationship quality) within a child’s system of socioecology may be associated with a higher likelihood of forgivingness in adulthood. Notably, the magnitude of effect estimates varied across predictors, with some cross-national variation in the pattern of associations observed. By considering a range of potential childhood determinants in a multinational dataset that represents approximately half of the global human population, this study expands existing knowledge of how early-life events, experiences, and conditions in a wide range of cultures and contexts are associated with the disposition to forgive others in adulthood.

### Cross-Country Predictors of Forgivingness in Adulthood

Of the 11 candidate predictors that were considered in the random effects meta-analyses, there was some evidence of an association with forgivingness in adulthood for seven of them (most effect sizes were very small). Consistent with a socioecological life course approach to human wellbeing (Tomlinson et al., 2021), these findings suggest that a variety of early-life factors within a child’s socioecological system may have the potential to shape the disposition to forgive others in adulthood. Although the candidate childhood predictors that were considered in the present study were limited to the microsystem, it is the first to apply a socioecological life course lens to study dispositional forgivingness using nationally representative data from many diverse countries.

Among the candidate predictors for which we found evidence of an association with forgivingness in adulthood in the random effects meta-analyses, attending religious services at least once/week emerged as one of the strongest (though still somewhat small in magnitude) predictors of higher forgivingness. This coheres with prior research documenting a positive association between adulthood religious/spiritual engagement (e.g., religious service attendance) and forgiveness, and advances knowledge about the importance of religious/spiritual participation during the early years of life for the disposition of forgivingness in adulthood. Regular service attendance around age 12 likely reflects consistent support from one or more adults to get to and from the service location, as well as regular encounters with the community that gathers for worship. Because forgiveness is an important concept in many religious/spiritual traditions (McCullough et al., 2005), regular attendance of religious services when growing up may provide more frequent opportunities for children to grow in their content and process knowledge of forgiveness through scriptures, teachings, worship practices, and encountering role models of forgiveness that can influence the development of a young person’s disposition to respond with forgiveness when hurt or wronged by others (Mullet et al., 2003; Wuthnow, 2000). These practices during childhood could sustain forgivingness in adulthood, even for those who have changed from the religious tradition they affiliated with during childhood (Van Tongeren et al., 2021).

There was some slightly weaker evidence indicating that individuals who had better quality maternal and paternal relationships when growing up had a higher likelihood of forgivingness in adulthood. These results indicate that adults’ memories of the quality of their childhood relationship with their mother or father are positively associated with their disposition to forgive others in adulthood, whereas family structure around age 12 showed little evidence of association with adult forgivingness. The findings for parental relationship quality are consistent with results from young adults whose trusting and communicative parental attachment correlated positively with forgivingness, whereas parental alienation was negatively correlated with forgivingness (Lawler-Row et al., 2011). Further, a meta-analysis demonstrated that anxious and avoidant adult attachment was negatively related to dispositional forgivingness (Hirst et al., 2019). The current multinational study advances knowledge by showing that retrospective positive parental attachment when growing up predicted adult levels of forgivingness.

Of the other childhood factors that were examined, there was marginal evidence of a positive association between excellent health when growing up and forgivingness in adulthood. While this finding resonates with a sizable body of empirical literature that has documented associations between forgiveness and better physical health (Toussaint et al., 2020), virtually all prior studies have considered health as an outcome. The present research suggests that the quality of a person’s health during the early years of life may itself have a longer-term influence on the development of a disposition to forgive others, and that future generations’ capacity for forgiveness might be strengthened through initiatives aimed at promoting their health during childhood.

Although the positive association between reporting that one’s family lived comfortably around age 12 and forgivingness in adulthood was more marginal, it may be that secure material circumstances during childhood could support the cultivation of a forgiving disposition. Children who grow up in families characterized by greater financial security may have more opportunities to develop the virtue of forgiveness through reduced strain to meet basic needs and access to enriching opportunities (e.g., attend character-oriented schools or extracurricular activities where forgiveness is taught, modeled, and valued). However, given the scarcity of prior research concerning the link between the subjective financial status of one’s family during childhood and subsequent forgiveness in adulthood, more in-depth analyses are required to explore this theorizing further.

We also found that higher forgivingness was predicted by being born in an older birth cohort (effect sizes were generally very small though increased in size with increasing age) and (to a lesser extent) female gender. These findings align with earlier research that has shown older individuals (Fehr et al., 2010) and females (Miller et al., 2008) tend to report higher forgiveness. Whereas few prior studies have used data that is sufficient for generalizing to the broader population, the current analysis of nationally representative data from multiple countries around the world takes a step toward addressing this gap in knowledge.

Applying a systems lens (Höltge et al., 2023), the pattern of findings in this study suggests there may be utility in implementing multipronged early-life interventions and policy initiatives targeting different aspects of a child’s socioecology (e.g., relationships with parents, participation in religious community, material security) that support the development of a disposition to forgive, which would likely affect numerous other wellbeing outcomes as well (VanderWeele et al., in press). Equipping families with education and access to resources that support parent-child secure attachment, child health, the financial capacity to meet needs, and participate in the services of their religious community may be especially vital for wellbeing, with implications for cultivating forgivingness at the population level if the reach of interventions targeting those factors is extensive (Komura et al., 2023). Such efforts might be especially impactful when paired with strategies aimed at preventing adverse childhood experiences (e.g., abuse) that could undermine the effectiveness of early-life initiatives designed to foster a forgiving disposition (Shonkoff et al., 2012). By targeting a combination of salient childhood factors, children may be exposed to a wider range of opportunities to learn about healthy relationships and forgiveness, observe others modeling forgiveness, learn how their faith connects to accountable repentance and forgiveness, and integrate the practice of forgiveness into their repertoire of tools for responsively and responsibly navigating interpersonal relationships throughout life. However, when resource limitations are unable to support a multipronged intervention approach, our findings could be used to guide decisions about which factors to prioritize, taking into account the observed results (their magnitude and uncertainty), how each factor might impact health and wellbeing over the life course, and the resources needed to appropriately intervene on each factor within the relevant cultural context.

### Similarities and Differences Across Countries

When country-specific results were inspected, we observed some cross-national similarities and differences in the pattern of associations. Attending religious services at least once/week evidenced the most consistent association with forgivingness in adulthood across the countries, with 95% CIs for effect estimates supporting a positive association in 14/22 countries. While effect sizes varied to some extent across countries, this fairly consistent association across countries underscores the potential longer-term benefits that regular religious service attendance during the early years of life can have on the development of a forgiving disposition among people from a range of countries that vary in socioecological system dynamics. Other findings suggest that the influence of some candidate predictors on forgivingness in adulthood may be more limited to specific countries. For example, 95% CIs for the association between feeling like an outsider in one’s family when growing up and forgivingness in adulthood supported a positive association in 3/22 countries (Hong Kong, India, Türkiye) and a negative association in 3/22 countries (Kenya, the Philippines, the United States). Although there may be several reasons for this pattern of findings (e.g., differences in population demographics), insights from socioecological systems theory would suggest that this early life relational experience could have different impacts on shaping forgivingness in adulthood depending on the broader socioecological context in which it occurs (Bronfenbrenner, 2005). Further work is needed to better understand whether (and to what extent) the influence of proximal factors within the child’s socioecological system (e.g., feeling like an outsider in the family) on cultivating a forgiving disposition in adulthood might vary cross-nationally based on factors situated within more distal layers of the socioecological system (e.g., the macrosystem).

As a further indication of the unique socioecological context of each country, the pattern of associations observed for the full set of candidate predictors tended to vary across countries. To illustrate, in 4/22 countries (India, Japan, Sweden, the United States), 95% CIs for effect estimates indicated that more than half of the candidate predictors were associated with forgivingness in adulthood, whereas in the other 18/22 countries (e.g., Argentina, the Philippines, the United Kingdom) evidence of association was found for less than half of the candidate predictors. Such cross-country variation highlights the need for awareness of, and the potential to develop policies and interventions that are responsive to, the country-specific factors during childhood which may promote (or undermine) the development of a forgiving disposition. Population health practitioners and policymakers should be sensitive to country-specific socioecological system dynamics that could impact the effectiveness of early-life initiatives aimed at stimulating childhood growth in the practice of forgiveness.

### Limitations and Future Research Directions

Although this study has many strengths, including the large sample size and nationally representative samples, there are also some limitations. First, candidate childhood predictors were assessed retrospectively in the same wave that the outcome of forgivingness was measured. Therefore, our findings may be influenced by recall and common method bias. For example, it is possible that some of the recalled childhood experiences might have been perceived more negatively by those who reported lower forgivingness. Recall bias can also vary based on participant characteristics (e.g., age differences in memories about childhood due to the passage of time) and across countries (e.g., cultural differences in memory processing), which should be considered when interpreting the findings of this study. However, for recall bias to completely explain away the observed associations would require that the effect of forgivingness in adulthood on biasing retrospective assessments of the childhood predictors is at least as strong as the observed associations themselves (VanderWeele & Li, 2019). To mitigate sources of bias, future research might employ prospective longitudinal designs that follow individuals from childhood to adulthood and complement self-report survey responses with data derived from other sources (e.g., parents).

Second, all psychosocial constructs (including the outcome of forgivingness) were assessed using a single item. While it is somewhat common to use single-item measures in large-scale epidemiologic studies and allows for a wider range of constructs to be assessed in a single survey, conceptual coverage of the construct tends to be narrower compared to multi-item measures. Future research could strengthen the findings of this study by using measures that provide broader conceptual coverage of the psychosocial constructs that were assessed.

Third, we cannot rule out the possibility that some of the observed associations might be explained away by unmeasured confounding. *E*-values generally suggested that some of the observed associations which did not include the null might be somewhat robust to unmeasured confounding, but the set of predictors may not have provided adequate confounding control. Although decisions about the inclusion of predictors were constrained by the variables that were measured in the GFS survey and multicollinearity issues, future studies could strengthen causal claims by including a more comprehensive set of relevant covariates. Relatedly, candidate childhood predictors attended principally to the microsystem of a child’s socioecological system. While factors nested within layers that are more proximal to the child (i.e., the microsystem) are often prioritized because they tend to be more directly involved in shaping development, additional work is needed to determine how different socioecological layers (particularly those that are more distal) during childhood might influence the development of a forgiving disposition.

Fourth, there is a chance that certain predictors might be on the pathway (a mediator) between another predictor and the outcome. For example, if the quality of relationships with both parents when growing up is downstream of family structure, it is possible that the observed associations with forgivingness in adulthood for one or more categories of family structure might be conservative estimates of actual overall effects.

Fifth, while Wave 1 of the GFS includes a substantially diverse set of 22 countries that collectively represent roughly half of the world’s population, some cultures and contexts are not represented in the dataset. We recommend caution if attempting to generalize the findings beyond the countries in the analytic sample and encourage research in countries that were not represented in Wave 1 of the GFS.

Sixth, this study allowed us to examine fundamental associations between a range of candidate predictors and subsequent forgivingness in adulthood, but cannot confirm why such associations might exist. As additional waves of the GFS data become available, researchers may be able to explore possible mechanisms underlying the associations that were observed.

## Conclusion

The present multinational study employed a life course epidemiological approach to explore 13 individual characteristics and recalled childhood factors as candidate predictors of forgivingness in adulthood. The findings provided some evidence suggesting that a combination of individual characteristics, early life conditions or experiences, and social circumstances or influences within a child’s system of socioecology are associated with forgivingness in adulthood (both within and across the countries). Although additional research is needed to build on the findings of this study, especially in identifying potential reasons for associations that differed across countries, the evidence reported herein could have practical utility for shaping multipronged early-life interventions that support the disposition to forgive.

## Electronic Supplementary Material

Below is the link to the electronic supplementary material.

Supplementary Material 1

## Data Availability

The data are publicly available through the Center for Open Science (https://www.cos.io/gfs).
