# Supplementary material for: Childhood Predictors of Dispositional Forgivingness in Adulthood: A Cross-National Analysis with 22 Countries
Source: Appl Res Qual Life. 2025 May 7;20(3):1057–84. doi: 10.1007/s11482-025-10451-z (PMC12328535; doi:10.1007/s11482-025-10451-z)

Childhood Predictors of Dispositional Forgivingness in Adulthood: A Cross-National Analysis With 22 Countries

**SUPPLEMENTAL FILE**

Tables S1a-S22a. Nationally Representative Descriptive Statistics for Each Country.

Tables S1b-S22b. Associations of Candidate Predictors With Forgivingness in Adulthood for Each Country.

Table S23. Population Weighted Meta-Analyses for Associations of Candidate Predictors With Forgivingness in Adulthood.

Table S24. Random Effects Meta-Analyses for Associations of Candidate Predictors With Forgivingness in Adulthood Using Alternative Coding of Forgivingness.

Figures S1-S27. Forest Plot for the Association of Each Candidate Predictor Category With Forgivingness in Adulthood in All 22 Countries.

| Table S1a  *Nationally Representative Descriptive Statistics for Argentina (N = 6,724)* | |
| --- | --- |
| Characteristic | *n* (%) |
| **Relationship with mother when growing up** |  |
| Very good | 4,463 (66%) |
| Somewhat good | 1,436 (21%) |
| Somewhat bad | 299 (4.4%) |
| Very bad | 216 (3.2%) |
| Does not apply | 273 (4.1%) |
| Missing | 36 (0.5%) |
| **Relationship with father when growing up** |  |
| Very good | 3,612 (54%) |
| Somewhat good | 1,537 (23%) |
| Somewhat bad | 440 (6.5%) |
| Very bad | 401 (6.0%) |
| Does not apply | 694 (10%) |
| Missing | 39 (0.6%) |
| **Family structure around age 12** |  |
| Parents were married | 4,110 (61%) |
| Parents were divorced | 637 (9.5%) |
| Parents were never married | 1,368 (20%) |
| One or both parents had died | 199 (3.0%) |
| Missing | 410 (6.1%) |
| **Subjective financial status of family around age 12** |  |
| Lived comfortably | 2,042 (30%) |
| Got by | 2,305 (34%) |
| Found it difficult | 1,789 (27%) |
| Found it very difficult | 569 (8.5%) |
| Missing | 19 (0.3%) |
| **Experienced abuse when growing up** |  |
| Yes | 1,302 (19%) |
| No | 5,271 (78%) |
| Missing | 151 (2.2%) |
| **Felt like an outsider in the family when growing up** |  |
| Yes | 1,165 (17%) |
| No | 5,458 (81%) |
| Missing | 101 (1.5%) |
| **Self-rated health when growing up** |  |
| Excellent | 2,402 (36%) |
| Very good | 1,819 (27%) |
| Good | 1,830 (27%) |
| Fair | 505 (7.5%) |
| Poor | 156 (2.3%) |
| Missing | 12 (0.2%) |
| **Immigration status** |  |
| Born in this country | 6,346 (94%) |
| Born in another country | 348 (5.2%) |
| Missing | 29 (0.4%) |
| **Frequency of religious service attendance around age 12** |  |
| ≥ 1/week | 2,601 (39%) |
| 1-3/month | 1,204 (18%) |
| < 1/month | 1,059 (16%) |
| Never | 1,808 (27%) |
| Missing | 53 (0.8%) |
| **Year of birth** |  |
| 1998-2005 (current age: 18-24 years) | 1,108 (16%) |
| 1993-1998 (current age: 25-29 years) | 719 (11%) |
| 1983-1993 (current age: 30-39 years) | 1,432 (21%) |
| 1973-1983 (current age: 40-49 years) | 1,254 (19%) |
| 1963-1973 (current age: 50-59 years) | 1,014 (15%) |
| 1953-1963 (current age: 60-69 years) | 730 (11%) |
| 1943-1953 (current age: 70-79 years) | 356 (5.3%) |
| 1943 or earlier (current age: 80+ years) | 112 (1.7%) |
| Missing | 0 (0%) |
| **Gender** |  |
| Male | 3,143 (47%) |
| Female | 3,542 (53%) |
| Other | 21 (0.3%) |
| Missing | 18 (0.3%) |
| **Religious affiliation at age 12** |  |
| Buddhism | 3 (< 0.1%) |
| Christianity | 5,805 (86%) |
| Hinduism | 2 (< 0.1%) |
| Islam | 11 (0.2%) |
| Judaism | 51 (0.8%) |
| No religion/atheist/agnostic | 697 (10%) |
| Primal, animist, or folk religion | 17 (0.2%) |
| Sikhism | 5 (< 0.1%) |
| Some other religion | 10 (0.2%) |
| Taoism | 1 (< 0.1%) |
| Missing | 122 (1.8%) |
| **Racial/ethnic identity** |  |
| Asian | 43 (0.6%) |
| Black | 95 (1.4%) |
| Indigenous | 129 (1.9%) |
| Mestizo(a) | 1,801 (27%) |
| Mullato(a) | 75 (1.1%) |
| Other | 104 (1.5%) |
| White | 3,406 (51%) |
| Missing | 1,070 (16%) |

| Table S1b  *Regression of Dispositional Forgivingness on Candidate Predictors for Argentina* | | | | |
| --- | --- | --- | --- | --- |
| Variable | RR (95% CI) | Global  *p*-value | *E*-values | |
|  |  |  | *E*-value for estimate | *E*-value for 95% CI |
| **Relationship with mother when growing up** |  | < 0.001 |  |  |
| Ref: Very bad/somewhat bad | - |  | - | - |
| Very good/somewhat good | 1.07 (0.98, 1.18) |  | 1.35 | 1.00 |
| **Relationship with father when growing up** |  | 0.010 |  |  |
| Ref: Very bad/somewhat bad | - |  | - | - |
| Very good/somewhat good | 0.97 (0.90, 1.03) |  | 1.23 | 1.00 |
| **Family structure around age 12** |  | 0.113 |  |  |
| Ref: Parents were married | - |  | - | - |
| Parents were divorced | 1.03 (0.96, 1.11) |  | 1.21 | 1.00 |
| Parents were never married | 0.95 (0.89, 1.02) |  | 1.28 | 1.00 |
| One or both parents had died | 0.96 (0.84, 1.09) |  | 1.25 | 1.00 |
| **Subjective financial status of family around age 12** |  | 0.814 |  |  |
| Ref: Got by | - |  | - | - |
| Lived comfortably | 0.99 (0.94, 1.05) |  | 1.10 | 1.00 |
| Found it difficult | 1.00 (0.94, 1.06) |  | 1.06 | 1.00 |
| Found it very difficult | 1.05 (0.97, 1.15) |  | 1.30 | 1.00 |
| **Experienced abuse when growing up** |  | 0.998 |  |  |
| Ref: No | - |  | - | - |
| Yes | 1.01 (0.95, 1.06) |  | 1.08 | 1.00 |
| **Felt like an outsider in the family when growing up** |  | 0.684 |  |  |
| Ref: No | - |  | - | - |
| Yes | 0.98 (0.91, 1.05) |  | 1.18 | 1.00 |
| **Self-rated health when growing up** |  | 0.017 |  |  |
| Ref: Good | - |  | - | - |
| Excellent | 1.07 (1.01, 1.14) |  | 1.36 | 1.14 |
| Very good | 1.04 (0.98, 1.11) |  | 1.26 | 1.00 |
| Fair | 1.03 (0.94, 1.13) |  | 1.21 | 1.00 |
| Poor | 0.92 (0.75, 1.14) |  | 1.38 | 1.00 |
| **Immigration status** |  | 0.002 |  |  |
| Ref: Born in this country | - |  | - | - |
| Born in another country | 1.08 (0.99, 1.19) |  | 1.39 | 1.00 |
| **Frequency of religious service attendance around age 12** |  | 0.034 |  |  |
| Ref: Never | - |  | - | - |
| ≥ 1/week | 1.07 (1.01, 1.14) |  | 1.34 | 1.09 |
| 1-3/month | 1.03 (0.96, 1.11) |  | 1.20 | 1.00 |
| < 1/month | 1.03 (0.96, 1.11) |  | 1.21 | 1.00 |
| **Year of birth** |  | 0.047 |  |  |
| Ref: 1998-2005 (current age: 18-24 years) | - |  | - | - |
| 1993-1998 (current age: 25-29 years) | 1.11 (1.01, 1.21) |  | 1.45 | 1.11 |
| 1983-1993 (current age: 30-39 years) | 1.06 (0.98, 1.16) |  | 1.32 | 1.00 |
| 1973-1983 (current age: 40-49 years) | 1.11 (1.02, 1.20) |  | 1.45 | 1.17 |
| 1963-1973 (current age: 50-59 years) | 1.09 (1.01, 1.19) |  | 1.42 | 1.08 |
| 1953-1963 (current age: 60-69 years) | 1.09 (0.99, 1.20) |  | 1.40 | 1.00 |
| 1943-1953 (current age: 70-79 years) | 1.09 (0.97, 1.23) |  | 1.41 | 1.00 |
| 1943 or earlier (current age: 80+ years) | 0.88 (0.68, 1.13) |  | 1.55 | 1.00 |
| **Gender** |  | < 0.001 |  |  |
| Ref: Male | - |  | - | - |
| Female | 1.08 (1.04, 1.13) |  | 1.39 | 1.24 |
| Other | 1.36 (1.14, 1.64) |  | 2.08 | 1.55 |
| **Religious affiliation at age 12** |  | 0.997 |  |  |
| Ref: No religion/atheist/agnostic | - |  | - | - |
| Christianity | 0.98 (0.90, 1.06) |  | 1.18 | 1.00 |
| Some other religion | 0.99 (0.83, 1.19) |  | 1.08 | 1.00 |
| **Racial/ethnic identity** |  | 1.000 |  |  |
| Ref: Majority | - |  | - | - |
| Minority | 1.00 (0.96, 1.05) |  | 1.03 | 1.00 |

| Table S2a  *Nationally Representative Descriptive Statistics for Australia (N = 3,844)* | |
| --- | --- |
| Characteristic | *n* (%) |
| **Relationship with mother when growing up** |  |
| Very good | 2,554 (66%) |
| Somewhat good | 925 (24%) |
| Somewhat bad | 218 (5.7%) |
| Very bad | 107 (2.8%) |
| Does not apply | 32 (0.8%) |
| Missing | 7 (0.2%) |
| **Relationship with father when growing up** |  |
| Very good | 2,032 (53%) |
| Somewhat good | 1,144 (30%) |
| Somewhat bad | 315 (8.2%) |
| Very bad | 196 (5.1%) |
| Does not apply | 148 (3.9%) |
| Missing | 9 (0.2%) |
| **Family structure around age 12** |  |
| Parents were married | 3,048 (79%) |
| Parents were divorced | 462 (12%) |
| Parents were never married | 187 (4.9%) |
| One or both parents had died | 96 (2.5%) |
| Missing | 52 (1.4%) |
| **Subjective financial status of family around age 12** |  |
| Lived comfortably | 1,756 (46%) |
| Got by | 1,496 (39%) |
| Found it difficult | 422 (11%) |
| Found it very difficult | 154 (4.0%) |
| Missing | 16 (0.4%) |
| **Experienced abuse when growing up** |  |
| Yes | 995 (26%) |
| No | 2,790 (73%) |
| Missing | 59 (1.5%) |
| **Felt like an outsider in the family when growing up** |  |
| Yes | 756 (20%) |
| No | 3,062 (80%) |
| Missing | 26 (0.7%) |
| **Self-rated health when growing up** |  |
| Excellent | 1,736 (45%) |
| Very good | 1,087 (28%) |
| Good | 603 (16%) |
| Fair | 308 (8.0%) |
| Poor | 106 (2.8%) |
| Missing | 4 (< 0.1%) |
| **Immigration status** |  |
| Born in this country | 2,953 (77%) |
| Born in another country | 885 (23%) |
| Missing | 6 (0.2%) |
| **Frequency of religious service attendance around age 12** |  |
| ≥ 1/week | 1,362 (35%) |
| 1-3/month | 486 (13%) |
| < 1/month | 600 (16%) |
| Never | 1,307 (34%) |
| Missing | 90 (2.3%) |
| **Year of birth** |  |
| 1998-2005 (current age: 18-24 years) | 345 (9.0%) |
| 1993-1998 (current age: 25-29 years) | 282 (7.3%) |
| 1983-1993 (current age: 30-39 years) | 641 (17%) |
| 1973-1983 (current age: 40-49 years) | 618 (16%) |
| 1963-1973 (current age: 50-59 years) | 691 (18%) |
| 1953-1963 (current age: 60-69 years) | 589 (15%) |
| 1943-1953 (current age: 70-79 years) | 498 (13%) |
| 1943 or earlier (current age: 80+ years) | 178 (4.6%) |
| Missing | 2 (< 0.1%) |
| **Gender** |  |
| Male | 1,861 (48%) |
| Female | 1,941 (50%) |
| Other | 36 (0.9%) |
| Missing | 6 (0.2%) |
| **Religious affiliation at age 12** |  |
| Baha’i | 5 (0.1%) |
| Buddhism | 16 (0.4%) |
| Christianity | 2,678 (70%) |
| Hinduism | 39 (1.0%) |
| Islam | 48 (1.2%) |
| Judaism | 29 (0.8%) |
| No religion/atheist/agnostic | 990 (26%) |
| Primal, animist, or folk religion | 4 (< 0.1%) |
| Sikhism | 6 (0.2%) |
| Some other religion | 8 (0.2%) |
| Taoism | 1 (< 0.1%) |
| Missing | 21 (0.5%) |
| **Racial/ethnic identity** |  |
| Aboriginal | 53 (1.4%) |
| Australian | 1,946 (51%) |
| Australian British/European | 1,047 (27%) |
| Chinese | 75 (1.9%) |
| Indian | 58 (1.5%) |
| Japanese | 1 (< 0.1%) |
| Malay | 11 (0.3%) |
| New Zealander | 91 (2.4%) |
| Other | 163 (4.2%) |
| Other European | 357 (9.3%) |
| Russian | 7 (0.2%) |
| Samoan | 4 (0.1%) |
| Sinhalese | 1 (< 0.1%) |
| Spanish | 2 (< 0.1%) |
| Sri Lankan Moor | 1 (< 0.1%) |
| Sri Lankan Tamil | 7 (0.2%) |
| Vietnamese | 7 (0.2%) |
| Missing | 14 (0.4%) |

| Table S2b  *Regression of Dispositional Forgivingness On Candidate Predictors for Australia* | | | | |
| --- | --- | --- | --- | --- |
| Variable | RR (95% CI) | Global  *p*-value | *E*-values | |
|  |  |  | *E*-value for estimate | *E*-value for 95% CI |
| **Relationship with mother when growing up** |  | 0.616 |  |  |
| Ref: Very bad/somewhat bad | - |  | - | - |
| Very good/somewhat good | 1.02 (0.93, 1.12) |  | 1.17 | 1.00 |
| **Relationship with father when growing up** |  | < 0.001 |  |  |
| Ref: Very bad/somewhat bad | - |  | - | - |
| Very good/somewhat good | 1.05 (0.97, 1.14) |  | 1.29 | 1.00 |
| **Family structure around age 12** |  | 0.204 |  |  |
| Ref: Parents were married | - |  | - | - |
| Parents were divorced | 0.99 (0.90, 1.08) |  | 1.13 | 1.00 |
| Parents were never married | 1.04 (0.93, 1.18) |  | 1.26 | 1.00 |
| One or both parents had died | 1.13 (0.98, 1.29) |  | 1.49 | 1.00 |
| **Subjective financial status of family around age 12** |  | 0.607 |  |  |
| Ref: Got by | - |  | - | - |
| Lived comfortably | 1.01 (0.97, 1.06) |  | 1.13 | 1.00 |
| Found it difficult | 0.96 (0.88, 1.04) |  | 1.26 | 1.00 |
| Found it very difficult | 1.05 (0.93, 1.19) |  | 1.29 | 1.00 |
| **Experienced abuse when growing up** |  | 0.151 |  |  |
| Ref: No | - |  | - | - |
| Yes | 1.03 (0.98, 1.09) |  | 1.21 | 1.00 |
| **Felt like an outsider in the family when growing up** |  | 0.622 |  |  |
| Ref: No | - |  | - | - |
| Yes | 0.97 (0.91, 1.04) |  | 1.19 | 1.00 |
| **Self-rated health when growing up** |  | 0.287 |  |  |
| Ref: Good | - |  | - | - |
| Excellent | 1.05 (0.99, 1.13) |  | 1.30 | 1.00 |
| Very good | 1.01 (0.94, 1.08) |  | 1.11 | 1.00 |
| Fair | 1.02 (0.92, 1.13) |  | 1.16 | 1.00 |
| Poor | 0.98 (0.81, 1.17) |  | 1.18 | 1.00 |
| **Immigration status** |  | 0.115 |  |  |
| Ref: Born in this country | - |  | - | - |
| Born in another country | 1.03 (0.98, 1.09) |  | 1.22 | 1.00 |
| **Frequency of religious service attendance around age 12** |  | < 0.001 |  |  |
| Ref: Never | - |  | - | - |
| ≥ 1/week | 1.13 (1.06, 1.20) |  | 1.50 | 1.30 |
| 1-3/month | 1.15 (1.07, 1.23) |  | 1.56 | 1.34 |
| < 1/month | 1.07 (0.99, 1.15) |  | 1.35 | 1.00 |
| **Year of birth** |  | < 0.001 |  |  |
| Ref: 1998-2005 (current age: 18-24 years) | - |  | - | - |
| 1993-1998 (current age: 25-29 years) | 0.99 (0.89, 1.11) |  | 1.11 | 1.00 |
| 1983-1993 (current age: 30-39 years) | 0.91 (0.83, 1.01) |  | 1.42 | 1.00 |
| 1973-1983 (current age: 40-49 years) | 0.89 (0.81, 0.98) |  | 1.49 | 1.15 |
| 1963-1973 (current age: 50-59 years) | 0.91 (0.83, 1.00) |  | 1.41 | 1.00 |
| 1953-1963 (current age: 60-69 years) | 0.90 (0.81, 0.98) |  | 1.49 | 1.16 |
| 1943-1953 (current age: 70-79 years) | 0.98 (0.89, 1.07) |  | 1.18 | 1.00 |
| 1943 or earlier (current age: 80+ years) | 0.98 (0.88, 1.10) |  | 1.14 | 1.00 |
| **Gender** |  | < 0.001 |  |  |
| Ref: Male | - |  | - | - |
| Female | 1.05 (1.01, 1.10) |  | 1.28 | 1.08 |
| Other | 1.36 (1.22, 1.52) |  | 2.06 | 1.73 |
| **Religious affiliation at age 12** |  | 0.343 |  |  |
| Ref: No religion/atheist/agnostic | - |  | - | - |
| Christianity | 1.02 (0.96, 1.10) |  | 1.18 | 1.00 |
| Some other religion | 0.94 (0.83, 1.07) |  | 1.32 | 1.00 |
| **Racial/ethnic identity** |  | 0.973 |  |  |
| Ref: Majority | - |  | - | - |
| Minority | 1.01 (0.96, 1.06) |  | 1.10 | 1.00 |

| Table S3a  *Nationally Representative Descriptive Statistics for Brazil (N = 13,204)* | |
| --- | --- |
| Characteristic | *n* (%) |
| **Relationship with mother when growing up** |  |
| Very good | 8,369 (63%) |
| Somewhat good | 3,559 (27%) |
| Somewhat bad | 483 (3.7%) |
| Very bad | 214 (1.6%) |
| Does not apply | 507 (3.8%) |
| Missing | 73 (0.6%) |
| **Relationship with father when growing up** |  |
| Very good | 6,364 (48%) |
| Somewhat good | 3,654 (28%) |
| Somewhat bad | 1,035 (7.8%) |
| Very bad | 756 (5.7%) |
| Does not apply | 1,303 (9.9%) |
| Missing | 93 (0.7%) |
| **Family structure around age 12** |  |
| Parents were married | 8,546 (65%) |
| Parents were divorced | 1,384 (10%) |
| Parents were never married | 1,985 (15%) |
| One or both parents had died | 508 (3.8%) |
| Missing | 781 (5.9%) |
| **Subjective financial status of family around age 12** |  |
| Lived comfortably | 4,998 (38%) |
| Got by | 4,616 (35%) |
| Found it difficult | 2,484 (19%) |
| Found it very difficult | 1,027 (7.8%) |
| Missing | 79 (0.6%) |
| **Experienced abuse when growing up** |  |
| Yes | 2,606 (20%) |
| No | 10,147 (77%) |
| Missing | 451 (3.4%) |
| **Felt like an outsider in the family when growing up** |  |
| Yes | 1,659 (13%) |
| No | 11,234 (85%) |
| Missing | 311 (2.4%) |
| **Self-rated health when growing up** |  |
| Excellent | 5,312 (40%) |
| Very good | 3,392 (26%) |
| Good | 2,873 (22%) |
| Fair | 1,368 (10%) |
| Poor | 228 (1.7%) |
| Missing | 30 (0.2%) |
| **Immigration status** |  |
| Born in this country | 12,688 (96%) |
| Born in another country | 153 (1.2%) |
| Missing | 363 (2.7%) |
| **Frequency of religious service attendance around age 12** |  |
| ≥ 1/week | 6,306 (48%) |
| 1-3/month | 2,491 (19%) |
| < 1/month | 2,629 (20%) |
| Never | 1,707 (13%) |
| Missing | 71 (0.5%) |
| **Year of birth** |  |
| 1998-2005 (current age: 18-24 years) | 1,986 (15%) |
| 1993-1998 (current age: 25-29 years) | 1,468 (11%) |
| 1983-1993 (current age: 30-39 years) | 2,908 (22%) |
| 1973-1983 (current age: 40-49 years) | 2,638 (20%) |
| 1963-1973 (current age: 50-59 years) | 2,131 (16%) |
| 1953-1963 (current age: 60-69 years) | 1,435 (11%) |
| 1943-1953 (current age: 70-79 years) | 510 (3.9%) |
| 1943 or earlier (current age: 80+ years) | 126 (1.0%) |
| Missing | 0 (0%) |
| **Gender** |  |
| Male | 6,320 (48%) |
| Female | 6,820 (52%) |
| Other | 35 (0.3%) |
| Missing | 30 (0.2%) |
| **Religious affiliation at age 12** |  |
| Baha’i | 1 (< 0.1%) |
| Buddhism | 27 (0.2%) |
| Christianity | 11,403 (86%) |
| Confucianism | 7 (< 0.1%) |
| Hinduism | 1 (< 0.1%) |
| Islam | 15 (0.1%) |
| Jainism | 4 (< 0.1%) |
| Judaism | 40 (0.3%) |
| No religion/atheist/agnostic | 908 (6.9%) |
| Primal, animist, or folk religion | 17 (0.1%) |
| Shinto | 4 (< 0.1%) |
| Some other religion | 87 (0.7%) |
| Spiritism | 336 (2.5%) |
| Taoism | 1 (< 0.1%) |
| Umbanda, Candomblé, and other African-derived religions | 262 (2.0%) |
| Missing | 94 (0.7%) |
| **Racial/ethnic identity** |  |
| Amarela | 238 (1.8%) |
| Branca | 5,169 (39%) |
| Indígena | 131 (1.0%) |
| Other | 61 (0.5%) |
| Parda | 5,125 (39%) |
| Preta | 1,615 (12%) |
| Missing | 865 (6.6%) |

| Table S3b  *Regression of Dispositional Forgivingness on Candidate Predictors for Brazil* | | | | |
| --- | --- | --- | --- | --- |
| Variable | RR (95% CI) | Global  *p*-value | *E*-values | |
|  |  |  | *E*-value for estimate | *E*-value for 95% CI |
| **Relationship with mother when growing up** |  | < 0.001 |  |  |
| Ref: Very bad/somewhat bad | - |  | - | - |
| Very good/somewhat good | 1.15 (1.08, 1.23) |  | 1.57 | 1.37 |
| **Relationship with father when growing up** |  | < 0.001 |  |  |
| Ref: Very bad/somewhat bad | - |  | - | - |
| Very good/somewhat good | 1.09 (1.04, 1.14) |  | 1.41 | 1.25 |
| **Family structure around age 12** |  | 0.994 |  |  |
| Ref: Parents were married | - |  | - | - |
| Parents were divorced | 0.99 (0.94, 1.04) |  | 1.12 | 1.00 |
| Parents were never married | 0.99 (0.95, 1.04) |  | 1.09 | 1.00 |
| One or both parents had died | 0.97 (0.88, 1.06) |  | 1.23 | 1.00 |
| **Subjective financial status of family around age 12** |  | 0.055 |  |  |
| Ref: Got by | - |  | - | - |
| Lived comfortably | 0.98 (0.95, 1.01) |  | 1.17 | 1.00 |
| Found it difficult | 1.02 (0.98, 1.06) |  | 1.16 | 1.00 |
| Found it very difficult | 1.02 (0.96, 1.08) |  | 1.17 | 1.00 |
| **Experienced abuse when growing up** |  | 0.002 |  |  |
| Ref: No | - |  | - | - |
| Yes | 1.03 (1.00, 1.07) |  | 1.23 | 1.00 |
| **Felt like an outsider in the family when growing up** |  | 0.266 |  |  |
| Ref: No | - |  | - | - |
| Yes | 0.98 (0.93, 1.02) |  | 1.18 | 1.00 |
| **Self-rated health when growing up** |  | < 0.001 |  |  |
| Ref: Good | - |  | - | - |
| Excellent | 1.05 (1.01, 1.09) |  | 1.27 | 1.09 |
| Very good | 1.02 (0.98, 1.06) |  | 1.15 | 1.00 |
| Fair | 0.97 (0.91, 1.03) |  | 1.22 | 1.00 |
| Poor | 0.93 (0.81, 1.07) |  | 1.35 | 1.00 |
| **Immigration status** |  | 0.999 |  |  |
| Ref: Born in this country | - |  | - | - |
| Born in another country | 1.01 (0.86, 1.18) |  | 1.10 | 1.00 |
| **Frequency of religious service attendance around age 12** |  | < 0.001 |  |  |
| Ref: Never | - |  | - | - |
| ≥ 1/week | 1.14 (1.08, 1.21) |  | 1.55 | 1.39 |
| 1-3/month | 1.08 (1.02, 1.15) |  | 1.37 | 1.14 |
| < 1/month | 1.05 (0.99, 1.12) |  | 1.28 | 1.00 |
| **Year of birth** |  | < 0.001 |  |  |
| Ref: 1998-2005 (current age: 18-24 years) | - |  | - | - |
| 1993-1998 (current age: 25-29 years) | 1.03 (0.98, 1.10) |  | 1.23 | 1.00 |
| 1983-1993 (current age: 30-39 years) | 1.06 (1.01, 1.12) |  | 1.33 | 1.13 |
| 1973-1983 (current age: 40-49 years) | 1.13 (1.07, 1.19) |  | 1.51 | 1.35 |
| 1963-1973 (current age: 50-59 years) | 1.15 (1.09, 1.22) |  | 1.57 | 1.41 |
| 1953-1963 (current age: 60-69 years) | 1.17 (1.10, 1.25) |  | 1.63 | 1.44 |
| 1943-1953 (current age: 70-79 years) | 1.13 (1.02, 1.25) |  | 1.51 | 1.18 |
| 1943 or earlier (current age: 80+ years) | 1.22 (1.04, 1.42) |  | 1.73 | 1.25 |
| **Gender** |  | 0.994 |  |  |
| Ref: Male | - |  | - | - |
| Female | 1.00 (0.97, 1.03) |  | 1.02 | 1.00 |
| Other | 0.88 (0.61, 1.27) |  | 1.52 | 1.00 |
| **Religious affiliation at age 12** |  | 0.373 |  |  |
| Ref: No religion/atheist/agnostic | - |  | - | - |
| Christianity | 0.99 (0.93, 1.05) |  | 1.09 | 1.00 |
| Some other religion | 0.95 (0.87, 1.03) |  | 1.30 | 1.00 |
| **Racial/ethnic identity** |  | 1.000 |  |  |
| Ref: Majority | - |  | - | - |
| Minority | 1.00 (0.97, 1.03) |  | 1.01 | 1.00 |

| Table S4a  *Nationally Representative Descriptive Statistics for Egypt (N = 4,729)* | |
| --- | --- |
| Characteristic | *n* (%) |
| **Relationship with mother when growing up** |  |
| Very good | 4,110 (87%) |
| Somewhat good | 505 (11%) |
| Somewhat bad | 21 (0.4%) |
| Very bad | 10 (0.2%) |
| Does not apply | 83 (1.8%) |
| Missing | 0 (0%) |
| **Relationship with father when growing up** |  |
| Very good | 3,713 (79%) |
| Somewhat good | 683 (14%) |
| Somewhat bad | 56 (1.2%) |
| Very bad | 30 (0.6%) |
| Does not apply | 233 (4.9%) |
| Missing | 14 (0.3%) |
| **Family structure around age 12** |  |
| Parents were married | 4,049 (86%) |
| Parents were divorced | 131 (2.8%) |
| Parents were never married | 9 (0.2%) |
| One or both parents had died | 485 (10%) |
| Missing | 55 (1.2%) |
| **Subjective financial status of family around age 12** |  |
| Lived comfortably | 1,251 (26%) |
| Got by | 2,352 (50%) |
| Found it difficult | 857 (18%) |
| Found it very difficult | 268 (5.7%) |
| Missing | 1 (< 0.1%) |
| **Experienced abuse when growing up** |  |
| Yes | 405 (8.6%) |
| No | 4,293 (91%) |
| Missing | 30 (0.6%) |
| **Felt like an outsider in the family when growing up** |  |
| Yes | 260 (5.5%) |
| No | 4,456 (94%) |
| Missing | 13 (0.3%) |
| **Self-rated health when growing up** |  |
| Excellent | 2,687 (57%) |
| Very good | 1,174 (25%) |
| Good | 497 (11%) |
| Fair | 265 (5.6%) |
| Poor | 106 (2.2%) |
| Missing | 1 (< 0.1%) |
| **Immigration status** |  |
| Born in this country | 4,713 (100%) |
| Born in another country | 16 (0.3%) |
| Missing | 1 (< 0.1%) |
| **Frequency of religious service attendance around age 12** |  |
| ≥ 1/week | 2,307 (49%) |
| 1-3/month | 570 (12%) |
| < 1/month | 629 (13%) |
| Never | 1,165 (25%) |
| Missing | 57 (1.2%) |
| **Year of birth** |  |
| 1998-2005 (current age: 18-24 years) | 960 (20%) |
| 1993-1998 (current age: 25-29 years) | 607 (13%) |
| 1983-1993 (current age: 30-39 years) | 1,204 (25%) |
| 1973-1983 (current age: 40-49 years) | 897 (19%) |
| 1963-1973 (current age: 50-59 years) | 613 (13%) |
| 1953-1963 (current age: 60-69 years) | 387 (8.2%) |
| 1943-1953 (current age: 70-79 years) | 54 (1.1%) |
| 1943 or earlier (current age: 80+ years) | 7 (0.2%) |
| Missing | 0 (0%) |
| **Gender** |  |
| Male | 2,394 (51%) |
| Female | 2,334 (49%) |
| Other | 0 (0%) |
| Missing | 0 (< 0.1%) |
| **Religious affiliation at age 12** |  |
| Christianity | 123 (2.6%) |
| Islam | 4,602 (97%) |
| Jainism | 1 (< 0.1%) |
| Taoism | 0 (< 0.1%) |
| Missing | 3 (< 0.1%) |
| **Racial/ethnic identity** |  |
| Arab | 4,585 (97%) |
| Bedouin Arab | 4 (< 0.1%) |
| Greek | 1 (< 0.1%) |
| Nubian | 27 (0.6%) |
| Turkish | 9 (0.2%) |
| Missing | 102 (2.2%) |

| Table S4b  *Regression of Dispositional Forgivingness on Candidate Predictors for Egypt* | | | | |
| --- | --- | --- | --- | --- |
| Variable | RR (95% CI) | Global  *p*-value | *E*-values | |
|  |  |  | *E*-value for estimate | *E*-value for 95% CI |
| **Relationship with mother when growing up** |  | 0.422 |  |  |
| Ref: Very bad/somewhat bad | - |  | - | - |
| Very good/somewhat good | 0.98 (0.90, 1.07) |  | 1.16 | 1.00 |
| **Relationship with father when growing up** |  | < 0.001 |  |  |
| Ref: Very bad/somewhat bad | - |  | - | - |
| Very good/somewhat good | 1.08 (0.97, 1.20) |  | 1.37 | 1.00 |
| **Family structure around age 12** |  | 0.773 |  |  |
| Ref: Parents were married | - |  | - | - |
| Parents were divorced | 0.99 (0.89, 1.10) |  | 1.11 | 1.00 |
| Parents were never married | 0.85 (0.69, 1.05) |  | 1.63 | 1.00 |
| One or both parents had died | 1.00 (0.95, 1.05) |  | 1.07 | 1.00 |
| **Subjective financial status of family around age 12** |  | 0.428 |  |  |
| Ref: Got by | - |  | - | - |
| Lived comfortably | 1.00 (0.96, 1.03) |  | 1.05 | 1.00 |
| Found it difficult | 0.98 (0.94, 1.03) |  | 1.15 | 1.00 |
| Found it very difficult | 1.04 (0.99, 1.10) |  | 1.25 | 1.00 |
| **Experienced abuse when growing up** |  | 0.552 |  |  |
| Ref: No | - |  | - | - |
| Yes | 0.98 (0.93, 1.03) |  | 1.17 | 1.00 |
| **Felt like an outsider in the family when growing up** |  | 0.168 |  |  |
| Ref: No | - |  | - | - |
| Yes | 0.97 (0.92, 1.02) |  | 1.22 | 1.00 |
| **Self-rated health when growing up** |  | 0.012 |  |  |
| Ref: Good | - |  | - | - |
| Excellent | 1.03 (0.97, 1.08) |  | 1.20 | 1.00 |
| Very good | 1.05 (0.99, 1.11) |  | 1.28 | 1.00 |
| Fair | 0.98 (0.90, 1.07) |  | 1.16 | 1.00 |
| Poor | 1.07 (0.98, 1.16) |  | 1.34 | 1.00 |
| **Immigration status** |  | 0.284 |  |  |
| Ref: Born in this country | - |  | - | - |
| Born in another country | 1.08 (0.93, 1.28) |  | 1.40 | 1.00 |
| **Frequency of religious service attendance around age 12** |  | 0.735 |  |  |
| Ref: Never | - |  | - | - |
| ≥ 1/week | 0.99 (0.96, 1.02) |  | 1.11 | 1.00 |
| 1-3/month | 1.01 (0.96, 1.05) |  | 1.10 | 1.00 |
| < 1/month | 1.02 (0.98, 1.07) |  | 1.16 | 1.00 |
| **Year of birth** |  | < 0.001 |  |  |
| Ref: 1998-2005 (current age: 18-24 years) | - |  | - | - |
| 1993-1998 (current age: 25-29 years) | 1.05 (0.99, 1.12) |  | 1.28 | 1.00 |
| 1983-1993 (current age: 30-39 years) | 1.08 (1.03, 1.13) |  | 1.38 | 1.22 |
| 1973-1983 (current age: 40-49 years) | 1.13 (1.07, 1.18) |  | 1.50 | 1.35 |
| 1963-1973 (current age: 50-59 years) | 1.15 (1.09, 1.21) |  | 1.56 | 1.42 |
| 1953-1963 (current age: 60-69 years) | 1.14 (1.08, 1.21) |  | 1.55 | 1.37 |
| 1943-1953 (current age: 70-79 years) | 1.22 (1.16, 1.29) |  | 1.75 | 1.58 |
| 1943 or earlier (current age: 80+ years) | 0.79 (0.35, 1.80) |  | 1.82 | 1.00 |
| **Gender** |  | 0.804 |  |  |
| Ref: Male | - |  | - | - |
| Female | 0.99 (0.96, 1.02) |  | 1.11 | 1.00 |
| **Religious affiliation at age 12** |  | 0.983 |  |  |
| Ref: Islam | - |  | - | - |
| Some other religion | 0.99 (0.90, 1.08) |  | 1.14 | 1.00 |
| **Racial/ethnic identity** |  | 0.989 |  |  |
| Ref: Majority | - |  | - | - |
| Minority | 0.98 (0.83, 1.15) |  | 1.18 | 1.00 |

| Table S5a  *Nationally Representative Descriptive Statistics for Germany (N = 9,506)* | |
| --- | --- |
| Characteristic | *n* (%) |
| **Relationship with mother when growing up** |  |
| Very good | 5,497 (58%) |
| Somewhat good | 3,031 (32%) |
| Somewhat bad | 496 (5.2%) |
| Very bad | 187 (2.0%) |
| Does not apply | 241 (2.5%) |
| Missing | 54 (0.6%) |
| **Relationship with father when growing up** |  |
| Very good | 4,652 (49%) |
| Somewhat good | 3,012 (32%) |
| Somewhat bad | 846 (8.9%) |
| Very bad | 385 (4.0%) |
| Does not apply | 538 (5.7%) |
| Missing | 73 (0.8%) |
| **Family structure around age 12** |  |
| Parents were married | 7,620 (80%) |
| Parents were divorced | 927 (9.8%) |
| Parents were never married | 578 (6.1%) |
| One or both parents had died | 245 (2.6%) |
| Missing | 136 (1.4%) |
| **Subjective financial status of family around age 12** |  |
| Lived comfortably | 3,177 (33%) |
| Got by | 4,508 (47%) |
| Found it difficult | 1,481 (16%) |
| Found it very difficult | 314 (3.3%) |
| Missing | 26 (0.3%) |
| **Experienced abuse when growing up** |  |
| Yes | 1,086 (11%) |
| No | 8,321 (88%) |
| Missing | 99 (1.0%) |
| **Felt like an outsider in the family when growing up** |  |
| Yes | 1,105 (12%) |
| No | 8,262 (87%) |
| Missing | 139 (1.5%) |
| **Self-rated health when growing up** |  |
| Excellent | 2,633 (28%) |
| Very good | 3,518 (37%) |
| Good | 2,582 (27%) |
| Fair | 612 (6.4%) |
| Poor | 134 (1.4%) |
| Missing | 26 (0.3%) |
| **Immigration status** |  |
| Born in this country | 8,722 (92%) |
| Born in another country | 744 (7.8%) |
| Missing | 40 (0.4%) |
| **Frequency of religious service attendance around age 12** |  |
| ≥ 1/week | 1,943 (20%) |
| 1-3/month | 1,899 (20%) |
| < 1/month | 2,887 (30%) |
| Never | 2,749 (29%) |
| Missing | 27 (0.3%) |
| **Year of birth** |  |
| 1998-2005 (current age: 18-24 years) | 829 (8.7%) |
| 1993-1998 (current age: 25-29 years) | 774 (8.1%) |
| 1983-1993 (current age: 30-39 years) | 1,438 (15%) |
| 1973-1983 (current age: 40-49 years) | 1,494 (16%) |
| 1963-1973 (current age: 50-59 years) | 1,729 (18%) |
| 1953-1963 (current age: 60-69 years) | 1,915 (20%) |
| 1943-1953 (current age: 70-79 years) | 1,137 (12%) |
| 1943 or earlier (current age: 80+ years) | 190 (2.0%) |
| Missing | 0 (0%) |
| **Gender** |  |
| Male | 4,641 (49%) |
| Female | 4,843 (51%) |
| Other | 11 (0.1%) |
| Missing | 11 (0.1%) |
| **Religious affiliation at age 12** |  |
| Baha’i | 2 (< 0.1%) |
| Buddhism | 25 (0.3%) |
| Christianity | 5,751 (61%) |
| Confucianism | 4 (< 0.1%) |
| Hinduism | 15 (0.2%) |
| Islam | 350 (3.7%) |
| Jainism | 1 (< 0.1%) |
| Judaism | 18 (0.2%) |
| No religion/atheist/agnostic | 3,163 (33%) |
| Primal, animist, or folk religion | 19 (0.2%) |
| Sikhism | 5 (< 0.1%) |
| Some other religion | 67 (0.7%) |
| Missing | 85 (0.9%) |
| **Racial/ethnic identity** |  |
| Missing | 9,506 (100%) |

| Table S5b  *Regression of Dispositional Forgivingness on Candidate Predictors for Germany* | | | | |
| --- | --- | --- | --- | --- |
| Variable | RR (95% CI) | Global  *p*-value | *E*-values | |
|  |  |  | *E*-value for estimate | *E*-value for 95% CI |
| **Relationship with mother when growing up** |  | < 0.001 |  |  |
| Ref: Very bad/somewhat bad | - |  | - | - |
| Very good/somewhat good | 1.12 (1.04, 1.19) |  | 1.46 | 1.24 |
| **Relationship with father when growing up** |  | 0.215 |  |  |
| Ref: Very bad/somewhat bad | - |  | - | - |
| Very good/somewhat good | 0.98 (0.94, 1.03) |  | 1.14 | 1.00 |
| **Family structure around age 12** |  | 0.013 |  |  |
| Ref: Parents were married | - |  | - | - |
| Parents were divorced | 0.97 (0.92, 1.03) |  | 1.19 | 1.00 |
| Parents were never married | 0.95 (0.89, 1.02) |  | 1.28 | 1.00 |
| One or both parents had died | 1.07 (0.99, 1.17) |  | 1.35 | 1.00 |
| **Subjective financial status of family around age 12** |  | 0.923 |  |  |
| Ref: Got by | - |  | - | - |
| Lived comfortably | 1.02 (0.98, 1.06) |  | 1.16 | 1.00 |
| Found it difficult | 1.00 (0.95, 1.05) |  | 1.02 | 1.00 |
| Found it very difficult | 0.97 (0.88, 1.06) |  | 1.21 | 1.00 |
| **Experienced abuse when growing up** |  | 0.106 |  |  |
| Ref: No | - |  | - | - |
| Yes | 1.03 (0.98, 1.08) |  | 1.21 | 1.00 |
| **Felt like an outsider in the family when growing up** |  | 0.976 |  |  |
| Ref: No | - |  | - | - |
| Yes | 0.99 (0.95, 1.04) |  | 1.09 | 1.00 |
| **Self-rated health when growing up** |  | < 0.001 |  |  |
| Ref: Good | - |  | - | - |
| Excellent | 1.04 (1.00, 1.09) |  | 1.25 | 1.00 |
| Very good | 1.03 (0.99, 1.07) |  | 1.21 | 1.00 |
| Fair | 1.03 (0.96, 1.10) |  | 1.20 | 1.00 |
| Poor | 1.19 (1.06, 1.32) |  | 1.65 | 1.32 |
| **Immigration status** |  | 0.179 |  |  |
| Ref: Born in this country | - |  | - | - |
| Born in another country | 0.96 (0.90, 1.03) |  | 1.24 | 1.00 |
| **Frequency of religious service attendance around age 12** |  | < 0.001 |  |  |
| Ref: Never | - |  | - | - |
| ≥ 1/week | 1.14 (1.08, 1.19) |  | 1.53 | 1.39 |
| 1-3/month | 1.14 (1.08, 1.19) |  | 1.52 | 1.38 |
| < 1/month | 1.06 (1.01, 1.11) |  | 1.31 | 1.14 |
| **Year of birth** |  | 0.006 |  |  |
| Ref: 1998-2005 (current age: 18-24 years) | - |  | - | - |
| 1993-1998 (current age: 25-29 years) | 1.00 (0.93, 1.08) |  | 1.03 | 1.00 |
| 1983-1993 (current age: 30-39 years) | 0.98 (0.91, 1.05) |  | 1.18 | 1.00 |
| 1973-1983 (current age: 40-49 years) | 0.99 (0.93, 1.06) |  | 1.10 | 1.00 |
| 1963-1973 (current age: 50-59 years) | 1.02 (0.95, 1.09) |  | 1.15 | 1.00 |
| 1953-1963 (current age: 60-69 years) | 0.99 (0.93, 1.07) |  | 1.08 | 1.00 |
| 1943-1953 (current age: 70-79 years) | 1.06 (0.99, 1.14) |  | 1.31 | 1.00 |
| 1943 or earlier (current age: 80+ years) | 1.07 (0.97, 1.20) |  | 1.37 | 1.00 |
| **Gender** |  | 0.107 |  |  |
| Ref: Male | - |  | - | - |
| Female | 1.03 (1.00, 1.06) |  | 1.19 | 1.00 |
| Other | 0.93 (0.53, 1.62) |  | 1.37 | 1.00 |
| **Religious affiliation at age 12** |  | < 0.001 |  |  |
| Ref: No religion/atheist/agnostic | - |  | - | - |
| Christianity | 1.05 (1.02, 1.10) |  | 1.30 | 1.15 |
| Some other religion | 1.12 (1.04, 1.19) |  | 1.47 | 1.24 |

| Table S6a  *Nationally Representative Descriptive Statistics for Hong Kong (Special Administrative Region of China; N = 3,012)* | |
| --- | --- |
| Characteristic | *n* (%) |
| **Relationship with mother when growing up** |  |
| Very good | 1,077 (36%) |
| Somewhat good | 1,164 (39%) |
| Somewhat bad | 293 (9.7%) |
| Very bad | 49 (1.6%) |
| Does not apply | 426 (14%) |
| Missing | 3 (< 0.1%) |
| **Relationship with father when growing up** |  |
| Very good | 868 (29%) |
| Somewhat good | 1,089 (36%) |
| Somewhat bad | 393 (13%) |
| Very bad | 102 (3.4%) |
| Does not apply | 557 (19%) |
| Missing | 3 (0.1%) |
| **Family structure around age 12** |  |
| Parents were married | 2,752 (91%) |
| Parents were divorced | 114 (3.8%) |
| Parents were never married | 40 (1.3%) |
| One or both parents had died | 50 (1.7%) |
| Missing | 56 (1.8%) |
| **Subjective financial status of family around age 12** |  |
| Lived comfortably | 906 (30%) |
| Got by | 1,527 (51%) |
| Found it difficult | 473 (16%) |
| Found it very difficult | 84 (2.8%) |
| Missing | 22 (0.7%) |
| **Experienced abuse when growing up** |  |
| Yes | 318 (11%) |
| No | 2,688 (89%) |
| Missing | 5 (0.2%) |
| **Felt like an outsider in the family when growing up** |  |
| Yes | 664 (22%) |
| No | 2,224 (74%) |
| Missing | 124 (4.1%) |
| **Self-rated health when growing up** |  |
| Excellent | 545 (18%) |
| Very good | 1,073 (36%) |
| Good | 863 (29%) |
| Fair | 426 (14%) |
| Poor | 91 (3.0%) |
| Missing | 13 (0.4%) |
| **Immigration status** |  |
| Born in this country | 2,637 (88%) |
| Born in another country | 321 (11%) |
| Missing | 53 (1.8%) |
| **Frequency of religious service attendance around age 12** |  |
| ≥ 1/week | 432 (14%) |
| 1-3/month | 528 (18%) |
| < 1/month | 753 (25%) |
| Never | 1,295 (43%) |
| Missing | 4 (0.1%) |
| **Year of birth** |  |
| 1998-2005 (current age: 18-24 years) | 217 (7.2%) |
| 1993-1998 (current age: 25-29 years) | 198 (6.6%) |
| 1983-1993 (current age: 30-39 years) | 507 (17%) |
| 1973-1983 (current age: 40-49 years) | 580 (19%) |
| 1963-1973 (current age: 50-59 years) | 711 (24%) |
| 1953-1963 (current age: 60-69 years) | 620 (21%) |
| 1943-1953 (current age: 70-79 years) | 164 (5.5%) |
| 1943 or earlier (current age: 80+ years) | 15 (0.5%) |
| Missing | 0 (0%) |
| **Gender** |  |
| Male | 1,390 (46%) |
| Female | 1,620 (54%) |
| Other | 2 (< 0.1%) |
| Missing | 0 (0%) |
| **Religious affiliation at age 12** |  |
| Buddhism | 323 (11%) |
| Chinese folk/traditional religion | 108 (3.6%) |
| Christianity | 715 (24%) |
| Confucianism | 10 (0.3%) |
| Hinduism | 27 (0.9%) |
| Islam | 86 (2.9%) |
| Jainism | 1 (< 0.1%) |
| Judaism | 16 (0.5%) |
| No religion/atheist/agnostic | 1,601 (53%) |
| Primal, animist, or folk religion | 15 (0.5%) |
| Shinto | 18 (0.6%) |
| Sikhism | 4 (0.1%) |
| Some other religion | 5 (0.2%) |
| Taoism | 81 (2.7%) |
| Missing | 1 (< 0.1%) |
| **Racial/ethnic identity** |  |
| Chinese (Cantonese) | 1,930 (64%) |
| Chinese (Chaoshan) | 201 (6.7%) |
| Chinese (Fujianese) | 117 (3.9%) |
| Chinese (Hakka) | 121 (4.0%) |
| Chinese (Other ethnicity) | 264 (8.8%) |
| Chinese (Shanghainese) | 89 (2.9%) |
| East Asian (Korean, Japanese) | 10 (0.3%) |
| Other | 4 (0.1%) |
| South Asian (Indian, Nepalese, Pakistani) | 17 (0.6%) |
| Southeast Asian (Filipino, Indonesian, Thailand) | 46 (1.5%) |
| Taiwanese | 14 (0.4%) |
| White | 15 (0.5%) |
| Missing | 184 (6.1%) |

| Table S6b  *Regression of Dispositional Forgivingness on Candidate Predictors for Hong Kong (Special Administrative Region of China)* | | | | | |
| --- | --- | --- | --- | --- | --- |
| Variable | RR (95% CI) | | Global  *p*-value | *E*-values | |
|  |  |  |  | *E*-value for estimate | *E*-value for estimate |
| **Relationship with mother when growing up** |  |  | 0.004 |  |  |
| Ref: Very bad/somewhat bad | - | |  | - | - |
| Very good/somewhat good | 1.06 | (0.94, 1.20) |  | 1.33 | 1.00 |
| **Relationship with father when growing up** |  |  | 0.831 |  |  |
| Ref: Very bad/somewhat bad | - | |  | - | - |
| Very good/somewhat good | 1.02 | (0.91, 1.15) |  | 1.16 | 1.00 |
| **Family structure around age 12** |  |  | 0.975 |  |  |
| Ref: Parents were married | - | |  | - | - |
| Parents were divorced | 0.97 | (0.79, 1.21) |  | 1.19 | 1.00 |
| Parents were never married | 0.80 | (0.53, 1.21) |  | 1.80 | 1.00 |
| One or both parents had died | 1.06 | (0.77, 1.45) |  | 1.31 | 1.00 |
| **Subjective financial status of family around age 12** |  |  | 0.597 |  |  |
| Ref: Got by | - | |  | - | - |
| Lived comfortably | 1.06 | (0.96, 1.16) |  | 1.31 | 1.00 |
| Found it difficult | 1.09 | (0.96, 1.24) |  | 1.41 | 1.00 |
| Found it very difficult | 1.06 | (0.80, 1.42) |  | 1.32 | 1.00 |
| **Experienced abuse when growing up** |  |  | 0.226 |  |  |
| Ref: No | - | |  | - | - |
| Yes | 0.93 | (0.81, 1.06) |  | 1.36 | 1.00 |
| **Felt like an outsider in the family when growing up** |  |  | < 0.001 |  |  |
| Ref: No | - | |  | - | - |
| Yes | 1.11 | (1.00, 1.22) |  | 1.44 | 1.00 |
| **Self-rated health when growing up** |  |  | < 0.001 |  |  |
| Ref: Good | - | |  | - | - |
| Excellent | 1.22 | (1.07, 1.39) |  | 1.74 | 1.35 |
| Very good | 1.14 | (1.02, 1.27) |  | 1.54 | 1.18 |
| Fair | 0.89 | (0.76, 1.04) |  | 1.50 | 1.00 |
| Poor | 1.00 | (0.74, 1.36) |  | 1.05 | 1.00 |
| **Immigration status** |  |  | 0.958 |  |  |
| Ref: Born in this country | - | |  | - | - |
| Born in another country | 0.97 | (0.82, 1.14) |  | 1.22 | 1.00 |
| **Frequency of religious service attendance around age 12** |  |  | < 0.001 |  |  |
| Ref: Never | - | |  | - | - |
| ≥ 1/week | 1.22 | (1.06, 1.41) |  | 1.75 | 1.32 |
| 1-3/month | 1.26 | (1.11, 1.42) |  | 1.83 | 1.46 |
| < 1/month | 1.13 | (1.01, 1.26) |  | 1.51 | 1.12 |
| **Year of birth** |  |  | < 0.001 |  |  |
| Ref: 1998-2005 (current age: 18-24 years) | - | |  | - | - |
| 1993-1998 (current age: 25-29 years) | 1.15 | (0.97, 1.35) |  | 1.55 | 1.00 |
| 1983-1993 (current age: 30-39 years) | 1.08 | (0.95, 1.24) |  | 1.39 | 1.00 |
| 1973-1983 (current age: 40-49 years) | 1.08 | (0.95, 1.23) |  | 1.38 | 1.00 |
| 1963-1973 (current age: 50-59 years) | 1.04 | (0.91, 1.19) |  | 1.25 | 1.00 |
| 1953-1963 (current age: 60-69 years) | 1.22 | (1.05, 1.43) |  | 1.75 | 1.28 |
| 1943-1953 (current age: 70-79 years) | 1.17 | (0.90, 1.53) |  | 1.62 | 1.00 |
| 1943 or earlier (current age: 80+ years) | 2.08 | (1.66, 2.60) |  | 3.57 | 2.70 |
| **Gender** |  |  | < 0.001 |  |  |
| Ref: Male | - | |  | - | - |
| Female | 0.97 | (0.90, 1.05) |  | 1.20 | 1.00 |
| Other | 0.00 | (0.00, 0.00) |  | * | * |
| **Religious affiliation at age 12** |  |  | 0.622 |  |  |
| Ref: No religion/atheist/agnostic | - | |  | - | - |
| Buddhism | 1.09 | (0.96, 1.24) |  | 1.41 | 1.00 |
| Chinese folk/traditional religion | 0.99 | (0.81, 1.21) |  | 1.12 | 1.00 |
| Christianity | 1.06 | (0.95, 1.19) |  | 1.32 | 1.00 |
| Some other religion | 0.95 | (0.80, 1.13) |  | 1.28 | 1.00 |
| **Racial/ethnic identity** |  |  | 0.091 |  |  |
| Ref: Majority | - | |  | - | - |
| Minority | 0.94 | (0.87, 1.03) |  | 1.30 | 1.00 |
| *Note*. *: *E*-values not estimable based on the observed data. | | | | | |

| Table S7a  *Nationally Representative Descriptive Statistics for India (N = 12,765)* | |
| --- | --- |
| Characteristic | *n* (%) |
| **Relationship with mother when growing up** |  |
| Very good | 11,465 (90%) |
| Somewhat good | 788 (6.2%) |
| Somewhat bad | 88 (0.7%) |
| Very bad | 73 (0.6%) |
| Does not apply | 269 (2.1%) |
| Missing | 82 (0.6%) |
| **Relationship with father when growing up** |  |
| Very good | 10,923 (86%) |
| Somewhat good | 995 (7.8%) |
| Somewhat bad | 126 (1.0%) |
| Very bad | 100 (0.8%) |
| Does not apply | 481 (3.8%) |
| Missing | 141 (1.1%) |
| **Family structure around age 12** |  |
| Parents were married | 5,578 (44%) |
| Parents were divorced | 236 (1.8%) |
| Parents were never married | 1,055 (8.3%) |
| One or both parents had died | 940 (7.4%) |
| Missing | 4,956 (39%) |
| **Subjective financial status of family around age 12** |  |
| Lived comfortably | 4,946 (39%) |
| Got by | 3,010 (24%) |
| Found it difficult | 2,703 (21%) |
| Found it very difficult | 2,035 (16%) |
| Missing | 70 (0.5%) |
| **Experienced abuse when growing up** |  |
| Yes | 1,468 (11%) |
| No | 10,526 (82%) |
| Missing | 771 (6.0%) |
| **Felt like an outsider in the family when growing up** |  |
| Yes | 1,926 (15%) |
| No | 10,780 (84%) |
| Missing | 59 (0.5%) |
| **Self-rated health when growing up** |  |
| Excellent | 2,182 (17%) |
| Very good | 3,882 (30%) |
| Good | 4,028 (32%) |
| Fair | 2,202 (17%) |
| Poor | 424 (3.3%) |
| Missing | 47 (0.4%) |
| **Immigration status** |  |
| Born in this country | 12,629 (99%) |
| Born in another country | 110 (0.9%) |
| Missing | 26 (0.2%) |
| **Frequency of religious service attendance around age 12** |  |
| ≥ 1/week | 5,288 (41%) |
| 1-3/month | 2,959 (23%) |
| < 1/month | 2,719 (21%) |
| Never | 1,478 (12%) |
| Missing | 321 (2.5%) |
| **Year of birth** |  |
| 1998-2005 (current age: 18-24 years) | 2,543 (20%) |
| 1993-1998 (current age: 25-29 years) | 1,640 (13%) |
| 1983-1993 (current age: 30-39 years) | 3,109 (24%) |
| 1973-1983 (current age: 40-49 years) | 2,275 (18%) |
| 1963-1973 (current age: 50-59 years) | 1,574 (12%) |
| 1953-1963 (current age: 60-69 years) | 1,188 (9.3%) |
| 1943-1953 (current age: 70-79 years) | 370 (2.9%) |
| 1943 or earlier (current age: 80+ years) | 67 (0.5%) |
| Missing | 0 (0%) |
| **Gender** |  |
| Male | 6,473 (51%) |
| Female | 6,292 (49%) |
| Other | 0 (0%) |
| Missing | 0 (0%) |
| **Religious affiliation at age 12** |  |
| Buddhism | 180 (1.4%) |
| Christianity | 254 (2.0%) |
| Hinduism | 10,417 (82%) |
| Islam | 1,550 (12%) |
| Jainism | 9 (< 0.1%) |
| No religion/atheist/agnostic | 7 (< 0.1%) |
| Primal, animist, or folk religion | 27 (0.2%) |
| Shinto | 4 (< 0.1%) |
| Sikhism | 126 (1.0%) |
| Some other religion | 59 (0.5%) |
| Missing | 131 (1.0%) |
| **Racial/ethnic identity** |  |
| General | 3,538 (28%) |
| Other backward caste | 4,177 (33%) |
| Schedule caste | 3,599 (28%) |
| Schedule tribe | 1,185 (9.3%) |
| Missing | 267 (2.1%) |

| Table S7b  *Regression of Dispositional Forgivingness on Candidate Predictors for India* | | | | |
| --- | --- | --- | --- | --- |
| Variable | RR (95% CI) | Global  *p*-value | *E*-values | |
|  |  |  | *E*-value for estimate | *E*-value for 95% CI |
| **Relationship with mother when growing up** |  | 0.386 |  |  |
| Ref: Very bad/somewhat bad | - |  | - | - |
| Very good/somewhat good | 0.97 (0.88, 1.07) |  | 1.19 | 1.00 |
| **Relationship with father when growing up** |  | < 0.001 |  |  |
| Ref: Very bad/somewhat bad | - |  | - | - |
| Very good/somewhat good | 1.06 (0.98, 1.17) |  | 1.33 | 1.00 |
| **Family structure around age 12** |  | 0.009 |  |  |
| Ref: Parents were married | - |  | - | - |
| Parents were divorced | 0.98 (0.85, 1.14) |  | 1.14 | 1.00 |
| Parents were never married | 1.04 (0.99, 1.09) |  | 1.23 | 1.00 |
| One or both parents had died | 1.01 (0.95, 1.06) |  | 1.09 | 1.00 |
| **Subjective financial status of family around age 12** |  | 1.000 |  |  |
| Ref: Got by | - |  | - | - |
| Lived comfortably | 0.99 (0.96, 1.02) |  | 1.10 | 1.00 |
| Found it difficult | 1.00 (0.96, 1.04) |  | 1.06 | 1.00 |
| Found it very difficult | 1.00 (0.95, 1.04) |  | 1.07 | 1.00 |
| **Experienced abuse when growing up** |  | < 0.001 |  |  |
| Ref: No | - |  | - | - |
| Yes | 0.94 (0.90, 0.98) |  | 1.33 | 1.17 |
| **Felt like an outsider in the family when growing up** |  | < 0.001 |  |  |
| Ref: No | - |  | - | - |
| Yes | 1.03 (1.00, 1.06) |  | 1.22 | 1.06 |
| **Self-rated health when growing up** |  | 0.608 |  |  |
| Ref: Good | - |  | - | - |
| Excellent | 1.00 (0.96, 1.04) |  | 1.02 | 1.00 |
| Very good | 1.00 (0.97, 1.04) |  | 1.05 | 1.00 |
| Fair | 0.97 (0.94, 1.01) |  | 1.21 | 1.00 |
| Poor | 0.98 (0.91, 1.06) |  | 1.15 | 1.00 |
| **Immigration status** |  | 0.991 |  |  |
| Ref: Born in this country | - |  | - | - |
| Born in another country | 1.02 (0.89, 1.17) |  | 1.16 | 1.00 |
| **Frequency of religious service attendance around age 12** |  | < 0.001 |  |  |
| Ref: Never | - |  | - | - |
| ≥ 1/week | 1.04 (1.00, 1.09) |  | 1.25 | 1.00 |
| 1-3/month | 1.01 (0.96, 1.06) |  | 1.11 | 1.00 |
| < 1/month | 1.00 (0.95, 1.05) |  | 1.06 | 1.00 |
| **Year of birth** |  | 0.093 |  |  |
| Ref: 1998-2005 (current age: 18-24 years) | - |  | - | - |
| 1993-1998 (current age: 25-29 years) | 1.05 (1.00, 1.09) |  | 1.27 | 1.07 |
| 1983-1993 (current age: 30-39 years) | 1.04 (1.00, 1.08) |  | 1.23 | 1.00 |
| 1973-1983 (current age: 40-49 years) | 1.04 (1.00, 1.09) |  | 1.26 | 1.06 |
| 1963-1973 (current age: 50-59 years) | 1.03 (0.98, 1.08) |  | 1.19 | 1.00 |
| 1953-1963 (current age: 60-69 years) | 1.07 (1.01, 1.13) |  | 1.34 | 1.13 |
| 1943-1953 (current age: 70-79 years) | 1.06 (0.98, 1.15) |  | 1.32 | 1.00 |
| 1943 or earlier (current age: 80+ years) | 1.14 (0.97, 1.33) |  | 1.53 | 1.00 |
| **Gender** |  | 0.001 |  |  |
| Ref: Male | - |  | - | - |
| Female | 1.02 (1.00, 1.05) |  | 1.18 | 1.01 |
| **Religious affiliation at age 12** |  | < 0.001 |  |  |
| Ref: Hinduism | - |  | - | - |
| Islam | 1.07 (1.03, 1.12) |  | 1.35 | 1.21 |
| Some other religion | 1.01 (0.96, 1.07) |  | 1.13 | 1.00 |
| **Racial/ethnic identity** |  | < 0.001 |  |  |
| Ref: Majority | - |  | - | - |
| Minority | 1.03 (1.00, 1.06) |  | 1.22 | 1.07 |

| Table S8a  *Nationally Representative Descriptive Statistics for Indonesia (N = 6,992)* | |
| --- | --- |
| Characteristic | *n* (%) |
| **Relationship with mother when growing up** |  |
| Very good | 6,238 (89%) |
| Somewhat good | 583 (8.3%) |
| Somewhat bad | 50 (0.7%) |
| Very bad | 26 (0.4%) |
| Does not apply | 68 (1.0%) |
| Missing | 27 (0.4%) |
| **Relationship with father when growing up** |  |
| Very good | 6,067 (87%) |
| Somewhat good | 628 (9.0%) |
| Somewhat bad | 68 (1.0%) |
| Very bad | 52 (0.7%) |
| Does not apply | 115 (1.6%) |
| Missing | 61 (0.9%) |
| **Family structure around age 12** |  |
| Parents were married | 5,557 (79%) |
| Parents were divorced | 448 (6.4%) |
| Parents were never married | 47 (0.7%) |
| One or both parents had died | 735 (11%) |
| Missing | 205 (2.9%) |
| **Subjective financial status of family around age 12** |  |
| Lived comfortably | 3,408 (49%) |
| Got by | 2,955 (42%) |
| Found it difficult | 439 (6.3%) |
| Found it very difficult | 181 (2.6%) |
| Missing | 9 (0.1%) |
| **Experienced abuse when growing up** |  |
| Yes | 486 (6.9%) |
| No | 6,427 (92%) |
| Missing | 79 (1.1%) |
| **Felt like an outsider in the family when growing up** |  |
| Yes | 343 (4.9%) |
| No | 6,639 (95%) |
| Missing | 10 (0.1%) |
| **Self-rated health when growing up** |  |
| Excellent | 1,246 (18%) |
| Very good | 1,968 (28%) |
| Good | 2,490 (36%) |
| Fair | 1,233 (18%) |
| Poor | 55 (0.8%) |
| Missing | 1 (< 0.1%) |
| **Immigration status** |  |
| Born in this country | 6,958 (100%) |
| Born in another country | 34 (0.5%) |
| Missing | 0 (0%) |
| **Frequency of religious service attendance around age 12** |  |
| ≥ 1/week | 5,363 (77%) |
| 1-3/month | 973 (14%) |
| < 1/month | 329 (4.7%) |
| Never | 275 (3.9%) |
| Missing | 51 (0.7%) |
| **Year of birth** |  |
| 1998-2005 (current age: 18-24 years) | 1,216 (17%) |
| 1993-1998 (current age: 25-29 years) | 849 (12%) |
| 1983-1993 (current age: 30-39 years) | 1,591 (23%) |
| 1973-1983 (current age: 40-49 years) | 1,576 (23%) |
| 1963-1973 (current age: 50-59 years) | 1,169 (17%) |
| 1953-1963 (current age: 60-69 years) | 490 (7.0%) |
| 1943-1953 (current age: 70-79 years) | 83 (1.2%) |
| 1943 or earlier (current age: 80+ years) | 17 (0.2%) |
| Missing | 0 (0%) |
| **Gender** |  |
| Male | 3,461 (50%) |
| Female | 3,513 (50%) |
| Other | 7 (< 0.1%) |
| Missing | 11 (0.2%) |
| **Religious affiliation at age 12** |  |
| Buddhism | 5 (< 0.1%) |
| Christianity | 528 (7.6%) |
| Confucianism | 1 (< 0.1%) |
| Hinduism | 75 (1.1%) |
| Islam | 6,373 (91%) |
| Jainism | 1 (< 0.1%) |
| No religion/atheist/agnostic | 2 (< 0.1%) |
| Primal, animist, or folk religion | 1 (< 0.1%) |
| Taoism | 0 (< 0.1%) |
| Missing | 8 (0.1%) |
| **Racial/ethnic identity** |  |
| Bali | 69 (1.0%) |
| Banjar/Melayu Banjar | 320 (4.6%) |
| Batak | 165 (2.4%) |
| Betawi | 251 (3.6%) |
| Bugis | 243 (3.5%) |
| Jawa | 2,846 (41%) |
| Madura | 262 (3.7%) |
| Makasar | 91 (1.3%) |
| Minangkabau | 273 (3.9%) |
| Other | 1,262 (18%) |
| Sunda/Parahyangan | 1,172 (17%) |
| Missing | 38 (0.5%) |

| Table S8b  *Regression of Dispositional Forgivingness on Candidate Predictors for Indonesia* | | | | |
| --- | --- | --- | --- | --- |
| Variable | RR (95% CI) | Global  *p*-value | *E*-values | |
|  |  |  | *E*-value for estimate | *E*-value for 95% CI |
| **Relationship with mother when growing up** |  | 0.760 |  |  |
| Ref: Very bad/somewhat bad | - |  | - | - |
| Very good/somewhat good | 1.03 (0.88, 1.19) |  | 1.19 | 1.00 |
| **Relationship with father when growing up** |  | 0.945 |  |  |
| Ref: Very bad/somewhat bad | - |  | - | - |
| Very good/somewhat good | 0.99 (0.91, 1.07) |  | 1.11 | 1.00 |
| **Family structure around age 12** |  | 0.095 |  |  |
| Ref: Parents were married | - |  | - | - |
| Parents were divorced | 1.00 (0.95, 1.06) |  | 1.07 | 1.00 |
| Parents were never married | 0.91 (0.77, 1.08) |  | 1.43 | 1.00 |
| One or both parents had died | 0.95 (0.91, 1.00) |  | 1.27 | 1.00 |
| **Subjective financial status of family around age 12** |  | 0.293 |  |  |
| Ref: Got by | - |  | - | - |
| Lived comfortably | 1.01 (0.98, 1.04) |  | 1.11 | 1.00 |
| Found it difficult | 0.98 (0.91, 1.04) |  | 1.18 | 1.00 |
| Found it very difficult | 0.91 (0.81, 1.03) |  | 1.42 | 1.00 |
| **Experienced abuse when growing up** |  | 0.015 |  |  |
| Ref: No | - |  | - | - |
| Yes | 0.95 (0.90, 1.01) |  | 1.27 | 1.00 |
| **Felt like an outsider in the family when growing up** |  | 0.058 |  |  |
| Ref: No | - |  | - | - |
| Yes | 1.04 (0.98, 1.10) |  | 1.24 | 1.00 |
| **Self-rated health when growing up** |  | 0.483 |  |  |
| Ref: Good | - |  | - | - |
| Excellent | 1.03 (0.99, 1.07) |  | 1.22 | 1.00 |
| Very good | 1.02 (0.99, 1.05) |  | 1.17 | 1.00 |
| Fair | 0.99 (0.96, 1.03) |  | 1.08 | 1.00 |
| Poor | 1.01 (0.84, 1.22) |  | 1.13 | 1.00 |
| **Immigration status** |  | 0.886 |  |  |
| Ref: Born in this country | - |  | - | - |
| Born in another country | 1.03 (0.92, 1.16) |  | 1.21 | 1.00 |
| **Frequency of religious service attendance around age 12** |  | 0.197 |  |  |
| Ref: Never | - |  | - | - |
| ≥ 1/week | 1.08 (0.98, 1.19) |  | 1.38 | 1.00 |
| 1-3/month | 1.08 (0.98, 1.19) |  | 1.37 | 1.00 |
| < 1/month | 1.12 (1.00, 1.24) |  | 1.47 | 1.00 |
| **Year of birth** |  | < 0.001 |  |  |
| Ref: 1998-2005 (current age: 18-24 years) | - |  | - | - |
| 1993-1998 (current age: 25-29 years) | 1.02 (0.98, 1.06) |  | 1.16 | 1.00 |
| 1983-1993 (current age: 30-39 years) | 1.02 (0.99, 1.06) |  | 1.18 | 1.00 |
| 1973-1983 (current age: 40-49 years) | 1.07 (1.02, 1.11) |  | 1.34 | 1.18 |
| 1963-1973 (current age: 50-59 years) | 1.03 (0.98, 1.08) |  | 1.20 | 1.00 |
| 1953-1963 (current age: 60-69 years) | 1.07 (1.02, 1.14) |  | 1.36 | 1.16 |
| 1943-1953 (current age: 70-79 years) | 0.97 (0.81, 1.16) |  | 1.22 | 1.00 |
| 1943 or earlier (current age: 80+ years) | 1.23 (1.11, 1.38) |  | 1.78 | 1.46 |
| **Gender** |  | 0.002 |  |  |
| Ref: Male | - |  | - | - |
| Female | 1.02 (1.00, 1.05) |  | 1.18 | 1.01 |
| Other | 0.48 (0.15, 1.56) |  | 3.60 | 1.00 |
| **Religious affiliation at age 12** |  | 0.638 |  |  |
| Ref: Islam | - |  | - | - |
| Christianity | 1.01 (0.97, 1.06) |  | 1.13 | 1.00 |
| Some other religion | 0.90 (0.76, 1.08) |  | 1.44 | 1.00 |
| **Racial/ethnic identity** |  | 0.118 |  |  |
| Ref: Majority | - |  | - | - |
| Minority | 0.98 (0.96, 1.01) |  | 1.15 | 1.00 |

| Table S9a  *Nationally Representative Descriptive Statistics for Israel (N = 3,669)* | |
| --- | --- |
| Characteristic | *n* (%) |
| **Relationship with mother when growing up** |  |
| Very good | 2,686 (73%) |
| Somewhat good | 793 (22%) |
| Somewhat bad | 110 (3.0%) |
| Very bad | 18 (0.5%) |
| Does not apply | 45 (1.2%) |
| Missing | 17 (0.5%) |
| **Relationship with father when growing up** |  |
| Very good | 2,290 (62%) |
| Somewhat good | 912 (25%) |
| Somewhat bad | 234 (6.4%) |
| Very bad | 37 (1.0%) |
| Does not apply | 171 (4.7%) |
| Missing | 25 (0.7%) |
| **Family structure around age 12** |  |
| Parents were married | 3,172 (86%) |
| Parents were divorced | 284 (7.8%) |
| Parents were never married | 36 (1.0%) |
| One or both parents had died | 130 (3.5%) |
| Missing | 47 (1.3%) |
| **Subjective financial status of family around age 12** |  |
| Lived comfortably | 923 (25%) |
| Got by | 1,822 (50%) |
| Found it difficult | 667 (18%) |
| Found it very difficult | 239 (6.5%) |
| Missing | 17 (0.5%) |
| **Experienced abuse when growing up** |  |
| Yes | 0 (0%) |
| No | 0 (0%) |
| Missing | 3,669 (100%) |
| **Felt like an outsider in the family when growing up** |  |
| Yes | 371 (10%) |
| No | 3,228 (88%) |
| Missing | 70 (1.9%) |
| **Self-rated health when growing up** |  |
| Excellent | 1,785 (49%) |
| Very good | 1,284 (35%) |
| Good | 480 (13%) |
| Fair | 105 (2.9%) |
| Poor | 6 (0.2%) |
| Missing | 8 (0.2%) |
| **Immigration status** |  |
| Born in this country | 2,796 (76%) |
| Born in another country | 868 (24%) |
| Missing | 5 (0.1%) |
| **Frequency of religious service attendance around age 12** |  |
| ≥ 1/week | 867 (24%) |
| 1-3/month | 435 (12%) |
| < 1/month | 810 (22%) |
| Never | 1,539 (42%) |
| Missing | 17 (0.5%) |
| **Year of birth** |  |
| 1998-2005 (current age: 18-24 years) | 553 (15%) |
| 1993-1998 (current age: 25-29 years) | 407 (11%) |
| 1983-1993 (current age: 30-39 years) | 666 (18%) |
| 1973-1983 (current age: 40-49 years) | 616 (17%) |
| 1963-1973 (current age: 50-59 years) | 542 (15%) |
| 1953-1963 (current age: 60-69 years) | 469 (13%) |
| 1943-1953 (current age: 70-79 years) | 336 (9.2%) |
| 1943 or earlier (current age: 80+ years) | 79 (2.2%) |
| Missing | 0 (0%) |
| **Gender** |  |
| Male | 1,791 (49%) |
| Female | 1,872 (51%) |
| Other | 0 (< 0.1%) |
| Missing | 6 (0.2%) |
| **Religious affiliation at age 12** |  |
| Baha’i | 1 (< 0.1%) |
| Christianity | 60 (1.6%) |
| Islam | 647 (18%) |
| Judaism | 2,873 (78%) |
| No religion/atheist/agnostic | 69 (1.9%) |
| Primal, animist, or folk religion | 3 (< 0.1%) |
| Sikhism | 1 (< 0.1%) |
| Some other religion | 5 (0.1%) |
| Missing | 10 (0.3%) |
| **Racial/ethnic identity** |  |
| Arab | 674 (18%) |
| Jewish | 2,926 (80%) |
| Other | 39 (1.1%) |
| Missing | 30 (0.8%) |

| Table S9b  *Regression of Dispositional Forgivingness on Candidate Predictors for Israel* | | | | |
| --- | --- | --- | --- | --- |
| Variable | RR (95% CI) | Global  *p*-value | *E*-values | |
|  |  |  | *E*-value for estimate | *E*-value for estimate |
| **Relationship with mother when growing up** |  | 0.316 |  |  |
| Ref: Very bad/somewhat bad | - |  | - | - |
| Very good/somewhat good | 0.96 (0.85, 1.09) |  | 1.23 | 1.00 |
| **Relationship with father when growing up** |  | 0.007 |  |  |
| Ref: Very bad/somewhat bad | - |  | - | - |
| Very good/somewhat good | 1.06 (0.95, 1.17) |  | 1.30 | 1.00 |
| **Family structure around age 12** |  | 0.037 |  |  |
| Ref: Parents were married | - |  | - | - |
| Parents were divorced | 0.88 (0.78, 0.98) |  | 1.54 | 1.14 |
| Parents were never married | 1.03 (0.82, 1.29) |  | 1.19 | 1.00 |
| One or both parents had died | 1.01 (0.93, 1.10) |  | 1.13 | 1.00 |
| **Subjective financial status of family around age 12** |  | 0.987 |  |  |
| Ref: Got by | - |  | - | - |
| Lived comfortably | 1.00 (0.95, 1.05) |  | 1.06 | 1.00 |
| Found it difficult | 1.02 (0.95, 1.08) |  | 1.15 | 1.00 |
| Found it very difficult | 0.95 (0.84, 1.08) |  | 1.29 | 1.00 |
| **Felt like an outsider in the family when growing up** |  | 0.806 |  |  |
| Ref: No | - |  | - | - |
| Yes | 0.97 (0.89, 1.07) |  | 1.20 | 1.00 |
| **Self-rated health when growing up** |  | < 0.001 |  |  |
| Ref: Good | - |  | - | - |
| Excellent | 1.00 (0.92, 1.09) |  | 1.06 | 1.00 |
| Very good | 1.01 (0.93, 1.09) |  | 1.10 | 1.00 |
| Fair | 0.87 (0.71, 1.07) |  | 1.56 | 1.00 |
| Poor | 1.57 (1.25, 1.96) |  | 2.50 | 1.81 |
| **Immigration status** |  | 0.017 |  |  |
| Ref: Born in this country | - |  | - | - |
| Born in another country | 0.95 (0.89, 1.01) |  | 1.29 | 1.00 |
| **Frequency of religious service attendance around age 12** |  | < 0.001 |  |  |
| Ref: Never | - |  | - | - |
| ≥ 1/week | 1.15 (1.07, 1.24) |  | 1.56 | 1.34 |
| 1-3/month | 1.22 (1.15, 1.31) |  | 1.76 | 1.57 |
| < 1/month | 1.17 (1.11, 1.25) |  | 1.63 | 1.45 |
| **Year of birth** |  | 0.783 |  |  |
| Ref: 1998-2005 (current age: 18-24 years) | - |  | - | - |
| 1993-1998 (current age: 25-29 years) | 1.01 (0.91, 1.11) |  | 1.10 | 1.00 |
| 1983-1993 (current age: 30-39 years) | 1.01 (0.93, 1.10) |  | 1.12 | 1.00 |
| 1973-1983 (current age: 40-49 years) | 1.06 (0.98, 1.15) |  | 1.32 | 1.00 |
| 1963-1973 (current age: 50-59 years) | 1.05 (0.96, 1.14) |  | 1.28 | 1.00 |
| 1953-1963 (current age: 60-69 years) | 1.08 (0.99, 1.18) |  | 1.38 | 1.00 |
| 1943-1953 (current age: 70-79 years) | 1.09 (0.97, 1.24) |  | 1.43 | 1.00 |
| 1943 or earlier (current age: 80+ years) | 1.08 (0.91, 1.29) |  | 1.37 | 1.00 |
| **Gender** |  | < 0.001 |  |  |
| Ref: Male | - |  | - | - |
| Female | 1.08 (1.03, 1.13) |  | 1.37 | 1.21 |
| Other | 0.00 (0.00, 0.00) |  | * | * |
| **Religious affiliation at age 12** |  | 0.001 |  |  |
| Ref: Judaism | - |  | - | - |
| Islam | 0.75 (0.59, 0.96) |  | 2.00 | 1.25 |
| Some other religion | 0.79 (0.64, 0.98) |  | 1.83 | 1.17 |
| **Racial/ethnic identity** |  | 0.409 |  |  |
| Ref: Majority | - |  | - | - |
| Minority | 1.11 (0.89, 1.38) |  | 1.46 | 1.00 |
| *Note*. *: *E*-values not estimable based on the observed data. | | | | |

| Table S10a  *Nationally Representative Descriptive Statistics for Japan (N = 20,543)* | |
| --- | --- |
| Characteristic | *n* (%) |
| **Relationship with mother when growing up** |  |
| Very good | 5,630 (27%) |
| Somewhat good | 9,461 (46%) |
| Somewhat bad | 2,750 (13%) |
| Very bad | 799 (3.9%) |
| Does not apply | 1,838 (8.9%) |
| Missing | 66 (0.3%) |
| **Relationship with father when growing up** |  |
| Very good | 4,156 (20%) |
| Somewhat good | 9,081 (44%) |
| Somewhat bad | 3,446 (17%) |
| Very bad | 1,223 (6.0%) |
| Does not apply | 2,580 (13%) |
| Missing | 57 (0.3%) |
| **Family structure around age 12** |  |
| Parents were married | 17,713 (86%) |
| Parents were divorced | 1,127 (5.5%) |
| Parents were never married | 591 (2.9%) |
| One or both parents had died | 754 (3.7%) |
| Missing | 359 (1.7%) |
| **Subjective financial status of family around age 12** |  |
| Lived comfortably | 8,320 (41%) |
| Got by | 8,799 (43%) |
| Found it difficult | 2,398 (12%) |
| Found it very difficult | 973 (4.7%) |
| Missing | 52 (0.3%) |
| **Experienced abuse when growing up** |  |
| Yes | 1,482 (7.2%) |
| No | 18,964 (92%) |
| Missing | 96 (0.5%) |
| **Felt like an outsider in the family when growing up** |  |
| Yes | 1,963 (9.6%) |
| No | 17,136 (83%) |
| Missing | 1,444 (7.0%) |
| **Self-rated health when growing up** |  |
| Excellent | 2,711 (13%) |
| Very good | 7,106 (35%) |
| Good | 6,689 (33%) |
| Fair | 3,199 (16%) |
| Poor | 758 (3.7%) |
| Missing | 80 (0.4%) |
| **Immigration status** |  |
| Born in this country | 19,548 (95%) |
| Born in another country | 158 (0.8%) |
| Missing | 837 (4.1%) |
| **Frequency of religious service attendance around age 12** |  |
| ≥ 1/week | 398 (1.9%) |
| 1-3/month | 883 (4.3%) |
| < 1/month | 5,023 (24%) |
| Never | 14,117 (69%) |
| Missing | 123 (0.6%) |
| **Year of birth** |  |
| 1998-2005 (current age: 18-24 years) | 1,589 (7.7%) |
| 1993-1998 (current age: 25-29 years) | 806 (3.9%) |
| 1983-1993 (current age: 30-39 years) | 2,851 (14%) |
| 1973-1983 (current age: 40-49 years) | 3,363 (16%) |
| 1963-1973 (current age: 50-59 years) | 3,770 (18%) |
| 1953-1963 (current age: 60-69 years) | 4,118 (20%) |
| 1943-1953 (current age: 70-79 years) | 3,554 (17%) |
| 1943 or earlier (current age: 80+ years) | 493 (2.4%) |
| Missing | 0 (0%) |
| **Gender** |  |
| Male | 9,847 (48%) |
| Female | 10,602 (52%) |
| Other | 28 (0.1%) |
| Missing | 66 (0.3%) |
| **Religious affiliation at age 12** |  |
| Baha’i | 7 (< 0.1%) |
| Buddhism | 6,536 (32%) |
| Christianity | 343 (1.7%) |
| Confucianism | 25 (0.1%) |
| Hinduism | 4 (< 0.1%) |
| Islam | 7 (< 0.1%) |
| Jainism | 1 (< 0.1%) |
| No religion/atheist/agnostic | 12,950 (63%) |
| Primal, animist, or folk religion | 13 (< 0.1%) |
| Shinto | 382 (1.9%) |
| Some other religion | 46 (0.2%) |
| Taoism | 14 (< 0.1%) |
| Missing | 215 (1.0%) |
| **Racial/ethnic identity** |  |
| Missing | 20,543 (100%) |

| Table S10b  *Regression of Dispositional Forgivingness on Candidate Predictors for Japan* | | | | | |
| --- | --- | --- | --- | --- | --- |
| Variable | RR (95% CI) | | Global  *p*-value | *E*-values | |
|  |  |  |  | *E*-value for estimate | *E*-value for estimate |
| **Relationship with mother when growing up** |  |  | < 0.001 |  |  |
| Ref: Very bad/somewhat bad | - | |  | - | - |
| Very good/somewhat good | 1.12 | (1.06, 1.17) |  | 1.47 | 1.32 |
| **Relationship with father when growing up** |  |  | < 0.001 |  |  |
| Ref: Very bad/somewhat bad | - | |  | - | - |
| Very good/somewhat good | 1.13 | (1.09, 1.18) |  | 1.52 | 1.39 |
| **Family structure around age 12** |  |  | 0.075 |  |  |
| Ref: Parents were married | - | |  | - | - |
| Parents were divorced | 1.07 | (0.99, 1.15) |  | 1.34 | 1.00 |
| Parents were never married | 0.96 | (0.88, 1.05) |  | 1.23 | 1.00 |
| One or both parents had died | 1.00 | (0.91, 1.09) |  | 1.06 | 1.00 |
| **Subjective financial status of family around age 12** |  |  | < 0.001 |  |  |
| Ref: Got by | - | |  | - | - |
| Lived comfortably | 1.06 | (1.03, 1.09) |  | 1.31 | 1.19 |
| Found it difficult | 0.93 | (0.89, 0.99) |  | 1.34 | 1.14 |
| Found it very difficult | 0.97 | (0.89, 1.05) |  | 1.22 | 1.00 |
| **Experienced abuse when growing up** |  |  | 0.012 |  |  |
| Ref: No | - | |  | - | - |
| Yes | 1.05 | (0.99, 1.12) |  | 1.29 | 1.00 |
| **Felt like an outsider in the family when growing up** |  |  | 0.498 |  |  |
| Ref: No | - | |  | - | - |
| Yes | 0.98 | (0.92, 1.03) |  | 1.18 | 1.00 |
| **Self-rated health when growing up** |  |  | < 0.001 |  |  |
| Ref: Good | - | |  | - | - |
| Excellent | 1.11 | (1.06, 1.15) |  | 1.44 | 1.30 |
| Very good | 1.08 | (1.05, 1.12) |  | 1.39 | 1.28 |
| Fair | 0.90 | (0.86, 0.95) |  | 1.46 | 1.30 |
| Poor | 0.90 | (0.83, 1.00) |  | 1.44 | 1.07 |
| **Immigration status** |  |  | 0.425 |  |  |
| Ref: Born in this country | - | |  | - | - |
| Born in another country | 0.92 | (0.77, 1.10) |  | 1.39 | 1.00 |
| **Frequency of religious service attendance around age 12** |  |  | < 0.001 |  |  |
| Ref: Never | - | |  | - | - |
| ≥ 1/week | 1.19 | (1.09, 1.29) |  | 1.65 | 1.41 |
| 1-3/month | 1.20 | (1.13, 1.28) |  | 1.69 | 1.51 |
| < 1/month | 1.11 | (1.07, 1.14) |  | 1.46 | 1.36 |
| **Year of birth** |  |  | < 0.001 |  |  |
| Ref: 1998-2005 (current age: 18-24 years) | - | |  | - | - |
| 1993-1998 (current age: 25-29 years) | 0.90 | (0.82, 0.99) |  | 1.46 | 1.14 |
| 1983-1993 (current age: 30-39 years) | 0.93 | (0.88, 1.00) |  | 1.34 | 1.04 |
| 1973-1983 (current age: 40-49 years) | 0.93 | (0.87, 0.99) |  | 1.35 | 1.09 |
| 1963-1973 (current age: 50-59 years) | 0.96 | (0.91, 1.02) |  | 1.23 | 1.00 |
| 1953-1963 (current age: 60-69 years) | 1.03 | (0.97, 1.09) |  | 1.22 | 1.00 |
| 1943-1953 (current age: 70-79 years) | 1.07 | (1.02, 1.14) |  | 1.36 | 1.14 |
| 1943 or earlier (current age: 80+ years) | 1.16 | (1.06, 1.27) |  | 1.59 | 1.32 |
| **Gender** |  |  | < 0.001 |  |  |
| Ref: Male | - | |  | - | - |
| Female | 1.08 | (1.06, 1.12) |  | 1.40 | 1.31 |
| Other | 1.17 | (0.86, 1.58) |  | 1.61 | 1.00 |
| **Religious affiliation at age 12** |  |  | < 0.001 |  |  |
| Ref: No religion/atheist/agnostic | - | |  | - | - |
| Buddhism | 1.04 | (1.01, 1.07) |  | 1.25 | 1.11 |
| Some other religion | 1.04 | (0.98, 1.12) |  | 1.26 | 1.00 |

| Table S11a  *Nationally Representative Descriptive Statistics for Kenya (N = 11,389)* | |
| --- | --- |
| Characteristic | *n* (%) |
| **Relationship with mother when growing up** |  |
| Very good | 9,418 (83%) |
| Somewhat good | 1,435 (13%) |
| Somewhat bad | 130 (1.1%) |
| Very bad | 100 (0.9%) |
| Does not apply | 240 (2.1%) |
| Missing | 66 (0.6%) |
| **Relationship with father when growing up** |  |
| Very good | 7,958 (70%) |
| Somewhat good | 1,896 (17%) |
| Somewhat bad | 216 (1.9%) |
| Very bad | 220 (1.9%) |
| Does not apply | 967 (8.5%) |
| Missing | 132 (1.2%) |
| **Family structure around age 12** |  |
| Parents were married | 9,238 (81%) |
| Parents were divorced | 697 (6.1%) |
| Parents were never married | 681 (6.0%) |
| One or both parents had died | 471 (4.1%) |
| Missing | 301 (2.6%) |
| **Subjective financial status of family around age 12** |  |
| Lived comfortably | 3,026 (27%) |
| Got by | 3,279 (29%) |
| Found it difficult | 4,071 (36%) |
| Found it very difficult | 994 (8.7%) |
| Missing | 19 (0.2%) |
| **Experienced abuse when growing up** |  |
| Yes | 1,300 (11%) |
| No | 10,039 (88%) |
| Missing | 49 (0.4%) |
| **Felt like an outsider in the family when growing up** |  |
| Yes | 1,223 (11%) |
| No | 10,114 (89%) |
| Missing | 52 (0.5%) |
| **Self-rated health when growing up** |  |
| Excellent | 4,449 (39%) |
| Very good | 2,598 (23%) |
| Good | 2,582 (23%) |
| Fair | 1,384 (12%) |
| Poor | 349 (3.1%) |
| Missing | 26 (0.2%) |
| **Immigration status** |  |
| Born in this country | 11,270 (99%) |
| Born in another country | 117 (1.0%) |
| Missing | 2 (< 0.1%) |
| **Frequency of religious service attendance around age 12** |  |
| ≥ 1/week | 9,189 (81%) |
| 1-3/month | 1,687 (15%) |
| < 1/month | 236 (2.1%) |
| Never | 198 (1.7%) |
| Missing | 79 (0.7%) |
| **Year of birth** |  |
| 1998-2005 (current age: 18-24 years) | 2,868 (25%) |
| 1993-1998 (current age: 25-29 years) | 2,035 (18%) |
| 1983-1993 (current age: 30-39 years) | 2,564 (23%) |
| 1973-1983 (current age: 40-49 years) | 1,708 (15%) |
| 1963-1973 (current age: 50-59 years) | 1,072 (9.4%) |
| 1953-1963 (current age: 60-69 years) | 710 (6.2%) |
| 1943-1953 (current age: 70-79 years) | 360 (3.2%) |
| 1943 or earlier (current age: 80+ years) | 67 (0.6%) |
| Missing | 5 (< 0.1%) |
| **Gender** |  |
| Male | 5,567 (49%) |
| Female | 5,813 (51%) |
| Other | 2 (< 0.1%) |
| Missing | 7 (< 0.1%) |
| **Religious affiliation at age 12** |  |
| Baha’i | 3 (< 0.1%) |
| Buddhism | 5 (< 0.1%) |
| Christianity | 10,369 (91%) |
| Islam | 916 (8.0%) |
| Jainism | 1 (< 0.1%) |
| Judaism | 6 (< 0.1%) |
| No religion/atheist/agnostic | 67 (0.6%) |
| Primal, animist, or folk religion | 13 (0.1%) |
| Sikhism | 0 (< 0.1%) |
| Some other religion | 0 (< 0.1%) |
| Missing | 9 (< 0.1%) |
| **Racial/ethnic identity** |  |
| Embu | 197 (1.7%) |
| Kalenjin | 1,377 (12%) |
| Kamba | 1,299 (11%) |
| Kenyan Somali/Somali | 396 (3.5%) |
| Kikuyu | 2,119 (19%) |
| Kisii | 789 (6.9%) |
| Luhya | 1,943 (17%) |
| Luo | 1,120 (9.8%) |
| Maasai | 237 (2.1%) |
| Meru | 630 (5.5%) |
| Miji Kenda tribes | 708 (6.2%) |
| Other | 548 (4.8%) |
| Missing | 27 (0.2%) |

| Table S11b  *Regression of Dispositional Forgivingness on Candidate Predictors for Kenya* | | | | | |
| --- | --- | --- | --- | --- | --- |
| Variable | RR (95% CI) | | Global  *p*-value | *E*-values | |
|  |  |  |  | *E*-value for estimate | *E*-value for estimate |
| **Relationship with mother when growing up** |  |  | 0.910 |  |  |
| Ref: Very bad/somewhat bad | - | |  | - | - |
| Very good/somewhat good | 0.99 | (0.93, 1.06) |  | 1.10 | 1.00 |
| **Relationship with father when growing up** |  |  | 0.432 |  |  |
| Ref: Very bad/somewhat bad | - | |  | - | - |
| Very good/somewhat good | 0.99 | (0.94, 1.04) |  | 1.13 | 1.00 |
| **Family structure around age 12** |  |  | 0.004 |  |  |
| Ref: Parents were married | - | |  | - | - |
| Parents were divorced | 0.96 | (0.92, 1.00) |  | 1.26 | 1.06 |
| Parents were never married | 0.96 | (0.92, 1.01) |  | 1.25 | 1.00 |
| One or both parents had died | 1.01 | (0.96, 1.05) |  | 1.08 | 1.00 |
| **Subjective financial status of family around age 12** |  |  | 0.947 |  |  |
| Ref: Got by | - | |  | - | - |
| Lived comfortably | 1.01 | (0.98, 1.04) |  | 1.12 | 1.00 |
| Found it difficult | 1.01 | (0.99, 1.04) |  | 1.13 | 1.00 |
| Found it very difficult | 0.99 | (0.95, 1.04) |  | 1.09 | 1.00 |
| **Experienced abuse when growing up** |  |  | 0.002 |  |  |
| Ref: No | - | |  | - | - |
| Yes | 0.97 | (0.93, 1.00) |  | 1.23 | 1.00 |
| **Felt like an outsider in the family when growing up** |  |  | < 0.001 |  |  |
| Ref: No | - | |  | - | - |
| Yes | 0.95 | (0.91, 0.99) |  | 1.29 | 1.13 |
| **Self-rated health when growing up** |  |  | 0.204 |  |  |
| Ref: Good | - | |  | - | - |
| Excellent | 1.01 | (0.98, 1.03) |  | 1.10 | 1.00 |
| Very good | 1.00 | (0.98, 1.03) |  | 1.06 | 1.00 |
| Fair | 0.97 | (0.94, 1.01) |  | 1.20 | 1.00 |
| Poor | 0.99 | (0.92, 1.06) |  | 1.13 | 1.00 |
| **Immigration status** |  |  | 0.752 |  |  |
| Ref: Born in this country | - | |  | - | - |
| Born in another country | 0.96 | (0.86, 1.08) |  | 1.24 | 1.00 |
| **Frequency of religious service attendance around age 12** |  |  | 0.942 |  |  |
| Ref: Never | - | |  | - | - |
| ≥ 1/week | 1.04 | (0.95, 1.14) |  | 1.25 | 1.00 |
| 1-3/month | 1.04 | (0.95, 1.14) |  | 1.24 | 1.00 |
| < 1/month | 1.01 | (0.90, 1.12) |  | 1.08 | 1.00 |
| **Year of birth** |  |  | < 0.001 |  |  |
| Ref: 1998-2005 (current age: 18-24 years) | - | |  | - | - |
| 1993-1998 (current age: 25-29 years) | 1.03 | (1.01, 1.05) |  | 1.20 | 1.08 |
| 1983-1993 (current age: 30-39 years) | 1.03 | (1.01, 1.06) |  | 1.22 | 1.10 |
| 1973-1983 (current age: 40-49 years) | 1.05 | (1.02, 1.08) |  | 1.27 | 1.14 |
| 1963-1973 (current age: 50-59 years) | 1.01 | (0.97, 1.06) |  | 1.13 | 1.00 |
| 1953-1963 (current age: 60-69 years) | 1.06 | (1.02, 1.11) |  | 1.33 | 1.17 |
| 1943-1953 (current age: 70-79 years) | 1.11 | (1.05, 1.17) |  | 1.45 | 1.26 |
| 1943 or earlier (current age: 80+ years) | 1.09 | (0.96, 1.23) |  | 1.40 | 1.00 |
| **Gender** |  |  | < 0.001 |  |  |
| Ref: Male | - | |  | - | - |
| Female | 0.98 | (0.97, 1.00) |  | 1.15 | 1.00 |
| Other | 1.20 | (1.14, 1.25) |  | 1.68 | 1.54 |
| **Religious affiliation at age 12** |  |  | 0.992 |  |  |
| Ref: Christianity | - | |  | - | - |
| Islam | 1.00 | (0.96, 1.04) |  | 1.03 | 1.00 |
| Some other religion | 1.04 | (0.93, 1.17) |  | 1.25 | 1.00 |
| **Racial/ethnic identity** |  |  | < 0.001 |  |  |
| Ref: Majority | - | |  | - | - |
| Minority | 0.95 | (0.93, 0.98) |  | 1.27 | 1.16 |

| Table S12a  *Nationally Representative Descriptive Statistics for Mexico (N = 5,776)* | |
| --- | --- |
| Characteristic | *n* (%) |
| **Relationship with mother when growing up** |  |
| Very good | 3,912 (68%) |
| Somewhat good | 1,340 (23%) |
| Somewhat bad | 177 (3.1%) |
| Very bad | 90 (1.6%) |
| Does not apply | 177 (3.1%) |
| Missing | 80 (1.4%) |
| **Relationship with father when growing up** |  |
| Very good | 3,089 (53%) |
| Somewhat good | 1,556 (27%) |
| Somewhat bad | 335 (5.8%) |
| Very bad | 267 (4.6%) |
| Does not apply | 470 (8.1%) |
| Missing | 60 (1.0%) |
| **Family structure around age 12** |  |
| Parents were married | 3,999 (69%) |
| Parents were divorced | 341 (5.9%) |
| Parents were never married | 827 (14%) |
| One or both parents had died | 176 (3.0%) |
| Missing | 432 (7.5%) |
| **Subjective financial status of family around age 12** |  |
| Lived comfortably | 1,775 (31%) |
| Got by | 1,872 (32%) |
| Found it difficult | 1,712 (30%) |
| Found it very difficult | 369 (6.4%) |
| Missing | 48 (0.8%) |
| **Experienced abuse when growing up** |  |
| Yes | 905 (16%) |
| No | 4,604 (80%) |
| Missing | 267 (4.6%) |
| **Felt like an outsider in the family when growing up** |  |
| Yes | 772 (13%) |
| No | 4,897 (85%) |
| Missing | 107 (1.9%) |
| **Self-rated health when growing up** |  |
| Excellent | 1,860 (32%) |
| Very good | 1,350 (23%) |
| Good | 1,677 (29%) |
| Fair | 743 (13%) |
| Poor | 133 (2.3%) |
| Missing | 14 (0.2%) |
| **Immigration status** |  |
| Born in this country | 5,517 (96%) |
| Born in another country | 108 (1.9%) |
| Missing | 151 (2.6%) |
| **Frequency of religious service attendance around age 12** |  |
| ≥ 1/week | 2,514 (44%) |
| 1-3/month | 1,162 (20%) |
| < 1/month | 1,087 (19%) |
| Never | 944 (16%) |
| Missing | 69 (1.2%) |
| **Year of birth** |  |
| 1998-2005 (current age: 18-24 years) | 986 (17%) |
| 1993-1998 (current age: 25-29 years) | 623 (11%) |
| 1983-1993 (current age: 30-39 years) | 1,312 (23%) |
| 1973-1983 (current age: 40-49 years) | 1,027 (18%) |
| 1963-1973 (current age: 50-59 years) | 873 (15%) |
| 1953-1963 (current age: 60-69 years) | 611 (11%) |
| 1943-1953 (current age: 70-79 years) | 277 (4.8%) |
| 1943 or earlier (current age: 80+ years) | 68 (1.2%) |
| Missing | 0 (0%) |
| **Gender** |  |
| Male | 2,755 (48%) |
| Female | 2,997 (52%) |
| Other | 3 (< 0.1%) |
| Missing | 21 (0.4%) |
| **Religious affiliation at age 12** |  |
| Baha’i | 1 (< 0.1%) |
| Buddhism | 1 (< 0.1%) |
| Christianity | 5,337 (92%) |
| Hinduism | 1 (< 0.1%) |
| Islam | 6 (< 0.1%) |
| Judaism | 8 (0.1%) |
| No religion/atheist/agnostic | 328 (5.7%) |
| Primal, animist, or folk religion | 2 (< 0.1%) |
| Shinto | 2 (< 0.1%) |
| Sikhism | 4 (< 0.1%) |
| Some other religion | 7 (0.1%) |
| Taoism | 5 (< 0.1%) |
| Missing | 74 (1.3%) |
| **Racial/ethnic identity** |  |
| Black | 108 (1.9%) |
| Indigenous | 594 (10%) |
| Mestizo | 2,762 (48%) |
| Mulatto | 63 (1.1%) |
| Other | 339 (5.9%) |
| White | 1,116 (19%) |
| Missing | 794 (14%) |

| Table S12b  *Regression of Dispositional Forgivingness on Candidate Predictors for Mexico* | | | | | |
| --- | --- | --- | --- | --- | --- |
| Variable | RR (95% CI) | | Global  *p*-value | *E*-values | |
|  |  |  |  | *E*-value for estimate | *E*-value for estimate |
| **Relationship with mother when growing up** |  |  | 0.009 |  |  |
| Ref: Very bad/somewhat bad | - | |  | - | - |
| Very good/somewhat good | 1.04 | (0.96, 1.14) |  | 1.27 | 1.00 |
| **Relationship with father when growing up** |  |  | 0.986 |  |  |
| Ref: Very bad/somewhat bad | - | |  | - | - |
| Very good/somewhat good | 1.00 | (0.94, 1.06) |  | 1.06 | 1.00 |
| **Family structure around age 12** |  |  | 0.060 |  |  |
| Ref: Parents were married | - | |  | - | - |
| Parents were divorced | 1.07 | (1.01, 1.15) |  | 1.36 | 1.08 |
| Parents were never married | 1.00 | (0.94, 1.06) |  | 1.04 | 1.00 |
| One or both parents had died | 1.01 | (0.91, 1.12) |  | 1.10 | 1.00 |
| **Subjective financial status of family around age 12** |  |  | 0.073 |  |  |
| Ref: Got by | - | |  | - | - |
| Lived comfortably | 1.04 | (0.99, 1.09) |  | 1.25 | 1.00 |
| Found it difficult | 1.00 | (0.95, 1.05) |  | 1.06 | 1.00 |
| Found it very difficult | 0.99 | (0.91, 1.08) |  | 1.08 | 1.00 |
| **Experienced abuse when growing up** |  |  | 0.004 |  |  |
| Ref: No | - | |  | - | - |
| Yes | 1.05 | (0.99, 1.10) |  | 1.27 | 1.00 |
| **Felt like an outsider in the family when growing up** |  |  | 0.151 |  |  |
| Ref: No | - | |  | - | - |
| Yes | 0.96 | (0.91, 1.02) |  | 1.23 | 1.00 |
| **Self-rated health when growing up** |  |  | 0.085 |  |  |
| Ref: Good | - | |  | - | - |
| Excellent | 1.05 | (1.00, 1.10) |  | 1.27 | 1.03 |
| Very good | 1.01 | (0.96, 1.06) |  | 1.10 | 1.00 |
| Fair | 0.98 | (0.92, 1.05) |  | 1.15 | 1.00 |
| Poor | 0.99 | (0.86, 1.15) |  | 1.10 | 1.00 |
| **Immigration status** |  |  | 1.000 |  |  |
| Ref: Born in this country | - | |  | - | - |
| Born in another country | 1.00 | (0.86, 1.17) |  | 1.07 | 1.00 |
| **Frequency of religious service attendance around age 12** |  |  | < 0.001 |  |  |
| Ref: Never | - | |  | - | - |
| ≥ 1/week | 1.14 | (1.07, 1.22) |  | 1.55 | 1.35 |
| 1-3/month | 1.09 | (1.02, 1.18) |  | 1.42 | 1.17 |
| < 1/month | 1.05 | (0.97, 1.13) |  | 1.27 | 1.00 |
| **Year of birth** |  |  | < 0.001 |  |  |
| Ref: 1998-2005 (current age: 18-24 years) | - | |  | - | - |
| 1993-1998 (current age: 25-29 years) | 1.13 | (1.04, 1.23) |  | 1.52 | 1.26 |
| 1983-1993 (current age: 30-39 years) | 1.14 | (1.06, 1.23) |  | 1.55 | 1.32 |
| 1973-1983 (current age: 40-49 years) | 1.20 | (1.12, 1.29) |  | 1.69 | 1.48 |
| 1963-1973 (current age: 50-59 years) | 1.25 | (1.16, 1.34) |  | 1.80 | 1.58 |
| 1953-1963 (current age: 60-69 years) | 1.32 | (1.23, 1.42) |  | 1.97 | 1.76 |
| 1943-1953 (current age: 70-79 years) | 1.16 | (1.03, 1.31) |  | 1.60 | 1.20 |
| 1943 or earlier (current age: 80+ years) | 1.13 | (0.89, 1.43) |  | 1.51 | 1.00 |
| **Gender** |  |  | 1.000 |  |  |
| Ref: Male | - | |  | - | - |
| Female | 1.00 | (0.96, 1.04) |  | 1.05 | 1.00 |
| Other | 0.89 | (0.50, 1.57) |  | 1.51 | 1.00 |
| **Religious affiliation at age 12** |  |  | 0.098 |  |  |
| Ref: No religion/atheist/agnostic | - | |  | - | - |
| Christianity | 1.08 | (0.97, 1.21) |  | 1.39 | 1.00 |
| Some other religion | 1.17 | (0.95, 1.43) |  | 1.61 | 1.00 |
| **Racial/ethnic identity** |  |  | < 0.001 |  |  |
| Ref: Majority | - | |  | - | - |
| Minority | 0.92 | (0.89, 0.96) |  | 1.38 | 1.25 |

| Table S13a  *Nationally Representative Descriptive Statistics for Nigeria (N = 6,827)* | |
| --- | --- |
| Characteristic | *n* (%) |
| **Relationship with mother when growing up** |  |
| Very good | 5,986 (88%) |
| Somewhat good | 648 (9.5%) |
| Somewhat bad | 62 (0.9%) |
| Very bad | 18 (0.3%) |
| Does not apply | 104 (1.5%) |
| Missing | 9 (0.1%) |
| **Relationship with father when growing up** |  |
| Very good | 5,578 (82%) |
| Somewhat good | 924 (14%) |
| Somewhat bad | 76 (1.1%) |
| Very bad | 43 (0.6%) |
| Does not apply | 177 (2.6%) |
| Missing | 29 (0.4%) |
| **Family structure around age 12** |  |
| Parents were married | 5,568 (82%) |
| Parents were divorced | 307 (4.5%) |
| Parents were never married | 335 (4.9%) |
| One or both parents had died | 462 (6.8%) |
| Missing | 154 (2.3%) |
| **Subjective financial status of family around age 12** |  |
| Lived comfortably | 2,192 (32%) |
| Got by | 2,381 (35%) |
| Found it difficult | 1,661 (24%) |
| Found it very difficult | 563 (8.3%) |
| Missing | 29 (0.4%) |
| **Experienced abuse when growing up** |  |
| Yes | 880 (13%) |
| No | 5,851 (86%) |
| Missing | 96 (1.4%) |
| **Felt like an outsider in the family when growing up** |  |
| Yes | 669 (9.8%) |
| No | 6,059 (89%) |
| Missing | 99 (1.5%) |
| **Self-rated health when growing up** |  |
| Excellent | 2,644 (39%) |
| Very good | 2,613 (38%) |
| Good | 1,152 (17%) |
| Fair | 306 (4.5%) |
| Poor | 98 (1.4%) |
| Missing | 14 (0.2%) |
| **Immigration status** |  |
| Born in this country | 6,779 (99%) |
| Born in another country | 47 (0.7%) |
| Missing | 1 (< 0.1%) |
| **Frequency of religious service attendance around age 12** |  |
| ≥ 1/week | 5,907 (87%) |
| 1-3/month | 600 (8.8%) |
| < 1/month | 136 (2.0%) |
| Never | 138 (2.0%) |
| Missing | 45 (0.7%) |
| **Year of birth** |  |
| 1998-2005 (current age: 18-24 years) | 1,533 (22%) |
| 1993-1998 (current age: 25-29 years) | 1,193 (17%) |
| 1983-1993 (current age: 30-39 years) | 1,943 (28%) |
| 1973-1983 (current age: 40-49 years) | 1,059 (16%) |
| 1963-1973 (current age: 50-59 years) | 619 (9.1%) |
| 1953-1963 (current age: 60-69 years) | 296 (4.3%) |
| 1943-1953 (current age: 70-79 years) | 133 (2.0%) |
| 1943 or earlier (current age: 80+ years) | 50 (0.7%) |
| Missing | 0 (0%) |
| **Gender** |  |
| Male | 3,371 (49%) |
| Female | 3,456 (51%) |
| Other | 0 (< 0.1%) |
| Missing | 0 (0%) |
| **Religious affiliation at age 12** |  |
| Buddhism | 0 (< 0.1%) |
| Christianity | 3,463 (51%) |
| Confucianism | 0 (< 0.1%) |
| Islam | 3,314 (49%) |
| No religion/atheist/agnostic | 19 (0.3%) |
| Primal, animist, or folk religion | 17 (0.3%) |
| Missing | 14 (0.2%) |
| **Racial/ethnic identity** |  |
| Edo | 116 (1.7%) |
| Efik | 48 (0.7%) |
| Fulani | 266 (3.9%) |
| Hausa | 2,342 (34%) |
| Ibibio | 180 (2.6%) |
| Idoma | 61 (0.9%) |
| Igala | 77 (1.1%) |
| Igbo (Ibo) | 1,111 (16%) |
| Ijaw | 110 (1.6%) |
| Kanuri | 31 (0.5%) |
| Other | 1,014 (15%) |
| Tiv | 198 (2.9%) |
| Urhobo | 38 (0.6%) |
| Yoruba | 1,230 (18%) |
| Missing | 4 (< 0.1%) |

| Table S13b  *Regression of Dispositional Forgivingness on Candidate Predictors for Nigeria* | | | | | | |
| --- | --- | --- | --- | --- | --- | --- |
| Variable | RR (95% CI) | | Global  *p*-value | *E*-values | |  |
|  |  |  |  | *E*-value for estimate | *E*-value for estimate |  |
| **Relationship with mother when growing up** |  |  | < 0.001 |  |  |  |
| Ref: Very bad/somewhat bad | - | |  | - | - |  |
| Very good/somewhat good | 1.12 | (1.02, 1.21) |  | 1.47 | 1.18 |  |
| **Relationship with father when growing up** |  |  | 0.673 |  |  |  |
| Ref: Very bad/somewhat bad | - | |  | - | - |  |
| Very good/somewhat good | 1.02 | (0.92, 1.13) |  | 1.17 | 1.00 |  |
| **Family structure around age 12** |  |  | < 0.001 |  |  |  |
| Ref: Parents were married | - | |  | - | - |  |
| Parents were divorced | 0.90 | (0.83, 0.97) |  | 1.47 | 1.21 |  |
| Parents were never married | 0.99 | (0.95, 1.03) |  | 1.11 | 1.00 |  |
| One or both parents had died | 0.93 | (0.88, 0.98) |  | 1.36 | 1.17 |  |
| **Subjective financial status of family around age 12** |  |  | 1.000 |  |  |  |
| Ref: Got by | - | |  | - | - |  |
| Lived comfortably | 1.01 | (0.98, 1.03) |  | 1.08 | 1.00 |  |
| Found it difficult | 1.00 | (0.97, 1.03) |  | 1.05 | 1.00 |  |
| Found it very difficult | 0.99 | (0.95, 1.03) |  | 1.10 | 1.00 |  |
| **Experienced abuse when growing up** |  |  | 0.046 |  |  |  |
| Ref: No | - | |  | - | - |  |
| Yes | 0.98 | (0.94, 1.01) |  | 1.18 | 1.00 |  |
| **Felt like an outsider in the family when growing up** |  |  | 0.005 |  |  |  |
| Ref: No | - | |  | - | - |  |
| Yes | 0.96 | (0.93, 1.00) |  | 1.23 | 1.00 |  |
| **Self-rated health when growing up** |  |  | < 0.001 |  |  |  |
| Ref: Good | - | |  | - | - |  |
| Excellent | 0.99 | (0.96, 1.03) |  | 1.09 | 1.00 |  |
| Very good | 1.00 | (0.97, 1.03) |  | 1.07 | 1.00 |  |
| Fair | 1.06 | (1.02, 1.10) |  | 1.31 | 1.17 |  |
| Poor | 1.03 | (0.97, 1.09) |  | 1.20 | 1.00 |  |
| **Immigration status** |  |  | 0.022 |  |  |  |
| Ref: Born in this country | - | |  | - | - |  |
| Born in another country | 1.04 | (0.99, 1.10) |  | 1.26 | 1.00 |  |
| **Frequency of religious service attendance around age 12** |  |  | 1.000 |  |  |  |
| Ref: Never | - | |  | - | - |  |
| ≥ 1/week | 1.00 | (0.93, 1.08) |  | 1.05 | 1.00 |  |
| 1-3/month | 1.01 | (0.93, 1.09) |  | 1.08 | 1.00 |  |
| < 1/month | 0.99 | (0.89, 1.09) |  | 1.12 | 1.00 |  |
| **Year of birth** |  |  | < 0.001 |  |  |  |
| Ref: 1998-2005 (current age: 18-24 years) | - | |  | - | - |  |
| 1993-1998 (current age: 25-29 years) | 1.02 | (0.99, 1.05) |  | 1.16 | 1.00 |  |
| 1983-1993 (current age: 30-39 years) | 1.01 | (0.99, 1.04) |  | 1.13 | 1.00 |  |
| 1973-1983 (current age: 40-49 years) | 1.04 | (1.01, 1.07) |  | 1.24 | 1.09 |  |
| 1963-1973 (current age: 50-59 years) | 1.06 | (1.02, 1.10) |  | 1.31 | 1.16 |  |
| 1953-1963 (current age: 60-69 years) | 0.94 | (0.86, 1.03) |  | 1.31 | 1.00 |  |
| 1943-1953 (current age: 70-79 years) | 1.04 | (0.94, 1.15) |  | 1.23 | 1.00 |  |
| 1943 or earlier (current age: 80+ years) | 0.90 | (0.75, 1.08) |  | 1.47 | 1.00 |  |
| **Gender** |  |  | < 0.001 |  |  |  |
| Ref: Male | - | |  | - | - |  |
| Female | 1.00 | (0.99, 1.02) |  | 1.07 | 1.00 |  |
| Other | 1.09 | (1.05, 1.14) |  | 1.43 | 1.29 |  |
| **Religious affiliation at age 12** |  |  | 0.481 |  |  |  |
| Ref: Christianity | - | |  | - | - |  |
| Islam | 1.01 | (0.99, 1.04) |  | 1.13 | 1.00 |  |
| Some other religion | 1.06 | (0.94, 1.19) |  | 1.31 | 1.00 |  |
| **Racial/ethnic identity** |  |  | 0.044 |  |  |  |
| Ref: Majority | - | |  | - | - |  |
| Minority | 1.02 | (0.99, 1.05) |  | 1.17 | 1.00 |  |

| Table S14a  *Nationally Representative Descriptive Statistics for Philippines (N = 5,292)* | |
| --- | --- |
| Characteristic | *n* (%) |
| **Relationship with mother when growing up** |  |
| Very good | 3,333 (63%) |
| Somewhat good | 1,703 (32%) |
| Somewhat bad | 124 (2.3%) |
| Very bad | 39 (0.7%) |
| Does not apply | 59 (1.1%) |
| Missing | 35 (0.7%) |
| **Relationship with father when growing up** |  |
| Very good | 3,443 (65%) |
| Somewhat good | 1,429 (27%) |
| Somewhat bad | 159 (3.0%) |
| Very bad | 58 (1.1%) |
| Does not apply | 108 (2.0%) |
| Missing | 95 (1.8%) |
| **Family structure around age 12** |  |
| Parents were married | 4,575 (86%) |
| Parents were divorced | 64 (1.2%) |
| Parents were never married | 517 (9.8%) |
| One or both parents had died | 51 (1.0%) |
| Missing | 85 (1.6%) |
| **Subjective financial status of family around age 12** |  |
| Lived comfortably | 937 (18%) |
| Got by | 3,006 (57%) |
| Found it difficult | 1,055 (20%) |
| Found it very difficult | 291 (5.5%) |
| Missing | 3 (< 0.1%) |
| **Experienced abuse when growing up** |  |
| Yes | 420 (7.9%) |
| No | 4,837 (91%) |
| Missing | 35 (0.7%) |
| **Felt like an outsider in the family when growing up** |  |
| Yes | 395 (7.5%) |
| No | 4,884 (92%) |
| Missing | 13 (0.2%) |
| **Self-rated health when growing up** |  |
| Excellent | 1,041 (20%) |
| Very good | 559 (11%) |
| Good | 2,174 (41%) |
| Fair | 1,246 (24%) |
| Poor | 272 (5.1%) |
| Missing | 0 (< 0.1%) |
| **Immigration status** |  |
| Born in this country | 5,284 (100%) |
| Born in another country | 8 (0.1%) |
| Missing | 0 (0%) |
| **Frequency of religious service attendance around age 12** |  |
| ≥ 1/week | 2,453 (46%) |
| 1-3/month | 1,699 (32%) |
| < 1/month | 892 (17%) |
| Never | 201 (3.8%) |
| Missing | 47 (0.9%) |
| **Year of birth** |  |
| 1998-2005 (current age: 18-24 years) | 1,073 (20%) |
| 1993-1998 (current age: 25-29 years) | 695 (13%) |
| 1983-1993 (current age: 30-39 years) | 1,160 (22%) |
| 1973-1983 (current age: 40-49 years) | 972 (18%) |
| 1963-1973 (current age: 50-59 years) | 732 (14%) |
| 1953-1963 (current age: 60-69 years) | 495 (9.4%) |
| 1943-1953 (current age: 70-79 years) | 143 (2.7%) |
| 1943 or earlier (current age: 80+ years) | 23 (0.4%) |
| Missing | 0 (0%) |
| **Gender** |  |
| Male | 2,625 (50%) |
| Female | 2,643 (50%) |
| Other | 13 (0.2%) |
| Missing | 11 (0.2%) |
| **Religious affiliation at age 12** |  |
| Baha’i | 1 (< 0.1%) |
| Buddhism | 1 (< 0.1%) |
| Christianity | 4,968 (94%) |
| Islam | 276 (5.2%) |
| No religion/atheist/agnostic | 9 (0.2%) |
| Primal, animist, or folk religion | 14 (0.3%) |
| Sikhism | 4 (< 0.1%) |
| Some other religion | 9 (0.2%) |
| Missing | 11 (0.2%) |
| **Racial/ethnic identity** |  |
| Aeta | 1 (< 0.1%) |
| Badjao | 2 (< 0.1%) |
| Bicolano/Bikolano | 300 (5.7%) |
| Cebuano | 656 (12%) |
| Chinese-Filipino | 3 (< 0.1%) |
| Igorot | 42 (0.8%) |
| Ilocano/Ilokano | 429 (8.1%) |
| Ilonggo/Hiligaynon | 428 (8.1%) |
| Kapampangan | 107 (2.0%) |
| Maguindanaoan | 84 (1.6%) |
| Mangyan | 2 (< 0.1%) |
| Maranao | 39 (0.7%) |
| Masbateno | 54 (1.0%) |
| Other | 244 (4.6%) |
| Pangasinense | 107 (2.0%) |
| Tagalog | 1,691 (32%) |
| Tausug | 94 (1.8%) |
| Visayan/Bisaya | 739 (14%) |
| Waray | 216 (4.1%) |
| Zamboangueno | 51 (1.0%) |
| Missing | 3 (< 0.1%) |

| Table S14b  *Regression of Dispositional Forgivingness on Candidate Predictors for Philippines* | | | | | |
| --- | --- | --- | --- | --- | --- |
| Variable | RR (95% CI) | | Global  *p*-value | *E*-values | |
|  |  |  |  | *E*-value for estimate | *E*-value for estimate |
| **Relationship with mother when growing up** |  |  | 0.460 |  |  |
| Ref: Very bad/somewhat bad | - | |  | - | - |
| Very good/somewhat good | 1.03 | (0.92, 1.14) |  | 1.20 | 1.00 |
| **Relationship with father when growing up** |  |  | 0.943 |  |  |
| Ref: Very bad/somewhat bad | - | |  | - | - |
| Very good/somewhat good | 1.01 | (0.92, 1.11) |  | 1.11 | 1.00 |
| **Family structure around age 12** |  |  | < 0.001 |  |  |
| Ref: Parents were married | - | |  | - | - |
| Parents were divorced | 1.04 | (0.91, 1.19) |  | 1.24 | 1.00 |
| Parents were never married | 0.99 | (0.93, 1.06) |  | 1.10 | 1.00 |
| One or both parents had died | 1.16 | (1.06, 1.28) |  | 1.61 | 1.32 |
| **Subjective financial status of family around age 12** |  |  | 0.519 |  |  |
| Ref: Got by | - | |  | - | - |
| Lived comfortably | 1.01 | (0.96, 1.07) |  | 1.13 | 1.00 |
| Found it difficult | 1.04 | (0.99, 1.08) |  | 1.23 | 1.00 |
| Found it very difficult | 0.98 | (0.88, 1.08) |  | 1.18 | 1.00 |
| **Experienced abuse when growing up** |  |  | 0.020 |  |  |
| Ref: No | - | |  | - | - |
| Yes | 0.93 | (0.86, 1.02) |  | 1.35 | 1.00 |
| **Felt like an outsider in the family when growing up** |  |  | < 0.001 |  |  |
| Ref: No | - | |  | - | - |
| Yes | 0.90 | (0.82, 0.99) |  | 1.45 | 1.11 |
| **Self-rated health when growing up** |  |  | 0.846 |  |  |
| Ref: Good | - | |  | - | - |
| Excellent | 0.98 | (0.93, 1.03) |  | 1.18 | 1.00 |
| Very good | 0.98 | (0.92, 1.05) |  | 1.15 | 1.00 |
| Fair | 0.96 | (0.92, 1.02) |  | 1.23 | 1.00 |
| Poor | 1.02 | (0.94, 1.10) |  | 1.15 | 1.00 |
| **Immigration status** |  |  | 0.962 |  |  |
| Ref: Born in this country | - | |  | - | - |
| Born in another country | 0.88 | (0.45, 1.70) |  | 1.55 | 1.00 |
| **Frequency of religious service attendance around age 12** |  |  | 0.115 |  |  |
| Ref: Never | - | |  | - | - |
| ≥ 1/week | 1.09 | (0.96, 1.25) |  | 1.42 | 1.00 |
| 1-3/month | 1.08 | (0.94, 1.25) |  | 1.39 | 1.00 |
| < 1/month | 1.04 | (0.90, 1.20) |  | 1.24 | 1.00 |
| **Year of birth** |  |  | 0.002 |  |  |
| Ref: 1998-2005 (current age: 18-24 years) | - | |  | - | - |
| 1993-1998 (current age: 25-29 years) | 1.09 | (1.02, 1.18) |  | 1.43 | 1.17 |
| 1983-1993 (current age: 30-39 years) | 1.03 | (0.97, 1.10) |  | 1.21 | 1.00 |
| 1973-1983 (current age: 40-49 years) | 1.06 | (0.99, 1.13) |  | 1.30 | 1.00 |
| 1963-1973 (current age: 50-59 years) | 0.98 | (0.91, 1.07) |  | 1.14 | 1.00 |
| 1953-1963 (current age: 60-69 years) | 1.04 | (0.96, 1.13) |  | 1.27 | 1.00 |
| 1943-1953 (current age: 70-79 years) | 0.93 | (0.80, 1.09) |  | 1.34 | 1.00 |
| 1943 or earlier (current age: 80+ years) | 1.05 | (0.79, 1.39) |  | 1.27 | 1.00 |
| **Gender** |  |  | 0.042 |  |  |
| Ref: Male | - | |  | - | - |
| Female | 0.97 | (0.93, 1.01) |  | 1.22 | 1.00 |
| Other | 0.78 | (0.50, 1.20) |  | 1.89 | 1.00 |
| **Religious affiliation at age 12** |  |  | 0.976 |  |  |
| Ref: Christianity | - | |  | - | - |
| Islam | 0.97 | (0.90, 1.05) |  | 1.20 | 1.00 |
| Some other religion | 1.05 | (0.80, 1.37) |  | 1.27 | 1.00 |
| **Racial/ethnic identity** |  |  | 0.123 |  |  |
| Ref: Majority | - | |  | - | - |
| Minority | 0.97 | (0.93, 1.02) |  | 1.19 | 1.00 |

| Table S15a  *Nationally Representative Descriptive Statistics for Poland (N = 10,389)* | |
| --- | --- |
| Characteristic | *n* (%) |
| **Relationship with mother when growing up** |  |
| Very good | 4,879 (47%) |
| Somewhat good | 4,973 (48%) |
| Somewhat bad | 285 (2.7%) |
| Very bad | 58 (0.6%) |
| Does not apply | 80 (0.8%) |
| Missing | 112 (1.1%) |
| **Relationship with father when growing up** |  |
| Very good | 4,231 (41%) |
| Somewhat good | 4,984 (48%) |
| Somewhat bad | 516 (5.0%) |
| Very bad | 78 (0.7%) |
| Does not apply | 407 (3.9%) |
| Missing | 173 (1.7%) |
| **Family structure around age 12** |  |
| Parents were married | 8,972 (86%) |
| Parents were divorced | 587 (5.7%) |
| Parents were never married | 193 (1.9%) |
| One or both parents had died | 313 (3.0%) |
| Missing | 324 (3.1%) |
| **Subjective financial status of family around age 12** |  |
| Lived comfortably | 1,384 (13%) |
| Got by | 6,257 (60%) |
| Found it difficult | 2,133 (21%) |
| Found it very difficult | 509 (4.9%) |
| Missing | 106 (1.0%) |
| **Experienced abuse when growing up** |  |
| Yes | 325 (3.1%) |
| No | 10,009 (96%) |
| Missing | 55 (0.5%) |
| **Felt like an outsider in the family when growing up** |  |
| Yes | 490 (4.7%) |
| No | 9,615 (93%) |
| Missing | 284 (2.7%) |
| **Self-rated health when growing up** |  |
| Excellent | 2,676 (26%) |
| Very good | 5,371 (52%) |
| Good | 1,779 (17%) |
| Fair | 406 (3.9%) |
| Poor | 123 (1.2%) |
| Missing | 34 (0.3%) |
| **Immigration status** |  |
| Born in this country | 10,258 (99%) |
| Born in another country | 108 (1.0%) |
| Missing | 23 (0.2%) |
| **Frequency of religious service attendance around age 12** |  |
| ≥ 1/week | 4,751 (46%) |
| 1-3/month | 2,689 (26%) |
| < 1/month | 2,161 (21%) |
| Never | 354 (3.4%) |
| Missing | 434 (4.2%) |
| **Year of birth** |  |
| 1998-2005 (current age: 18-24 years) | 955 (9.2%) |
| 1993-1998 (current age: 25-29 years) | 761 (7.3%) |
| 1983-1993 (current age: 30-39 years) | 2,159 (21%) |
| 1973-1983 (current age: 40-49 years) | 1,956 (19%) |
| 1963-1973 (current age: 50-59 years) | 1,670 (16%) |
| 1953-1963 (current age: 60-69 years) | 1,909 (18%) |
| 1943-1953 (current age: 70-79 years) | 833 (8.0%) |
| 1943 or earlier (current age: 80+ years) | 145 (1.4%) |
| Missing | 1 (< 0.1%) |
| **Gender** |  |
| Male | 4,974 (48%) |
| Female | 5,387 (52%) |
| Other | 3 (< 0.1%) |
| Missing | 26 (0.2%) |
| **Religious affiliation at age 12** |  |
| Buddhism | 2 (< 0.1%) |
| Christianity | 9,861 (95%) |
| Islam | 3 (< 0.1%) |
| No religion/atheist/agnostic | 482 (4.6%) |
| Primal, animist, or folk religion | 5 (< 0.1%) |
| Sikhism | 1 (< 0.1%) |
| Missing | 35 (0.3%) |
| **Racial/ethnic identity** |  |
| Belarussian | 2 (< 0.1%) |
| German | 4 (< 0.1%) |
| Kashubians | 3 (< 0.1%) |
| Other | 4 (< 0.1%) |
| Polish | 10,309 (99%) |
| Silesia | 14 (0.1%) |
| Ukrainian | 38 (0.4%) |
| Missing | 14 (0.1%) |

| Table S15b  *Regression of Dispositional Forgivingness on Candidate Predictors for Poland* | | | | | |
| --- | --- | --- | --- | --- | --- |
| Variable | RR (95% CI) | | Global  *p*-value | *E*-values | |
|  |  |  |  | *E*-value for estimate | *E*-value for estimate |
| **Relationship with mother when growing up** |  |  | 0.002 |  |  |
| Ref: Very bad/somewhat bad | - | |  | - | - |
| Very good/somewhat good | 1.11 | (0.93, 1.30) |  | 1.44 | 1.00 |
| **Relationship with father when growing up** |  |  | 0.005 |  |  |
| Ref: Very bad/somewhat bad | - | |  | - | - |
| Very good/somewhat good | 1.07 | (0.94, 1.23) |  | 1.36 | 1.00 |
| **Family structure around age 12** |  |  | 1.000 |  |  |
| Ref: Parents were married | - | |  | - | - |
| Parents were divorced | 0.99 | (0.89, 1.10) |  | 1.09 | 1.00 |
| Parents were never married | 1.01 | (0.84, 1.21) |  | 1.12 | 1.00 |
| One or both parents had died | 1.00 | (0.87, 1.15) |  | 1.02 | 1.00 |
| **Subjective financial status of family around age 12** |  |  | 0.326 |  |  |
| Ref: Got by | - | |  | - | - |
| Lived comfortably | 0.99 | (0.92, 1.06) |  | 1.14 | 1.00 |
| Found it difficult | 1.01 | (0.96, 1.07) |  | 1.12 | 1.00 |
| Found it very difficult | 0.90 | (0.81, 1.02) |  | 1.44 | 1.00 |
| **Experienced abuse when growing up** |  |  | 0.843 |  |  |
| Ref: No | - | |  | - | - |
| Yes | 1.03 | (0.92, 1.16) |  | 1.21 | 1.00 |
| **Felt like an outsider in the family when growing up** |  |  | 0.929 |  |  |
| Ref: No | - | |  | - | - |
| Yes | 0.97 | (0.86, 1.10) |  | 1.19 | 1.00 |
| **Self-rated health when growing up** |  |  | < 0.001 |  |  |
| Ref: Good | - | |  | - | - |
| Excellent | 1.11 | (1.03, 1.19) |  | 1.45 | 1.20 |
| Very good | 1.01 | (0.95, 1.08) |  | 1.13 | 1.00 |
| Fair | 1.06 | (0.94, 1.20) |  | 1.31 | 1.00 |
| Poor | 0.92 | (0.64, 1.35) |  | 1.38 | 1.00 |
| **Immigration status** |  |  | < 0.001 |  |  |
| Ref: Born in this country | - | |  | - | - |
| Born in another country | 1.22 | (1.04, 1.44) |  | 1.75 | 1.25 |
| **Frequency of religious service attendance around age 12** |  |  | < 0.001 |  |  |
| Ref: Never | - | |  | - | - |
| ≥ 1/week | 1.31 | (1.10, 1.55) |  | 1.94 | 1.42 |
| 1-3/month | 1.25 | (1.05, 1.48) |  | 1.80 | 1.27 |
| < 1/month | 1.09 | (0.92, 1.30) |  | 1.42 | 1.00 |
| **Year of birth** |  |  | < 0.001 |  |  |
| Ref: 1998-2005 (current age: 18-24 years) | - | |  | - | - |
| 1993-1998 (current age: 25-29 years) | 1.00 | (0.90, 1.11) |  | 1.02 | 1.00 |
| 1983-1993 (current age: 30-39 years) | 1.07 | (0.97, 1.19) |  | 1.36 | 1.00 |
| 1973-1983 (current age: 40-49 years) | 1.07 | (0.97, 1.20) |  | 1.36 | 1.00 |
| 1963-1973 (current age: 50-59 years) | 1.08 | (0.97, 1.22) |  | 1.40 | 1.00 |
| 1953-1963 (current age: 60-69 years) | 1.25 | (1.11, 1.39) |  | 1.80 | 1.47 |
| 1943-1953 (current age: 70-79 years) | 1.39 | (1.24, 1.58) |  | 2.14 | 1.78 |
| 1943 or earlier (current age: 80+ years) | 1.17 | (0.93, 1.50) |  | 1.64 | 1.00 |
| **Gender** |  |  | < 0.001 |  |  |
| Ref: Male | - | |  | - | - |
| Female | 1.15 | (1.10, 1.20) |  | 1.56 | 1.43 |
| Other | 0.83 | (0.36, 1.89) |  | 1.71 | 1.00 |
| **Religious affiliation at age 12** |  |  | 0.243 |  |  |
| Ref: No religion/atheist/agnostic | - | |  | - | - |
| Christianity | 0.91 | (0.81, 1.04) |  | 1.40 | 1.00 |
| Some other religion | 1.06 | (0.72, 1.57) |  | 1.31 | 1.00 |
| **Racial/ethnic identity** |  |  | 1.000 |  |  |
| Ref: Majority | - | |  | - | - |
| Minority | 1.01 | (0.76, 1.34) |  | 1.11 | 1.00 |

| Table S16a  *Nationally Representative Descriptive Statistics for South Africa (N = 2,651)* | |
| --- | --- |
| Characteristic | *n* (%) |
| **Relationship with mother when growing up** |  |
| Very good | 2,186 (82%) |
| Somewhat good | 263 (9.9%) |
| Somewhat bad | 51 (1.9%) |
| Very bad | 39 (1.5%) |
| Does not apply | 90 (3.4%) |
| Missing | 21 (0.8%) |
| **Relationship with father when growing up** |  |
| Very good | 1,656 (62%) |
| Somewhat good | 333 (13%) |
| Somewhat bad | 86 (3.3%) |
| Very bad | 159 (6.0%) |
| Does not apply | 331 (12%) |
| Missing | 85 (3.2%) |
| **Family structure around age 12** |  |
| Parents were married | 1,321 (50%) |
| Parents were divorced | 131 (5.0%) |
| Parents were never married | 904 (34%) |
| One or both parents had died | 140 (5.3%) |
| Missing | 155 (5.8%) |
| **Subjective financial status of family around age 12** |  |
| Lived comfortably | 1,050 (40%) |
| Got by | 875 (33%) |
| Found it difficult | 432 (16%) |
| Found it very difficult | 289 (11%) |
| Missing | 5 (0.2%) |
| **Experienced abuse when growing up** |  |
| Yes | 450 (17%) |
| No | 2,149 (81%) |
| Missing | 52 (2.0%) |
| **Felt like an outsider in the family when growing up** |  |
| Yes | 434 (16%) |
| No | 2,211 (83%) |
| Missing | 6 (0.2%) |
| **Self-rated health when growing up** |  |
| Excellent | 1,225 (46%) |
| Very good | 590 (22%) |
| Good | 370 (14%) |
| Fair | 266 (10%) |
| Poor | 183 (6.9%) |
| Missing | 17 (0.6%) |
| **Immigration status** |  |
| Born in this country | 2,511 (95%) |
| Born in another country | 139 (5.2%) |
| Missing | 1 (< 0.1%) |
| **Frequency of religious service attendance around age 12** |  |
| ≥ 1/week | 1,681 (63%) |
| 1-3/month | 552 (21%) |
| < 1/month | 175 (6.6%) |
| Never | 217 (8.2%) |
| Missing | 26 (1.0%) |
| **Year of birth** |  |
| 1998-2005 (current age: 18-24 years) | 461 (17%) |
| 1993-1998 (current age: 25-29 years) | 364 (14%) |
| 1983-1993 (current age: 30-39 years) | 655 (25%) |
| 1973-1983 (current age: 40-49 years) | 522 (20%) |
| 1963-1973 (current age: 50-59 years) | 309 (12%) |
| 1953-1963 (current age: 60-69 years) | 195 (7.4%) |
| 1943-1953 (current age: 70-79 years) | 120 (4.5%) |
| 1943 or earlier (current age: 80+ years) | 17 (0.6%) |
| Missing | 9 (0.3%) |
| **Gender** |  |
| Male | 1,288 (49%) |
| Female | 1,356 (51%) |
| Other | 2 (< 0.1%) |
| Missing | 4 (0.2%) |
| **Religious affiliation at age 12** |  |
| Buddhism | 11 (0.4%) |
| Christianity | 2,323 (88%) |
| Hinduism | 2 (< 0.1%) |
| Islam | 52 (2.0%) |
| No religion/atheist/agnostic | 107 (4.1%) |
| Primal, animist, or folk religion | 117 (4.4%) |
| Shinto | 2 (< 0.1%) |
| Some other religion | 7 (0.3%) |
| Taoism | 1 (< 0.1%) |
| Missing | 27 (1.0%) |
| **Racial/ethnic identity** |  |
| Asian/Indian | 6 (0.2%) |
| Black | 2,381 (90%) |
| Colored | 252 (9.5%) |
| Other | 1 (< 0.1%) |
| White | 8 (0.3%) |
| Missing | 3 (0.1%) |

| Table S16b  *Regression of Dispositional Forgivingness on Candidate Predictors for South Africa* | | | | | |
| --- | --- | --- | --- | --- | --- |
| Variable | RR (95% CI) | | Global  *p*-value | *E*-values | |
|  |  |  |  | *E*-value for estimate | *E*-value for estimate |
| **Relationship with mother when growing up** |  |  | 1.000 |  |  |
| Ref: Very bad/somewhat bad | - | |  | - | - |
| Very good/somewhat good | 1.00 | (0.88, 1.13) |  | 1.07 | 1.00 |
| **Relationship with father when growing up** |  |  | 1.000 |  |  |
| Ref: Very bad/somewhat bad | - | |  | - | - |
| Very good/somewhat good | 1.00 | (0.93, 1.08) |  | 1.04 | 1.00 |
| **Family structure around age 12** |  |  | 0.002 |  |  |
| Ref: Parents were married | - | |  | - | - |
| Parents were divorced | 0.99 | (0.89, 1.11) |  | 1.09 | 1.00 |
| Parents were never married | 1.04 | (0.99, 1.09) |  | 1.23 | 1.00 |
| One or both parents had died | 0.85 | (0.73, 0.98) |  | 1.63 | 1.14 |
| **Subjective financial status of family around age 12** |  |  | 0.952 |  |  |
| Ref: Got by | - | |  | - | - |
| Lived comfortably | 1.00 | (0.95, 1.04) |  | 1.06 | 1.00 |
| Found it difficult | 1.02 | (0.96, 1.08) |  | 1.16 | 1.00 |
| Found it very difficult | 0.97 | (0.89, 1.05) |  | 1.21 | 1.00 |
| **Experienced abuse when growing up** |  |  | 0.004 |  |  |
| Ref: No | - | |  | - | - |
| Yes | 0.95 | (0.89, 1.01) |  | 1.30 | 1.00 |
| **Felt like an outsider in the family when growing up** |  |  | 0.981 |  |  |
| Ref: No | - | |  | - | - |
| Yes | 0.99 | (0.92, 1.06) |  | 1.12 | 1.00 |
| **Self-rated health when growing up** |  |  | 0.645 |  |  |
| Ref: Good | - | |  | - | - |
| Excellent | 0.98 | (0.92, 1.03) |  | 1.18 | 1.00 |
| Very good | 1.00 | (0.94, 1.07) |  | 1.05 | 1.00 |
| Fair | 0.98 | (0.90, 1.08) |  | 1.15 | 1.00 |
| Poor | 1.03 | (0.96, 1.11) |  | 1.22 | 1.00 |
| **Immigration status** |  |  | 0.057 |  |  |
| Ref: Born in this country | - | |  | - | - |
| Born in another country | 1.06 | (0.98, 1.15) |  | 1.31 | 1.00 |
| **Frequency of religious service attendance around age 12** |  |  | < 0.001 |  |  |
| Ref: Never | - | |  | - | - |
| ≥ 1/week | 1.09 | (0.97, 1.24) |  | 1.43 | 1.00 |
| 1-3/month | 1.03 | (0.91, 1.17) |  | 1.22 | 1.00 |
| < 1/month | 0.95 | (0.82, 1.12) |  | 1.27 | 1.00 |
| **Year of birth** |  |  | < 0.001 |  |  |
| Ref: 1998-2005 (current age: 18-24 years) | - | |  | - | - |
| 1993-1998 (current age: 25-29 years) | 0.98 | (0.91, 1.06) |  | 1.15 | 1.00 |
| 1983-1993 (current age: 30-39 years) | 1.03 | (0.97, 1.11) |  | 1.22 | 1.00 |
| 1973-1983 (current age: 40-49 years) | 1.00 | (0.93, 1.09) |  | 1.07 | 1.00 |
| 1963-1973 (current age: 50-59 years) | 1.08 | (1.00, 1.18) |  | 1.39 | 1.00 |
| 1953-1963 (current age: 60-69 years) | 1.05 | (0.95, 1.16) |  | 1.27 | 1.00 |
| 1943-1953 (current age: 70-79 years) | 1.15 | (1.03, 1.29) |  | 1.57 | 1.22 |
| 1943 or earlier (current age: 80+ years) | 1.21 | (1.11, 1.32) |  | 1.71 | 1.45 |
| **Gender** |  |  | < 0.001 |  |  |
| Ref: Male | - | |  | - | - |
| Female | 1.00 | (0.96, 1.04) |  | 1.06 | 1.00 |
| Other | 1.20 | (1.11, 1.31) |  | 1.69 | 1.45 |
| **Religious affiliation at age 12** |  |  | 0.996 |  |  |
| Ref: No religion/atheist/agnostic | - | |  | - | - |
| Christianity | 1.03 | (0.87, 1.21) |  | 1.19 | 1.00 |
| Primal, animist, or folk religion | 1.06 | (0.89, 1.28) |  | 1.33 | 1.00 |
| Some other religion | 1.03 | (0.84, 1.27) |  | 1.22 | 1.00 |
| **Racial/ethnic identity** |  |  | 0.413 |  |  |
| Ref: Majority | - | |  | - | - |
| Minority | 0.96 | (0.87, 1.05) |  | 1.26 | 1.00 |

| Table S17a  *Nationally Representative Descriptive Statistics for Spain (N = 6,290)* | |
| --- | --- |
| Characteristic | *n* (%) |
| **Relationship with mother when growing up** |  |
| Very good | 4,557 (72%) |
| Somewhat good | 1,258 (20%) |
| Somewhat bad | 248 (3.9%) |
| Very bad | 92 (1.5%) |
| Does not apply | 107 (1.7%) |
| Missing | 28 (0.4%) |
| **Relationship with father when growing up** |  |
| Very good | 4,131 (66%) |
| Somewhat good | 1,397 (22%) |
| Somewhat bad | 309 (4.9%) |
| Very bad | 178 (2.8%) |
| Does not apply | 243 (3.9%) |
| Missing | 33 (0.5%) |
| **Family structure around age 12** |  |
| Parents were married | 5,285 (84%) |
| Parents were divorced | 378 (6.0%) |
| Parents were never married | 312 (5.0%) |
| One or both parents had died | 126 (2.0%) |
| Missing | 188 (3.0%) |
| **Subjective financial status of family around age 12** |  |
| Lived comfortably | 2,041 (32%) |
| Got by | 2,956 (47%) |
| Found it difficult | 1,154 (18%) |
| Found it very difficult | 110 (1.7%) |
| Missing | 29 (0.5%) |
| **Experienced abuse when growing up** |  |
| Yes | 659 (10%) |
| No | 5,510 (88%) |
| Missing | 122 (1.9%) |
| **Felt like an outsider in the family when growing up** |  |
| Yes | 579 (9.2%) |
| No | 5,637 (90%) |
| Missing | 75 (1.2%) |
| **Self-rated health when growing up** |  |
| Excellent | 2,450 (39%) |
| Very good | 2,286 (36%) |
| Good | 1,235 (20%) |
| Fair | 164 (2.6%) |
| Poor | 135 (2.1%) |
| Missing | 20 (0.3%) |
| **Immigration status** |  |
| Born in this country | 5,479 (87%) |
| Born in another country | 788 (13%) |
| Missing | 23 (0.4%) |
| **Frequency of religious service attendance around age 12** |  |
| ≥ 1/week | 2,391 (38%) |
| 1-3/month | 1,132 (18%) |
| < 1/month | 1,287 (20%) |
| Never | 1,445 (23%) |
| Missing | 36 (0.6%) |
| **Year of birth** |  |
| 1998-2005 (current age: 18-24 years) | 594 (9.4%) |
| 1993-1998 (current age: 25-29 years) | 450 (7.2%) |
| 1983-1993 (current age: 30-39 years) | 1,111 (18%) |
| 1973-1983 (current age: 40-49 years) | 1,396 (22%) |
| 1963-1973 (current age: 50-59 years) | 1,252 (20%) |
| 1953-1963 (current age: 60-69 years) | 977 (16%) |
| 1943-1953 (current age: 70-79 years) | 467 (7.4%) |
| 1943 or earlier (current age: 80+ years) | 43 (0.7%) |
| Missing | 0 (0%) |
| **Gender** |  |
| Male | 3,142 (50%) |
| Female | 3,119 (50%) |
| Other | 6 (0.1%) |
| Missing | 22 (0.4%) |
| **Religious affiliation at age 12** |  |
| Buddhism | 8 (0.1%) |
| Christianity | 5,119 (81%) |
| Confucianism | 1 (< 0.1%) |
| Hinduism | 5 (< 0.1%) |
| Islam | 132 (2.1%) |
| Judaism | 5 (< 0.1%) |
| No religion/atheist/agnostic | 972 (15%) |
| Primal, animist, or folk religion | 4 (< 0.1%) |
| Sikhism | 2 (< 0.1%) |
| Some other religion | 13 (0.2%) |
| Missing | 29 (0.5%) |
| **Racial/ethnic identity** |  |
| Missing | 6,290 (100%) |

| Table S17b  *Regression of Dispositional Forgivingness on Candidate Predictors for Spain* | | | | | |
| --- | --- | --- | --- | --- | --- |
| Variable | RR (95% CI) | | Global  *p*-value | *E*-values | |
|  |  |  |  | *E*-value for estimate | *E*-value for estimate |
| **Relationship with mother when growing up** |  |  | < 0.001 |  |  |
| Ref: Very bad/somewhat bad | - | |  | - | - |
| Very good/somewhat good | 1.06 | (0.98, 1.16) |  | 1.32 | 1.00 |
| **Relationship with father when growing up** |  |  | 0.007 |  |  |
| Ref: Very bad/somewhat bad | - | |  | - | - |
| Very good/somewhat good | 0.96 | (0.90, 1.03) |  | 1.23 | 1.00 |
| **Family structure around age 12** |  |  | 0.068 |  |  |
| Ref: Parents were married | - | |  | - | - |
| Parents were divorced | 1.02 | (0.95, 1.09) |  | 1.15 | 1.00 |
| Parents were never married | 0.94 | (0.85, 1.03) |  | 1.33 | 1.00 |
| One or both parents had died | 0.89 | (0.75, 1.04) |  | 1.51 | 1.00 |
| **Subjective financial status of family around age 12** |  |  | 0.422 |  |  |
| Ref: Got by | - | |  | - | - |
| Lived comfortably | 1.03 | (1.00, 1.08) |  | 1.22 | 1.00 |
| Found it difficult | 1.00 | (0.95, 1.06) |  | 1.05 | 1.00 |
| Found it very difficult | 1.02 | (0.90, 1.17) |  | 1.18 | 1.00 |
| **Experienced abuse when growing up** |  |  | 0.935 |  |  |
| Ref: No | - | |  | - | - |
| Yes | 0.99 | (0.93, 1.05) |  | 1.13 | 1.00 |
| **Felt like an outsider in the family when growing up** |  |  | 0.956 |  |  |
| Ref: No |  | |  |  |  |
| Yes | 1.01 | (0.95, 1.07) |  | 1.12 | 1.00 |
| **Self-rated health when growing up** |  |  | 0.945 |  |  |
| Ref: Good | - | |  | - | - |
| Excellent | 1.03 | (0.98, 1.09) |  | 1.23 | 1.00 |
| Very good | 1.03 | (0.98, 1.09) |  | 1.22 | 1.00 |
| Fair | 1.07 | (0.95, 1.20) |  | 1.34 | 1.00 |
| Poor | 1.02 | (0.89, 1.16) |  | 1.16 | 1.00 |
| **Immigration status** |  |  | 0.456 |  |  |
| Ref: Born in this country | - | |  | - | - |
| Born in another country | 0.98 | (0.93, 1.03) |  | 1.18 | 1.00 |
| **Frequency of religious service attendance around age 12** |  |  | < 0.001 |  |  |
| Ref: Never | - | |  | - | - |
| ≥ 1/week | 1.16 | (1.10, 1.23) |  | 1.59 | 1.43 |
| 1-3/month | 1.15 | (1.09, 1.22) |  | 1.57 | 1.39 |
| < 1/month | 1.05 | (0.99, 1.12) |  | 1.29 | 1.00 |
| **Year of birth** |  |  | 1.000 |  |  |
| Ref: 1998-2005 (current age: 18-24 years) | - | |  | - | - |
| 1993-1998 (current age: 25-29 years) | 1.03 | (0.95, 1.13) |  | 1.22 | 1.00 |
| 1983-1993 (current age: 30-39 years) | 1.03 | (0.97, 1.11) |  | 1.22 | 1.00 |
| 1973-1983 (current age: 40-49 years) | 1.03 | (0.97, 1.11) |  | 1.23 | 1.00 |
| 1963-1973 (current age: 50-59 years) | 1.05 | (0.98, 1.13) |  | 1.28 | 1.00 |
| 1953-1963 (current age: 60-69 years) | 1.03 | (0.95, 1.12) |  | 1.22 | 1.00 |
| 1943-1953 (current age: 70-79 years) | 1.00 | (0.90, 1.11) |  | 1.07 | 1.00 |
| 1943 or earlier (current age: 80+ years) | 1.08 | (0.88, 1.32) |  | 1.37 | 1.00 |
| **Gender** |  |  | 0.833 |  |  |
| Ref: Male | - | |  | - | - |
| Female | 1.01 | (0.98, 1.05) |  | 1.12 | 1.00 |
| Other | 0.73 | (0.35, 1.52) |  | 2.07 | 1.00 |
| **Religious affiliation at age 12** |  |  | < 0.001 |  |  |
| Ref: No religion/atheist/agnostic | - | |  | - | - |
| Christianity | 1.07 | (1.01, 1.13) |  | 1.35 | 1.13 |
| Some other religion | 0.91 | (0.78, 1.07) |  | 1.42 | 1.00 |

| Table S18a  *Nationally Representative Descriptive Statistics for Sweden (N = 15,068)* | |
| --- | --- |
| Characteristic | *n* (%) |
| **Relationship with mother when growing up** |  |
| Very good | 8,743 (58%) |
| Somewhat good | 4,513 (30%) |
| Somewhat bad | 1,194 (7.9%) |
| Very bad | 371 (2.5%) |
| Does not apply | 216 (1.4%) |
| Missing | 30 (0.2%) |
| **Relationship with father when growing up** |  |
| Very good | 7,134 (47%) |
| Somewhat good | 4,885 (32%) |
| Somewhat bad | 1,588 (11%) |
| Very bad | 725 (4.8%) |
| Does not apply | 720 (4.8%) |
| Missing | 16 (0.1%) |
| **Family structure around age 12** |  |
| Parents were married | 10,887 (72%) |
| Parents were divorced | 1,927 (13%) |
| Parents were never married | 1,747 (12%) |
| One or both parents had died | 362 (2.4%) |
| Missing | 145 (1.0%) |
| **Subjective financial status of family around age 12** |  |
| Lived comfortably | 5,951 (39%) |
| Got by | 7,717 (51%) |
| Found it difficult | 1,238 (8.2%) |
| Found it very difficult | 140 (0.9%) |
| Missing | 22 (0.1%) |
| **Experienced abuse when growing up** |  |
| Yes | 2,288 (15%) |
| No | 12,735 (85%) |
| Missing | 45 (0.3%) |
| **Felt like an outsider in the family when growing up** |  |
| Yes | 1,867 (12%) |
| No | 13,034 (86%) |
| Missing | 168 (1.1%) |
| **Self-rated health when growing up** |  |
| Excellent | 5,733 (38%) |
| Very good | 5,124 (34%) |
| Good | 2,669 (18%) |
| Fair | 1,108 (7.4%) |
| Poor | 397 (2.6%) |
| Missing | 38 (0.2%) |
| **Immigration status** |  |
| Born in this country | 13,922 (92%) |
| Born in another country | 1,052 (7.0%) |
| Missing | 94 (0.6%) |
| **Frequency of religious service attendance around age 12** |  |
| ≥ 1/week | 955 (6.3%) |
| 1-3/month | 1,362 (9.0%) |
| < 1/month | 6,224 (41%) |
| Never | 6,472 (43%) |
| Missing | 54 (0.4%) |
| **Year of birth** |  |
| 1998-2005 (current age: 18-24 years) | 1,515 (10%) |
| 1993-1998 (current age: 25-29 years) | 1,399 (9.3%) |
| 1983-1993 (current age: 30-39 years) | 2,398 (16%) |
| 1973-1983 (current age: 40-49 years) | 2,221 (15%) |
| 1963-1973 (current age: 50-59 years) | 2,493 (17%) |
| 1953-1963 (current age: 60-69 years) | 2,168 (14%) |
| 1943-1953 (current age: 70-79 years) | 2,253 (15%) |
| 1943 or earlier (current age: 80+ years) | 621 (4.1%) |
| Missing | 0 (0%) |
| **Gender** |  |
| Male | 7,536 (50%) |
| Female | 7,493 (50%) |
| Other | 27 (0.2%) |
| Missing | 12 (< 0.1%) |
| **Religious affiliation at age 12** |  |
| Baha’i | 3 (< 0.1%) |
| Buddhism | 41 (0.3%) |
| Christianity | 10,617 (70%) |
| Confucianism | 4 (< 0.1%) |
| Hinduism | 16 (0.1%) |
| Islam | 462 (3.1%) |
| Judaism | 51 (0.3%) |
| No religion/atheist/agnostic | 3,738 (25%) |
| Primal, animist, or folk religion | 31 (0.2%) |
| Shinto | 1 (< 0.1%) |
| Sikhism | 9 (< 0.1%) |
| Some other religion | 69 (0.5%) |
| Missing | 26 (0.2%) |
| **Racial/ethnic identity** |  |
| Missing | 15,068 (100%) |

| Table S18b  *Regression of Dispositional Forgivingness on Candidate Predictors for Sweden* | | | | | |
| --- | --- | --- | --- | --- | --- |
| Variable | RR (95% CI) | | Global  *p*-value | *E*-values | |
|  |  |  |  | *E*-value for estimate | *E*-value for estimate |
| **Relationship with mother when growing up** |  |  | < 0.001 |  |  |
| Ref: Very bad/somewhat bad | - | |  | - | - |
| Very good/somewhat good | 1.09 | (1.04, 1.14) |  | 1.40 | 1.24 |
| **Relationship with father when growing up** |  |  | < 0.001 |  |  |
| Ref: Very bad/somewhat bad | - | |  | - | - |
| Very good/somewhat good | 1.06 | (1.02, 1.10) |  | 1.32 | 1.17 |
| **Family structure around age 12** |  |  | 0.832 |  |  |
| Ref: Parents were married | - | |  | - | - |
| Parents were divorced | 1.01 | (0.97, 1.04) |  | 1.09 | 1.00 |
| Parents were never married | 1.01 | (0.97, 1.05) |  | 1.11 | 1.00 |
| One or both parents had died | 1.05 | (0.98, 1.14) |  | 1.30 | 1.00 |
| **Subjective financial status of family around age 12** |  |  | 0.257 |  |  |
| Ref: Got by | - | |  | - | - |
| Lived comfortably | 1.02 | (1.00, 1.04) |  | 1.17 | 1.00 |
| Found it difficult | 1.00 | (0.95, 1.05) |  | 1.04 | 1.00 |
| Found it very difficult | 0.95 | (0.83, 1.09) |  | 1.28 | 1.00 |
| **Experienced abuse when growing up** |  |  | < 0.001 |  |  |
| Ref: No | - | |  | - | - |
| Yes | 1.04 | (1.00, 1.07) |  | 1.23 | 1.05 |
| **Felt like an outsider in the family when growing up** |  |  | 0.075 |  |  |
| Ref: No | - | |  | - | - |
| Yes | 0.97 | (0.93, 1.01) |  | 1.20 | 1.00 |
| **Self-rated health when growing up** |  |  | < 0.001 |  |  |
| Ref: Good | - | |  | - | - |
| Excellent | 1.09 | (1.06, 1.14) |  | 1.43 | 1.31 |
| Very good | 1.06 | (1.03, 1.10) |  | 1.32 | 1.19 |
| Fair | 1.07 | (1.02, 1.12) |  | 1.34 | 1.15 |
| Poor | 1.00 | (0.92, 1.09) |  | 1.06 | 1.00 |
| **Immigration status** |  |  | 0.125 |  |  |
| Ref: Born in this country | - | |  | - | - |
| Born in another country | 0.97 | (0.92, 1.02) |  | 1.21 | 1.00 |
| **Frequency of religious service attendance around age 12** |  |  | < 0.001 |  |  |
| Ref: Never | - | |  | - | - |
| ≥ 1/week | 1.14 | (1.10, 1.19) |  | 1.54 | 1.42 |
| 1-3/month | 1.07 | (1.03, 1.11) |  | 1.34 | 1.20 |
| < 1/month | 1.04 | (1.02, 1.07) |  | 1.25 | 1.14 |
| **Year of birth** |  |  | < 0.001 |  |  |
| Ref: 1998-2005 (current age: 18-24 years) | - | |  | - | - |
| 1993-1998 (current age: 25-29 years) | 1.01 | (0.96, 1.06) |  | 1.08 | 1.00 |
| 1983-1993 (current age: 30-39 years) | 1.04 | (0.99, 1.08) |  | 1.23 | 1.00 |
| 1973-1983 (current age: 40-49 years) | 1.01 | (0.97, 1.06) |  | 1.12 | 1.00 |
| 1963-1973 (current age: 50-59 years) | 1.03 | (0.99, 1.08) |  | 1.21 | 1.00 |
| 1953-1963 (current age: 60-69 years) | 0.97 | (0.92, 1.01) |  | 1.23 | 1.00 |
| 1943-1953 (current age: 70-79 years) | 1.01 | (0.96, 1.06) |  | 1.13 | 1.00 |
| 1943 or earlier (current age: 80+ years) | 1.06 | (1.00, 1.13) |  | 1.32 | 1.06 |
| **Gender** |  |  | 0.069 |  |  |
| Ref: Male | - | |  | - | - |
| Female | 1.02 | (1.00, 1.04) |  | 1.17 | 1.00 |
| Other | 1.02 | (0.76, 1.37) |  | 1.15 | 1.00 |
| **Religious affiliation at age 12** |  |  | < 0.001 |  |  |
| Ref: No religion/atheist/agnostic | - | |  | - | - |
| Christianity | 1.05 | (1.02, 1.08) |  | 1.27 | 1.15 |
| Some other religion | 0.91 | (0.84, 0.99) |  | 1.43 | 1.11 |

| Table S19a  *Nationally Representative Descriptive Statistics for Tanzania (N = 9,075)* | |
| --- | --- |
| Characteristic | *n* (%) |
| **Relationship with mother when growing up** |  |
| Very good | 7,739 (85%) |
| Somewhat good | 796 (8.8%) |
| Somewhat bad | 84 (0.9%) |
| Very bad | 84 (0.9%) |
| Does not apply | 303 (3.3%) |
| Missing | 70 (0.8%) |
| **Relationship with father when growing up** |  |
| Very good | 6,831 (75%) |
| Somewhat good | 1,101 (12%) |
| Somewhat bad | 203 (2.2%) |
| Very bad | 247 (2.7%) |
| Does not apply | 550 (6.1%) |
| Missing | 142 (1.6%) |
| **Family structure around age 12** |  |
| Parents were married | 6,929 (76%) |
| Parents were divorced | 678 (7.5%) |
| Parents were never married | 751 (8.3%) |
| One or both parents had died | 313 (3.4%) |
| Missing | 404 (4.4%) |
| **Subjective financial status of family around age 12** |  |
| Lived comfortably | 2,611 (29%) |
| Got by | 2,909 (32%) |
| Found it difficult | 2,679 (30%) |
| Found it very difficult | 814 (9.0%) |
| Missing | 61 (0.7%) |
| **Experienced abuse when growing up** |  |
| Yes | 716 (7.9%) |
| No | 8,328 (92%) |
| Missing | 32 (0.3%) |
| **Felt like an outsider in the family when growing up** |  |
| Yes | 734 (8.1%) |
| No | 8,320 (92%) |
| Missing | 22 (0.2%) |
| **Self-rated health when growing up** |  |
| Excellent | 2,406 (27%) |
| Very good | 2,036 (22%) |
| Good | 2,946 (32%) |
| Fair | 1,177 (13%) |
| Poor | 456 (5.0%) |
| Missing | 54 (0.6%) |
| **Immigration status** |  |
| Born in this country | 9,048 (100%) |
| Born in another country | 25 (0.3%) |
| Missing | 1 (< 0.1%) |
| **Frequency of religious service attendance around age 12** |  |
| ≥ 1/week | 5,580 (61%) |
| 1-3/month | 2,383 (26%) |
| < 1/month | 333 (3.7%) |
| Never | 595 (6.6%) |
| Missing | 184 (2.0%) |
| **Year of birth** |  |
| 1998-2005 (current age: 18-24 years) | 2,284 (25%) |
| 1993-1998 (current age: 25-29 years) | 1,349 (15%) |
| 1983-1993 (current age: 30-39 years) | 2,060 (23%) |
| 1973-1983 (current age: 40-49 years) | 1,503 (17%) |
| 1963-1973 (current age: 50-59 years) | 912 (10%) |
| 1953-1963 (current age: 60-69 years) | 575 (6.3%) |
| 1943-1953 (current age: 70-79 years) | 297 (3.3%) |
| 1943 or earlier (current age: 80+ years) | 93 (1.0%) |
| Missing | 2 (< 0.1%) |
| **Gender** |  |
| Male | 4,299 (47%) |
| Female | 4,776 (53%) |
| Other | 0 (0%) |
| Missing | 0 (0%) |
| **Religious affiliation at age 12** |  |
| Baha’i | 1 (< 0.1%) |
| Christianity | 5,651 (62%) |
| Islam | 3,060 (34%) |
| No religion/atheist/agnostic | 345 (3.8%) |
| Primal, animist, or folk religion | 11 (0.1%) |
| Missing | 7 (< 0.1%) |
| **Racial/ethnic identity** |  |
| African | 9,060 (100%) |
| Arab | 11 (0.1%) |
| Indian | 3 (< 0.1%) |
| Missing | 2 (< 0.1%) |

| Table S19b  *Regression of Dispositional Forgivingness on Candidate Predictors for Tanzania* | | | | | |
| --- | --- | --- | --- | --- | --- |
| Variable | RR (95% CI) | | Global  *p*-value | *E*-values | |
|  |  |  |  | *E*-value for estimate | *E*-value for estimate |
| **Relationship with mother when growing up** |  |  | 0.853 |  |  |
| Ref: Very bad/somewhat bad | - | |  | - | - |
| Very good/somewhat good | 1.01 | (0.94, 1.09) |  | 1.12 | 1.00 |
| **Relationship with father when growing up** |  |  | 0.061 |  |  |
| Ref: Very bad/somewhat bad | - | |  | - | - |
| Very good/somewhat good | 1.02 | (0.97, 1.07) |  | 1.17 | 1.00 |
| **Family structure around age 12** |  |  | 0.008 |  |  |
| Ref: Parents were married | - | |  | - | - |
| Parents were divorced | 0.99 | (0.95, 1.04) |  | 1.09 | 1.00 |
| Parents were never married | 0.94 | (0.90, 0.99) |  | 1.31 | 1.13 |
| One or both parents had died | 0.99 | (0.92, 1.06) |  | 1.12 | 1.00 |
| **Subjective financial status of family around age 12** |  |  | 0.945 |  |  |
| Ref: Got by | - | |  | - | - |
| Lived comfortably | 0.98 | (0.96, 1.01) |  | 1.15 | 1.00 |
| Found it difficult | 0.99 | (0.96, 1.02) |  | 1.11 | 1.00 |
| Found it very difficult | 0.99 | (0.94, 1.04) |  | 1.13 | 1.00 |
| **Experienced abuse when growing up** |  |  | < 0.001 |  |  |
| Ref: No | - | |  | - | - |
| Yes | 0.94 | (0.90, 0.99) |  | 1.32 | 1.12 |
| **Felt like an outsider in the family when growing up** |  |  | 0.841 |  |  |
| Ref: No | - | |  | - | - |
| Yes | 1.01 | (0.97, 1.06) |  | 1.12 | 1.00 |
| **Self-rated health when growing up** |  |  | 0.067 |  |  |
| Ref: Good | - | |  | - | - |
| Excellent | 1.00 | (0.97, 1.03) |  | 1.02 | 1.00 |
| Very good | 0.98 | (0.95, 1.00) |  | 1.18 | 1.00 |
| Fair | 0.98 | (0.95, 1.01) |  | 1.16 | 1.00 |
| Poor | 0.94 | (0.88, 1.01) |  | 1.32 | 1.00 |
| **Immigration status** |  |  | < 0.001 |  |  |
| Ref: Born in this country | - | |  | - | - |
| Born in another country | 1.14 | (1.05, 1.24) |  | 1.54 | 1.28 |
| **Frequency of religious service attendance around age 12** |  |  | 0.154 |  |  |
| Ref: Never | - | |  | - | - |
| ≥ 1/week | 1.04 | (0.98, 1.12) |  | 1.26 | 1.00 |
| 1-3/month | 1.03 | (0.96, 1.10) |  | 1.19 | 1.00 |
| < 1/month | 1.00 | (0.92, 1.08) |  | 1.05 | 1.00 |
| **Year of birth** |  |  | 1.000 |  |  |
| Ref: 1998-2005 (current age: 18-24 years) | - | |  | - | - |
| 1993-1998 (current age: 25-29 years) | 0.99 | (0.96, 1.03) |  | 1.09 | 1.00 |
| 1983-1993 (current age: 30-39 years) | 1.00 | (0.96, 1.03) |  | 1.07 | 1.00 |
| 1973-1983 (current age: 40-49 years) | 1.01 | (0.97, 1.04) |  | 1.08 | 1.00 |
| 1963-1973 (current age: 50-59 years) | 1.00 | (0.95, 1.04) |  | 1.07 | 1.00 |
| 1953-1963 (current age: 60-69 years) | 1.01 | (0.96, 1.06) |  | 1.10 | 1.00 |
| 1943-1953 (current age: 70-79 years) | 0.99 | (0.91, 1.08) |  | 1.12 | 1.00 |
| 1943 or earlier (current age: 80+ years) | 1.06 | (0.97, 1.16) |  | 1.32 | 1.00 |
| **Gender** |  |  | 0.039 |  |  |
| Ref: Male | - | |  | - | - |
| Female | 0.98 | (0.96, 1.01) |  | 1.15 | 1.00 |
| **Religious affiliation at age 12** |  |  | < 0.001 |  |  |
| Ref: No religion/atheist/agnostic | - | |  | - | - |
| Christianity | 0.95 | (0.88, 1.03) |  | 1.28 | 1.00 |
| Islam | 0.92 | (0.85, 1.00) |  | 1.39 | 1.07 |
| Some other religion | 1.13 | (1.05, 1.20) |  | 1.50 | 1.29 |
| **Racial/ethnic identity** |  |  | 0.994 |  |  |
| Ref: Majority | - | |  | - | - |
| Minority | 0.97 | (0.74, 1.26) |  | 1.23 | 1.00 |

| Table S20a  *Nationally Representative Descriptive Statistics for Türkiye (N = 1,473)* | |
| --- | --- |
| Characteristic | *n* (%) |
| **Relationship with mother when growing up** |  |
| Very good | 970 (66%) |
| Somewhat good | 401 (27%) |
| Somewhat bad | 48 (3.2%) |
| Very bad | 26 (1.8%) |
| Does not apply | 21 (1.4%) |
| Missing | 7 (0.5%) |
| **Relationship with father when growing up** |  |
| Very good | 795 (54%) |
| Somewhat good | 425 (29%) |
| Somewhat bad | 73 (5.0%) |
| Very bad | 95 (6.5%) |
| Does not apply | 60 (4.1%) |
| Missing | 25 (1.7%) |
| **Family structure around age 12** |  |
| Parents were married | 1,325 (90%) |
| Parents were divorced | 57 (3.9%) |
| Parents were never married | 7 (0.5%) |
| One or both parents had died | 61 (4.1%) |
| Missing | 23 (1.5%) |
| **Subjective financial status of family around age 12** |  |
| Lived comfortably | 498 (34%) |
| Got by | 647 (44%) |
| Found it difficult | 218 (15%) |
| Found it very difficult | 108 (7.3%) |
| Missing | 2 (0.1%) |
| **Experienced abuse when growing up** |  |
| Yes | 158 (11%) |
| No | 1,290 (88%) |
| Missing | 25 (1.7%) |
| **Felt like an outsider in the family when growing up** |  |
| Yes | 157 (11%) |
| No | 1,306 (89%) |
| Missing | 9 (0.6%) |
| **Self-rated health when growing up** |  |
| Excellent | 377 (26%) |
| Very good | 410 (28%) |
| Good | 419 (28%) |
| Fair | 220 (15%) |
| Poor | 47 (3.2%) |
| Missing | 0 (< 0.1%) |
| **Immigration status** |  |
| Born in this country | 1,415 (96%) |
| Born in another country | 58 (4.0%) |
| Missing | 0 (0%) |
| **Frequency of religious service attendance around age 12** |  |
| ≥ 1/week | 609 (41%) |
| 1-3/month | 238 (16%) |
| < 1/month | 225 (15%) |
| Never | 383 (26%) |
| Missing | 18 (1.2%) |
| **Year of birth** |  |
| 1998-2005 (current age: 18-24 years) | 222 (15%) |
| 1993-1998 (current age: 25-29 years) | 152 (10%) |
| 1983-1993 (current age: 30-39 years) | 315 (21%) |
| 1973-1983 (current age: 40-49 years) | 312 (21%) |
| 1963-1973 (current age: 50-59 years) | 225 (15%) |
| 1953-1963 (current age: 60-69 years) | 164 (11%) |
| 1943-1953 (current age: 70-79 years) | 65 (4.4%) |
| 1943 or earlier (current age: 80+ years) | 18 (1.2%) |
| Missing | 0 (0%) |
| **Gender** |  |
| Male | 754 (51%) |
| Female | 719 (49%) |
| Other | 0 (0%) |
| Missing | 0 (0%) |
| **Religious affiliation at age 12** |  |
| Christianity | 1 (< 0.1%) |
| Islam | 1,439 (98%) |
| Judaism | 1 (< 0.1%) |
| No religion/atheist/agnostic | 13 (0.9%) |
| Missing | 19 (1.3%) |
| **Racial/ethnic identity** |  |
| Albanian | 8 (0.5%) |
| Arab | 51 (3.5%) |
| Armenian | 1 (< 0.1%) |
| Azeri | 9 (0.6%) |
| Bosnian | 5 (0.3%) |
| Circassian | 19 (1.3%) |
| Georgian | 4 (0.3%) |
| Greek | 1 (< 0.1%) |
| Kurdish/Zaza | 252 (17%) |
| Laz | 25 (1.7%) |
| Other | 58 (3.9%) |
| Turkish | 1,030 (70%) |
| Uyghur | 1 (< 0.1%) |
| Missing | 9 (0.6%) |

| Table S20b  *Regression of Dispositional Forgivingness on Candidate Predictors for Türkiye* | | | | | |
| --- | --- | --- | --- | --- | --- |
| Variable | RR (95% CI) | | Global  *p*-value | *E*-values | |
|  |  |  |  | *E*-value for estimate | *E*-value for estimate |
| **Relationship with mother when growing up** |  |  | 0.095 |  |  |
| Ref: Very bad/somewhat bad | - | |  | - | - |
| Very good/somewhat good | 1.14 | (0.81, 1.58) |  | 1.53 | 1.00 |
| **Relationship with father when growing up** |  |  | 0.305 |  |  |
| Ref: Very bad/somewhat bad | - | |  | - | - |
| Very good/somewhat good | 1.07 | (0.85, 1.35) |  | 1.34 | 1.00 |
| **Family structure around age 12** |  |  | 0.002 |  |  |
| Ref: Parents were married | - | |  | - | - |
| Parents were divorced | 1.19 | (0.86, 1.65) |  | 1.67 | 1.00 |
| Parents were never married | 0.79 | (0.29, 2.22) |  | 1.83 | 1.00 |
| One or both parents had died | 1.46 | (1.07, 1.98) |  | 2.27 | 1.35 |
| **Subjective financial status of family around age 12** |  |  | 0.050 |  |  |
| Ref: Got by | - | |  | - | - |
| Lived comfortably | 0.95 | (0.80, 1.12) |  | 1.30 | 1.00 |
| Found it difficult | 1.12 | (0.91, 1.37) |  | 1.49 | 1.00 |
| Found it very difficult | 0.73 | (0.49, 1.08) |  | 2.08 | 1.00 |
| **Experienced abuse when growing up** |  |  | 0.949 |  |  |
| Ref: No | - | |  | - | - |
| Yes | 1.05 | (0.82, 1.35) |  | 1.29 | 1.00 |
| **Felt like an outsider in the family when growing up** |  |  | < 0.001 |  |  |
| Ref: No | - | |  | - | - |
| Yes | 1.31 | (1.06, 1.61) |  | 1.94 | 1.32 |
| **Self-rated health when growing up** |  |  | 0.140 |  |  |
| Ref: Good | - | |  | - | - |
| Excellent | 1.00 | (0.82, 1.23) |  | 1.01 | 1.00 |
| Very good | 1.13 | (0.94, 1.36) |  | 1.52 | 1.00 |
| Fair | 0.98 | (0.77, 1.24) |  | 1.17 | 1.00 |
| Poor | 0.61 | (0.32, 1.16) |  | 2.69 | 1.00 |
| **Immigration status** |  |  | 0.522 |  |  |
| Ref: Born in this country | - | |  | - | - |
| Born in another country | 0.82 | (0.51, 1.31) |  | 1.75 | 1.00 |
| **Frequency of religious service attendance around age 12** |  |  | 0.510 |  |  |
| Ref: Never | - | |  | - | - |
| ≥ 1/week | 1.19 | (0.97, 1.45) |  | 1.65 | 1.00 |
| 1-3/month | 1.14 | (0.90, 1.45) |  | 1.55 | 1.00 |
| < 1/month | 1.08 | (0.84, 1.40) |  | 1.37 | 1.00 |
| **Year of birth** |  |  | < 0.001 |  |  |
| Ref: 1998-2005 (current age: 18-24 years) | - | |  | - | - |
| 1993-1998 (current age: 25-29 years) | 0.93 | (0.68, 1.27) |  | 1.37 | 1.00 |
| 1983-1993 (current age: 30-39 years) | 0.96 | (0.75, 1.23) |  | 1.24 | 1.00 |
| 1973-1983 (current age: 40-49 years) | 1.26 | (1.00, 1.58) |  | 1.82 | 1.00 |
| 1963-1973 (current age: 50-59 years) | 1.20 | (0.91, 1.58) |  | 1.68 | 1.00 |
| 1953-1963 (current age: 60-69 years) | 1.51 | (1.13, 2.01) |  | 2.38 | 1.52 |
| 1943-1953 (current age: 70-79 years) | 1.86 | (1.30, 2.65) |  | 3.13 | 1.93 |
| 1943 or earlier (current age: 80+ years) | 1.97 | (1.18, 3.31) |  | 3.37 | 1.65 |
| **Gender** |  |  | 0.053 |  |  |
| Ref: Male | - | |  | - | - |
| Female | 0.90 | (0.76, 1.05) |  | 1.48 | 1.00 |
| **Religious affiliation at age 12** |  |  | 0.010 |  |  |
| Ref: Islam | - | |  | - | - |
| Some other religion | 0.48 | (0.18, 1.31) |  | 3.57 | 1.00 |
| **Racial/ethnic identity** |  |  | 0.937 |  |  |
| Ref: Majority | - | |  | - | - |
| Minority | 0.96 | (0.81, 1.14) |  | 1.24 | 1.00 |

| Table S21a  *Nationally Representative Descriptive Statistics for United Kingdom (N = 5,368)* | |
| --- | --- |
| Characteristic | *n* (%) |
| **Relationship with mother when growing up** |  |
| Very good | 3,435 (64%) |
| Somewhat good | 1,338 (25%) |
| Somewhat bad | 325 (6.1%) |
| Very bad | 150 (2.8%) |
| Does not apply | 92 (1.7%) |
| Missing | 27 (0.5%) |
| **Relationship with father when growing up** |  |
| Very good | 2,907 (54%) |
| Somewhat good | 1,383 (26%) |
| Somewhat bad | 407 (7.6%) |
| Very bad | 321 (6.0%) |
| Does not apply | 321 (6.0%) |
| Missing | 29 (0.5%) |
| **Family structure around age 12** |  |
| Parents were married | 4,343 (81%) |
| Parents were divorced | 481 (9.0%) |
| Parents were never married | 315 (5.9%) |
| One or both parents had died | 154 (2.9%) |
| Missing | 75 (1.4%) |
| **Subjective financial status of family around age 12** |  |
| Lived comfortably | 2,552 (48%) |
| Got by | 1,933 (36%) |
| Found it difficult | 632 (12%) |
| Found it very difficult | 230 (4.3%) |
| Missing | 22 (0.4%) |
| **Experienced abuse when growing up** |  |
| Yes | 864 (16%) |
| No | 4,455 (83%) |
| Missing | 49 (0.9%) |
| **Felt like an outsider in the family when growing up** |  |
| Yes | 1,017 (19%) |
| No | 4,308 (80%) |
| Missing | 43 (0.8%) |
| **Self-rated health when growing up** |  |
| Excellent | 2,154 (40%) |
| Very good | 1,736 (32%) |
| Good | 995 (19%) |
| Fair | 332 (6.2%) |
| Poor | 130 (2.4%) |
| Missing | 20 (0.4%) |
| **Immigration status** |  |
| Born in this country | 4,659 (87%) |
| Born in another country | 682 (13%) |
| Missing | 27 (0.5%) |
| **Frequency of religious service attendance around age 12** |  |
| ≥ 1/week | 1,732 (32%) |
| 1-3/month | 733 (14%) |
| < 1/month | 903 (17%) |
| Never | 1,972 (37%) |
| Missing | 28 (0.5%) |
| **Year of birth** |  |
| 1998-2005 (current age: 18-24 years) | 490 (9.1%) |
| 1993-1998 (current age: 25-29 years) | 391 (7.3%) |
| 1983-1993 (current age: 30-39 years) | 946 (18%) |
| 1973-1983 (current age: 40-49 years) | 827 (15%) |
| 1963-1973 (current age: 50-59 years) | 949 (18%) |
| 1953-1963 (current age: 60-69 years) | 889 (17%) |
| 1943-1953 (current age: 70-79 years) | 711 (13%) |
| 1943 or earlier (current age: 80+ years) | 163 (3.0%) |
| Missing | 1 (< 0.1%) |
| **Gender** |  |
| Male | 2,557 (48%) |
| Female | 2,789 (52%) |
| Other | 14 (0.3%) |
| Missing | 9 (0.2%) |
| **Religious affiliation at age 12** |  |
| Baha’i | 5 (< 0.1%) |
| Buddhism | 15 (0.3%) |
| Christianity | 3,461 (64%) |
| Confucianism | 3 (< 0.1%) |
| Hinduism | 88 (1.6%) |
| Islam | 230 (4.3%) |
| Jainism | 0 (< 0.1%) |
| Judaism | 59 (1.1%) |
| No religion/atheist/agnostic | 1,409 (26%) |
| Primal, animist, or folk religion | 22 (0.4%) |
| Sikhism | 30 (0.6%) |
| Some other religion | 24 (0.5%) |
| Taoism | 2 (< 0.1%) |
| Missing | 21 (0.4%) |
| **Racial/ethnic identity** |  |
| Asian | 426 (7.9%) |
| Black | 152 (2.8%) |
| Other | 96 (1.8%) |
| White | 4,647 (87%) |
| Missing | 47 (0.9%) |

| Table S21b  *Regression of Dispositional Forgivingness on Candidate Predictors for United Kingdom* | | | | | |
| --- | --- | --- | --- | --- | --- |
| Variable | RR (95% CI) | | Global  *p*-value | *E*-values | |
|  |  |  |  | *E*-value for estimate | *E*-value for estimate |
| **Relationship with mother when growing up** |  |  | 0.985 |  |  |
| Ref: Very bad/somewhat bad | - | |  | - | - |
| Very good/somewhat good | 0.99 | (0.92, 1.08) |  | 1.08 | 1.00 |
| **Relationship with father when growing up** |  |  | < 0.001 |  |  |
| Ref: Very bad/somewhat bad | - | |  | - | - |
| Very good/somewhat good | 1.07 | (0.99, 1.16) |  | 1.35 | 1.00 |
| **Family structure around age 12** |  |  | 0.107 |  |  |
| Ref: Parents were married | - | |  | - | - |
| Parents were divorced | 1.03 | (0.94, 1.12) |  | 1.20 | 1.00 |
| Parents were never married | 0.94 | (0.83, 1.07) |  | 1.31 | 1.00 |
| One or both parents had died | 1.12 | (0.98, 1.28) |  | 1.49 | 1.00 |
| **Subjective financial status of family around age 12** |  |  | 0.014 |  |  |
| Ref: Got by | - | |  | - | - |
| Lived comfortably | 1.02 | (0.97, 1.08) |  | 1.18 | 1.00 |
| Found it difficult | 0.92 | (0.84, 1.01) |  | 1.39 | 1.00 |
| Found it very difficult | 1.01 | (0.88, 1.15) |  | 1.09 | 1.00 |
| **Experienced abuse when growing up** |  |  | 0.747 |  |  |
| Ref: No | - | |  | - | - |
| Yes | 1.02 | (0.96, 1.09) |  | 1.17 | 1.00 |
| **Felt like an outsider in the family when growing up** |  |  | 0.080 |  |  |
| Ref: No | - | |  | - | - |
| Yes | 0.96 | (0.90, 1.02) |  | 1.26 | 1.00 |
| **Self-rated health when growing up** |  |  | 0.241 |  |  |
| Ref: Good | - | |  | - | - |
| Excellent | 1.00 | (0.93, 1.07) |  | 1.04 | 1.00 |
| Very good | 1.01 | (0.94, 1.08) |  | 1.09 | 1.00 |
| Fair | 0.87 | (0.76, 1.00) |  | 1.55 | 1.05 |
| Poor | 1.03 | (0.88, 1.21) |  | 1.21 | 1.00 |
| **Immigration status** |  |  | 0.947 |  |  |
| Ref: Born in this country | - | |  | - | - |
| Born in another country | 0.98 | (0.92, 1.06) |  | 1.14 | 1.00 |
| **Frequency of religious service attendance around age 12** |  |  | < 0.001 |  |  |
| Ref: Never | - | |  | - | - |
| ≥ 1/week | 1.16 | (1.09, 1.24) |  | 1.60 | 1.40 |
| 1-3/month | 1.20 | (1.11, 1.29) |  | 1.68 | 1.46 |
| < 1/month | 1.09 | (1.02, 1.18) |  | 1.41 | 1.15 |
| **Year of birth** |  |  | < 0.001 |  |  |
| Ref: 1998-2005 (current age: 18-24 years) | - | |  | - | - |
| 1993-1998 (current age: 25-29 years) | 1.01 | (0.89, 1.13) |  | 1.08 | 1.00 |
| 1983-1993 (current age: 30-39 years) | 1.02 | (0.92, 1.13) |  | 1.16 | 1.00 |
| 1973-1983 (current age: 40-49 years) | 0.93 | (0.83, 1.04) |  | 1.37 | 1.00 |
| 1963-1973 (current age: 50-59 years) | 0.94 | (0.84, 1.05) |  | 1.33 | 1.00 |
| 1953-1963 (current age: 60-69 years) | 0.93 | (0.83, 1.04) |  | 1.36 | 1.00 |
| 1943-1953 (current age: 70-79 years) | 0.87 | (0.77, 0.99) |  | 1.56 | 1.14 |
| 1943 or earlier (current age: 80+ years) | 0.98 | (0.85, 1.13) |  | 1.15 | 1.00 |
| **Gender** |  |  | 0.159 |  |  |
| Ref: Male | - | |  | - | - |
| Female | 1.04 | (0.99, 1.09) |  | 1.23 | 1.00 |
| Other | 0.73 | (0.30, 1.78) |  | 2.11 | 1.00 |
| **Religious affiliation at age 12** |  |  | 0.004 |  |  |
| Ref: No religion/atheist/agnostic | - | |  | - | - |
| Christianity | 1.07 | (1.00, 1.14) |  | 1.34 | 1.02 |
| Islam | 1.04 | (0.91, 1.20) |  | 1.26 | 1.00 |
| Some other religion | 0.94 | (0.81, 1.09) |  | 1.33 | 1.00 |
| **Racial/ethnic identity** |  |  | 0.803 |  |  |
| Ref: Majority | - | |  | - | - |
| Minority | 0.97 | (0.89, 1.06) |  | 1.20 | 1.00 |

| Table S22a  *Nationally Representative Descriptive Statistics for United States (N = 38,312)* | |
| --- | --- |
| Characteristic | *n* (%) |
| **Relationship with mother when growing up** |  |
| Very good | 20,590 (54%) |
| Somewhat good | 11,525 (30%) |
| Somewhat bad | 3,523 (9.2%) |
| Very bad | 1,874 (4.9%) |
| Does not apply | 694 (1.8%) |
| Missing | 106 (0.3%) |
| **Relationship with father when growing up** |  |
| Very good | 15,313 (40%) |
| Somewhat good | 12,665 (33%) |
| Somewhat bad | 4,879 (13%) |
| Very bad | 2,604 (6.8%) |
| Does not apply | 2,811 (7.3%) |
| Missing | 38 (0.1%) |
| **Family structure around age 12** |  |
| Parents were married | 27,415 (72%) |
| Parents were divorced | 6,325 (17%) |
| Parents were never married | 3,048 (8.0%) |
| One or both parents had died | 1,024 (2.7%) |
| Missing | 500 (1.3%) |
| **Subjective financial status of family around age 12** |  |
| Lived comfortably | 15,116 (39%) |
| Got by | 15,682 (41%) |
| Found it difficult | 5,152 (13%) |
| Found it very difficult | 2,342 (6.1%) |
| Missing | 19 (< 0.1%) |
| **Experienced abuse when growing up** |  |
| Yes | 10,026 (26%) |
| No | 28,045 (73%) |
| Missing | 242 (0.6%) |
| **Felt like an outsider in the family when growing up** |  |
| Yes | 10,185 (27%) |
| No | 27,714 (72%) |
| Missing | 413 (1.1%) |
| **Self-rated health when growing up** |  |
| Excellent | 16,866 (44%) |
| Very good | 12,108 (32%) |
| Good | 6,444 (17%) |
| Fair | 2,303 (6.0%) |
| Poor | 520 (1.4%) |
| Missing | 71 (0.2%) |
| **Immigration status** |  |
| Born in this country | 34,865 (91%) |
| Born in another country | 3,020 (7.9%) |
| Missing | 427 (1.1%) |
| **Frequency of religious service attendance around age 12** |  |
| ≥ 1/week | 18,609 (49%) |
| 1-3/month | 6,644 (17%) |
| < 1/month | 5,829 (15%) |
| Never | 7,085 (18%) |
| Missing | 145 (0.4%) |
| **Year of birth** |  |
| 1998-2005 (current age: 18-24 years) | 2,682 (7.0%) |
| 1993-1998 (current age: 25-29 years) | 3,540 (9.2%) |
| 1983-1993 (current age: 30-39 years) | 7,284 (19%) |
| 1973-1983 (current age: 40-49 years) | 5,649 (15%) |
| 1963-1973 (current age: 50-59 years) | 6,745 (18%) |
| 1953-1963 (current age: 60-69 years) | 6,832 (18%) |
| 1943-1953 (current age: 70-79 years) | 4,054 (11%) |
| 1943 or earlier (current age: 80+ years) | 1,525 (4.0%) |
| Missing | 0 (0%) |
| **Gender** |  |
| Male | 18,222 (48%) |
| Female | 19,562 (51%) |
| Other | 392 (1.0%) |
| Missing | 136 (0.4%) |
| **Religious affiliation at age 12** |  |
| Baha’i | 4 (< 0.1%) |
| Buddhism | 172 (0.4%) |
| Christianity | 30,444 (79%) |
| Confucianism | 8 (< 0.1%) |
| Hinduism | 203 (0.5%) |
| Islam | 220 (0.6%) |
| Jainism | 18 (< 0.1%) |
| Judaism | 787 (2.1%) |
| No religion/atheist/agnostic | 5,845 (15%) |
| Primal, animist, or folk religion | 67 (0.2%) |
| Shinto | 6 (< 0.1%) |
| Sikhism | 47 (0.1%) |
| Some other religion | 359 (0.9%) |
| Taoism | 17 (< 0.1%) |
| Missing | 115 (0.3%) |
| **Racial/ethnic identity** |  |
| Asian | 2,466 (6.4%) |
| Black | 4,501 (12%) |
| Hispanic | 6,724 (18%) |
| Other | 997 (2.6%) |
| White | 23,605 (62%) |
| Missing | 20 (< 0.1%) |

| Table S22b  *Regression of Dispositional Forgivingness on Candidate Predictors for United States* | | | | | |
| --- | --- | --- | --- | --- | --- |
| Variable | RR (95% CI) | | Global  *p*-value | *E*-values | |
|  |  |  |  | *E*-value for estimate | *E*-value for estimate |
| **Relationship with mother when growing up** |  |  | < 0.001 |  |  |
| Ref: Very bad/somewhat bad | - | |  | - | - |
| Very good/somewhat good | 1.08 | (1.02, 1.14) |  | 1.37 | 1.17 |
| **Relationship with father when growing up** |  |  | < 0.001 |  |  |
| Ref: Very bad/somewhat bad | - | |  | - | - |
| Very good/somewhat good | 1.05 | (1.00, 1.10) |  | 1.27 | 1.05 |
| **Family structure around age 12** |  |  | 0.057 |  |  |
| Ref: Parents were married | - | |  | - | - |
| Parents were divorced | 1.05 | (1.00, 1.09) |  | 1.27 | 1.07 |
| Parents were never married | 1.02 | (0.93, 1.12) |  | 1.17 | 1.00 |
| One or both parents had died | 1.03 | (0.94, 1.14) |  | 1.22 | 1.00 |
| **Subjective financial status of family around age 12** |  |  | 0.407 |  |  |
| Ref: Got by | - | |  | - | - |
| Lived comfortably | 1.03 | (1.00, 1.05) |  | 1.19 | 1.00 |
| Found it difficult | 1.02 | (0.97, 1.07) |  | 1.15 | 1.00 |
| Found it very difficult | 1.02 | (0.94, 1.10) |  | 1.14 | 1.00 |
| **Experienced abuse when growing up** |  |  | 1.000 |  |  |
| Ref: No | - | |  | - | - |
| Yes | 1.00 | (0.96, 1.04) |  | 1.03 | 1.00 |
| **Felt like an outsider in the family when growing up** |  |  | < 0.001 |  |  |
| Ref: No | - | |  | - | - |
| Yes | 0.95 | (0.91, 0.99) |  | 1.30 | 1.11 |
| **Self-rated health when growing up** |  |  | 0.003 |  |  |
| Ref: Good | - | |  | - | - |
| Excellent | 1.01 | (0.97, 1.06) |  | 1.14 | 1.00 |
| Very good | 0.98 | (0.94, 1.03) |  | 1.14 | 1.00 |
| Fair | 0.92 | (0.84, 1.02) |  | 1.37 | 1.00 |
| Poor | 0.90 | (0.74, 1.11) |  | 1.43 | 1.00 |
| **Immigration status** |  |  | < 0.001 |  |  |
| Ref: Born in this country | - | |  | - | - |
| Born in another country | 1.07 | (1.02, 1.14) |  | 1.36 | 1.16 |
| **Frequency of religious service attendance around age 12** |  |  | < 0.001 |  |  |
| Ref: Never | - | |  | - | - |
| ≥ 1/week | 1.15 | (1.10, 1.22) |  | 1.58 | 1.43 |
| 1-3/month | 1.08 | (1.03, 1.15) |  | 1.40 | 1.19 |
| < 1/month | 1.08 | (1.02, 1.15) |  | 1.39 | 1.18 |
| **Year of birth** |  |  | < 0.001 |  |  |
| Ref: 1998-2005 (current age: 18-24 years) | - | |  | - | - |
| 1993-1998 (current age: 25-29 years) | 1.03 | (0.91, 1.16) |  | 1.19 | 1.00 |
| 1983-1993 (current age: 30-39 years) | 1.05 | (0.95, 1.18) |  | 1.30 | 1.00 |
| 1973-1983 (current age: 40-49 years) | 1.06 | (0.95, 1.18) |  | 1.30 | 1.00 |
| 1963-1973 (current age: 50-59 years) | 1.08 | (0.97, 1.20) |  | 1.37 | 1.00 |
| 1953-1963 (current age: 60-69 years) | 1.12 | (1.01, 1.24) |  | 1.47 | 1.08 |
| 1943-1953 (current age: 70-79 years) | 1.11 | (0.99, 1.22) |  | 1.44 | 1.00 |
| 1943 or earlier (current age: 80+ years) | 1.14 | (1.02, 1.27) |  | 1.53 | 1.17 |
| **Gender** |  |  | < 0.001 |  |  |
| Ref: Male | - | |  | - | - |
| Female | 1.03 | (1.00, 1.06) |  | 1.20 | 1.04 |
| Other | 0.80 | (0.60, 1.08) |  | 1.79 | 1.00 |
| **Religious affiliation at age 12** |  |  | 0.020 |  |  |
| Ref: No religion/atheist/agnostic | - | |  | - | - |
| Christianity | 1.04 | (0.98, 1.11) |  | 1.25 | 1.00 |
| Judaism | 1.01 | (0.94, 1.09) |  | 1.11 | 1.00 |
| Some other religion | 0.93 | (0.82, 1.06) |  | 1.36 | 1.00 |
| **Racial/ethnic identity** |  |  | < 0.001 |  |  |
| Ref: Majority | - | |  | - | - |
| Minority | 0.94 | (0.91, 0.98) |  | 1.31 | 1.18 |

| Table S23  *Population Weighted Meta-Analyses for Associations of Candidate Predictors With Forgivingness in Adulthood* | | | |
| --- | --- | --- | --- |
| Variable | RR (95% CI) | *E*-values | |
|  |  | *E*-value for estimate | *E*-value for 95% CI |
| **Relationship with mother when growing up** |  |  |  |
| Ref: Very bad/somewhat bad | - | - | - |
| Very good/somewhat good | 1.03 (0.99, 1.08) | 1.21 | 1.00 |
| **Relationship with father when growing up** |  |  |  |
| Ref: Very bad/somewhat bad | - | - | - |
| Very good/somewhat good | 1.05 (1.01, 1.09) | 1.27 | 1.11 |
| **Family structure around age 12** |  |  |  |
| Ref: Parents were married | - | - | - |
| Parents were divorced | 1.00 (0.95, 1.06) | 1.07 | 1.00 |
| Parents were Parents were never married | 0.99 (0.96, 1.03) | 1.10 | 1.00 |
| One or both parents had died | 1.01 (0.98, 1.04) | 1.12 | 1.00 |
| **Subjective financial status of family around age 12** |  |  |  |
| Ref: Got by | - | - | - |
| Lived comfortably | 1.00 (0.99, 1.02) | 1.07 | 1.00 |
| Found it difficult | 1.00 (0.98, 1.02) | 1.04 | 1.00 |
| Found it very difficult | 0.98 (0.96, 1.01) | 1.15 | 1.00 |
| **Experienced abuse when growing up** |  |  |  |
| Ref: No | - | - | - |
| Yes | 0.97 (0.96, 0.99) | 1.19 | 1.08 |
| **Felt like an outsider in the family when growing up** |  |  |  |
| Ref: No | - | - | - |
| Yes | 1.01 (0.99, 1.02) | 1.08 | 1.00 |
| **Self-rated health when growing up** |  |  |  |
| Ref: Good | - | - | - |
| Excellent | 1.02 (1.00, 1.04) | 1.15 | 1.00 |
| Very good | 1.01 (1.00, 1.03) | 1.13 | 1.00 |
| Fair | 0.97 (0.95, 0.99) | 1.20 | 1.10 |
| Poor | 0.97 (0.93, 1.01) | 1.21 | 1.00 |
| **Immigration status** |  |  |  |
| Ref: Born in this country | - | - | - |
| Born in another country | 1.01 (0.95, 1.08) | 1.13 | 1.00 |
| **Frequency of religious service attendance around age 12** |  |  |  |
| Ref: Never | - | - | - |
| ≥ 1/week | 1.09 (1.06, 1.11) | 1.40 | 1.32 |
| 1-3/month | 1.06 (1.04, 1.08) | 1.31 | 1.23 |
| < 1/month | 1.04 (1.01, 1.06) | 1.23 | 1.11 |
| **Year of birth** |  |  |  |
| Ref: 1998-2005 (current age: 18-24 years) | - | - | - |
| 1993-1998 (current age: 25-29 years) | 1.03 (1.01, 1.06) | 1.21 | 1.08 |
| 1983-1993 (current age: 30-39 years) | 1.03 (1.01, 1.06) | 1.22 | 1.12 |
| 1973-1983 (current age: 40-49 years) | 1.05 (1.03, 1.08) | 1.29 | 1.21 |
| 1963-1973 (current age: 50-59 years) | 1.05 (1.02, 1.08) | 1.28 | 1.18 |
| 1953-1963 (current age: 60-69 years) | 1.09 (1.06, 1.12) | 1.39 | 1.30 |
| 1943-1953 (current age: 70-79 years) | 1.08 (1.04, 1.12) | 1.37 | 1.24 |
| 1943 or earlier (current age: 80+ years) | 1.13 (1.05, 1.22) | 1.52 | 1.29 |
| **Gender** |  |  |  |
| Ref: Male | - | - | - |
| Female | 1.02 (1.01, 1.03) | 1.16 | 1.09 |
| Other | 0.75 (0.61, 0.92) | 2.00 | 1.40 |

| Table S24  *Random Effects Meta-Analyses for Associations of Candidate Predictors With Forgivingness in Adulthood Using Alternative Coding of Forgivingness* | | | |
| --- | --- | --- | --- |
| Variable | RR (95% CI) | *E*-values | |
|  |  | *E*-value for estimate | *E*-value for 95% CI |
| **Relationship with mother when growing up** |  |  |  |
| Ref: Very bad/somewhat bad | - | - | - |
| Very good/somewhat good | 1.08 (1.01, 1.15) | 1.37 | 1.11 |
| **Relationship with father when growing up** |  |  |  |
| Ref: Very bad/somewhat bad | - | - | - |
| Very good/somewhat good | 1.05 (1.01, 1.09) | 1.28 | 1.13 |
| **Family structure around age 12** |  |  |  |
| Ref: Parents were married | - | - | - |
| Parents were divorced | 1.00 (0.95, 1.04) | 1.06 | 1.00 |
| Parents were Parents were never married | 1.02 (0.95, 1.09) | 1.15 | 1.00 |
| One or both parents had died | 1.01 (0.96, 1.06) | 1.09 | 1.00 |
| **Subjective financial status of family around age 12** |  |  |  |
| Ref: Got by | - | - | - |
| Lived comfortably | 1.05 (1.00, 1.09) | 1.27 | 1.03 |
| Found it difficult | 1.05 (1.03, 1.08) | 1.29 | 1.19 |
| Found it very difficult | 1.10 (1.03, 1.18) | 1.44 | 1.21 |
| **Experienced abuse when growing up** |  |  |  |
| Ref: No | - | - | - |
| Yes | 1.01 (0.96, 1.06) | 1.12 | 1.00 |
| **Felt like an outsider in the family when growing up** |  |  |  |
| Ref: No | - | - | - |
| Yes | 1.03 (0.96, 1.11) | 1.20 | 1.00 |
| **Self-rated health when growing up** |  |  |  |
| Ref: Good | - | - | - |
| Excellent | 1.18 (1.07, 1.31) | 1.65 | 1.34 |
| Very good | 1.02 (0.99, 1.06) | 1.18 | 1.00 |
| Fair | 1.03 (0.97, 1.08) | 1.19 | 1.00 |
| Poor | 1.08 (1.00, 1.16) | 1.37 | 1.03 |
| **Immigration status** |  |  |  |
| Ref: Born in this country | - | - | - |
| Born in another country | 1.01 (0.92, 1.10) | 1.10 | 1.00 |
| **Frequency of religious service attendance around age 12** |  |  |  |
| Ref: Never | - | - | - |
| ≥ 1/week | 1.29 (1.17, 1.42) | 1.90 | 1.61 |
| 1-3/month | 1.11 (1.03, 1.21) | 1.47 | 1.20 |
| < 1/month | 1.00 (0.95, 1.06) | 1.05 | 1.00 |
| **Year of birth** |  |  |  |
| Ref: 1998-2005 (current age: 18-24 years) | - | - | - |
| 1993-1998 (current age: 25-29 years) | 1.04 (1.01, 1.07) | 1.25 | 1.13 |
| 1983-1993 (current age: 30-39 years) | 0.99 (0.93, 1.06) | 1.11 | 1.00 |
| 1973-1983 (current age: 40-49 years) | 1.03 (0.94, 1.12) | 1.19 | 1.00 |
| 1963-1973 (current age: 50-59 years) | 1.04 (0.94, 1.14) | 1.23 | 1.00 |
| 1953-1963 (current age: 60-69 years) | 1.08 (0.95, 1.22) | 1.37 | 1.00 |
| 1943-1953 (current age: 70-79 years) | 1.20 (1.07, 1.33) | 1.68 | 1.35 |
| 1943 or earlier (current age: 80+ years) | 0.73 (0.23, 2.36) | 2.07 | 1.00 |
| **Gender** |  |  |  |
| Ref: Male | - | - | - |
| Female | 1.00 (0.97, 1.04) | 1.07 | 1.00 |
| Other | 0.06 (0.01, 0.54) | 34.12 | 3.12 |

Figure S1. Forest plot for association of very good/somewhat good relationship with mother when growing up (reference: very bad/somewhat bad) with dispositional forgivingness in adulthood


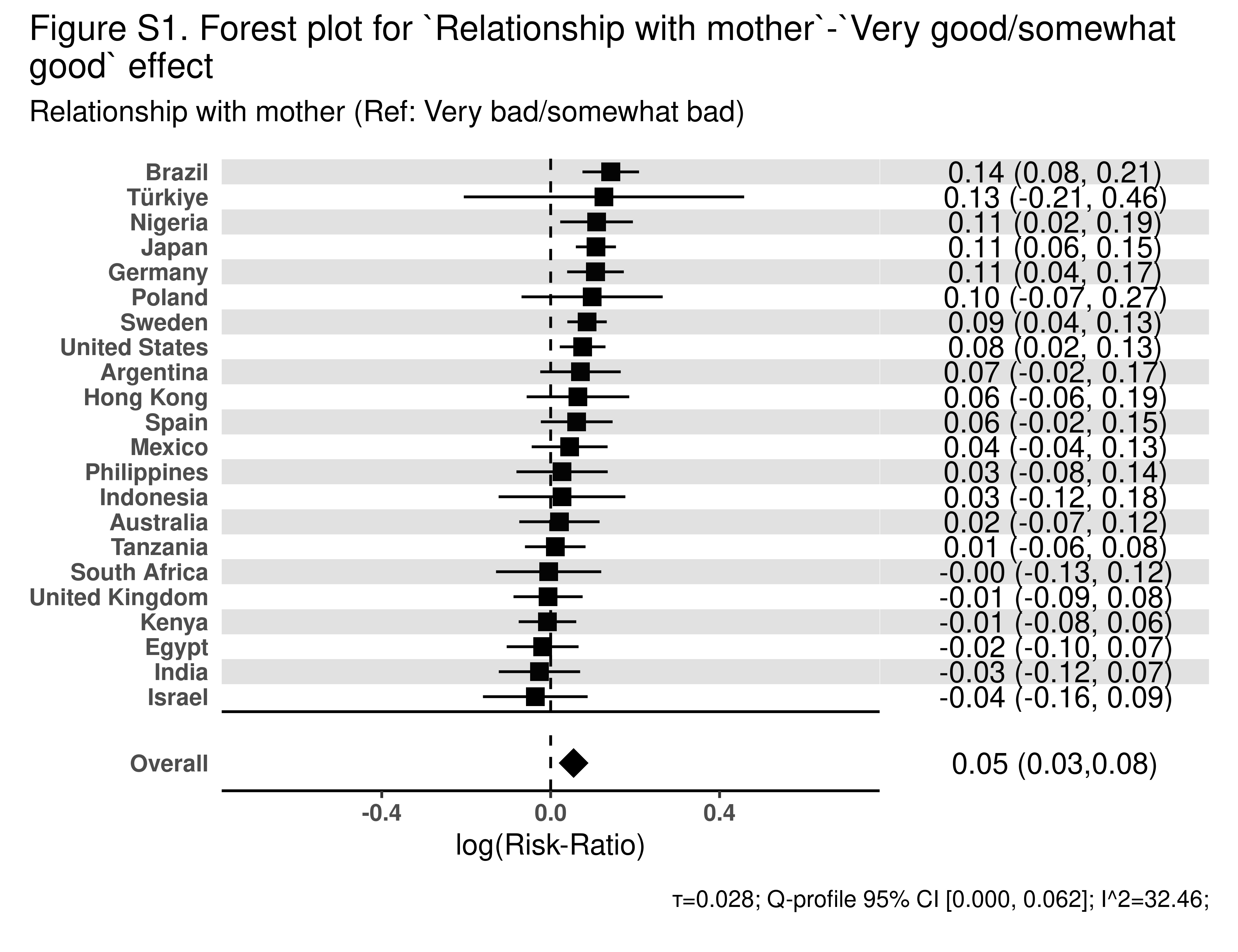


Figure S2. Forest plot for association of very good/somewhat good relationship with father when growing up (reference: very bad/somewhat bad) with dispositional forgivingness in adulthood


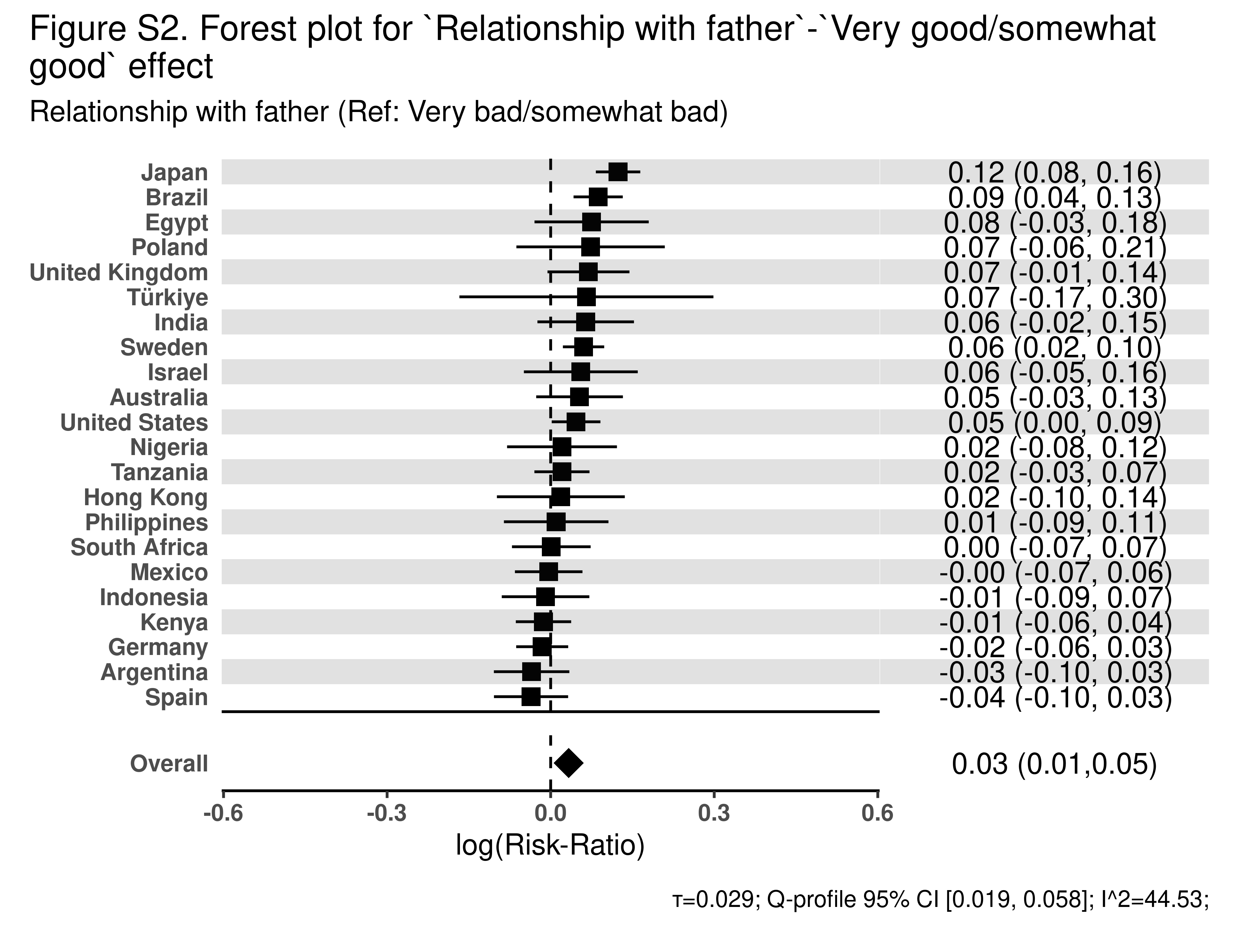


Figure S3. Forest plot for association of parents were divorced at around age 12 (reference: parents were married) with dispositional forgivingness in adulthood


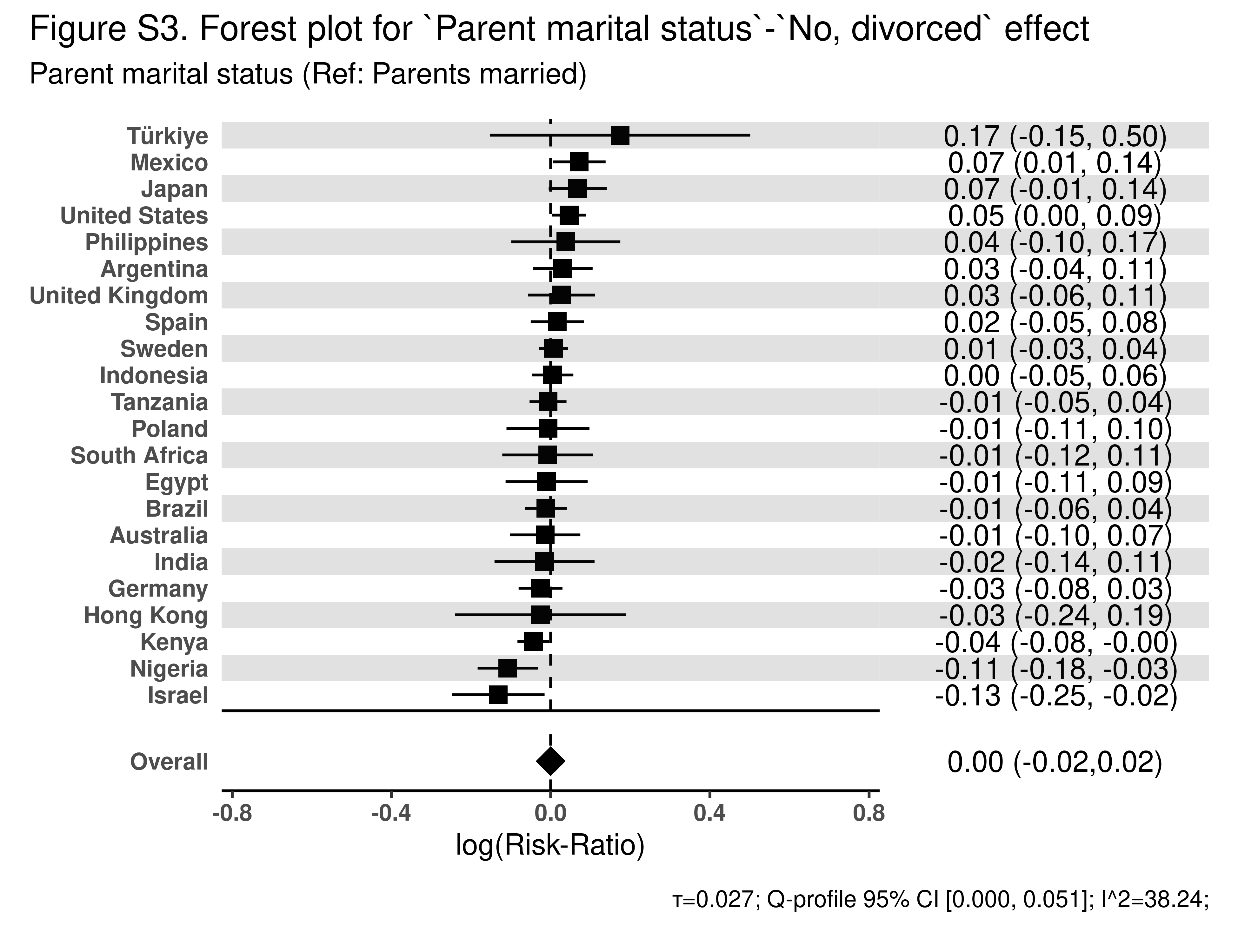


Figure S4. Forest plot for association of parents were never married at around age 12 (reference: parents were married) with dispositional forgivingness in adulthood


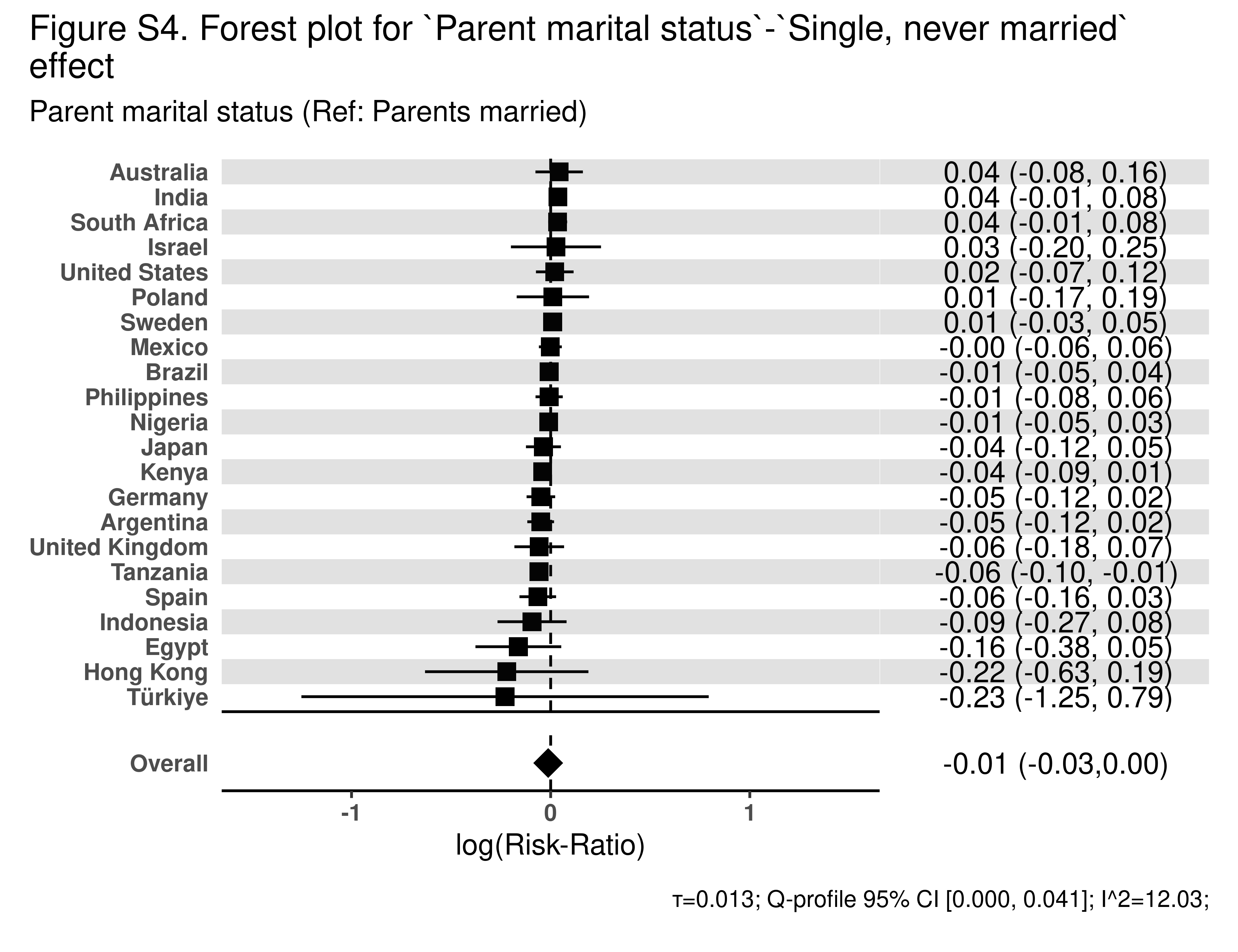


Figure S5. Forest plot for association of one or both parents had died at around age 12 (reference: parents were married) with dispositional forgivingness in adulthood


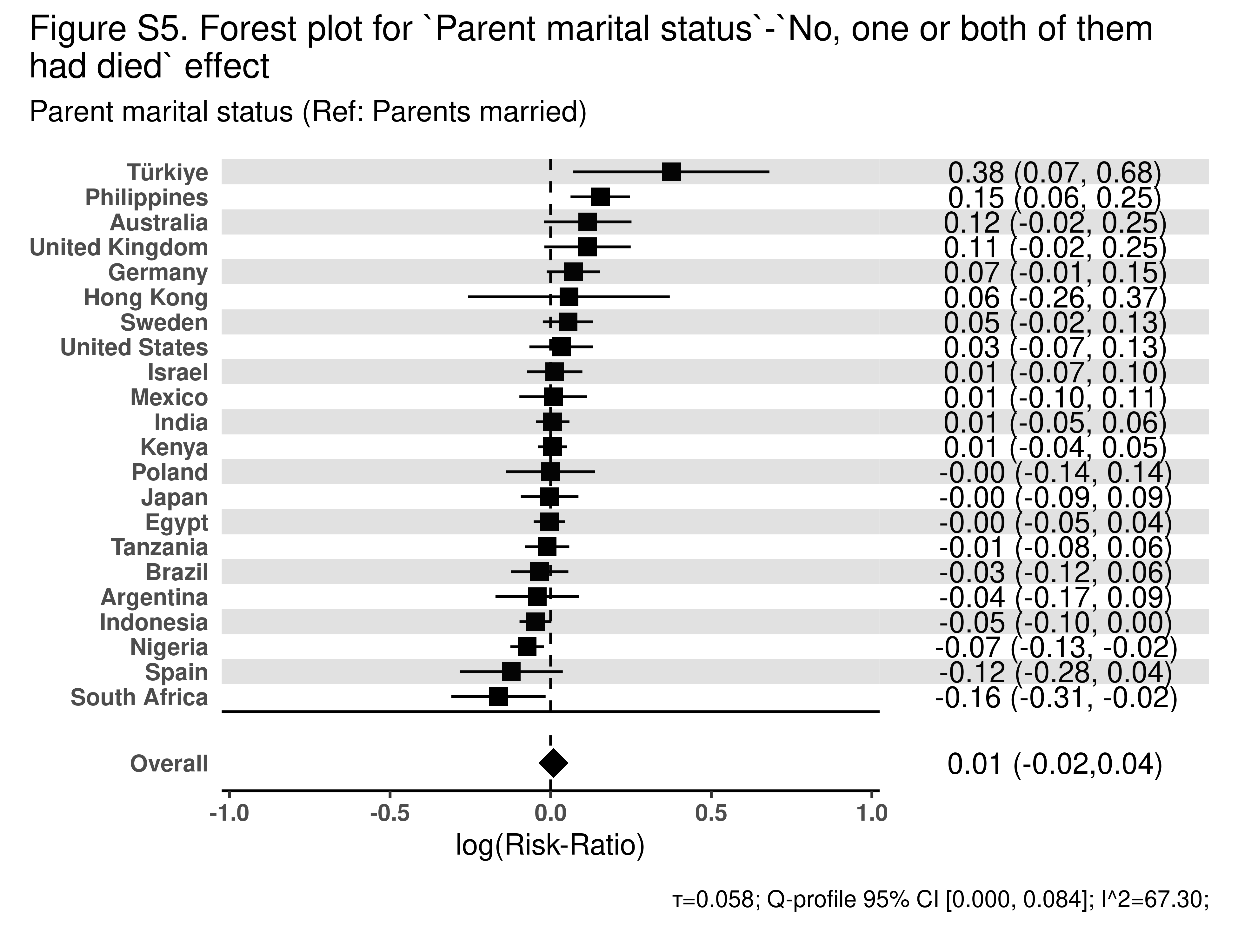


Figure S6. Forest plot for association of lived comfortably financially around age 12 (reference: got by) with dispositional forgivingness in adulthood


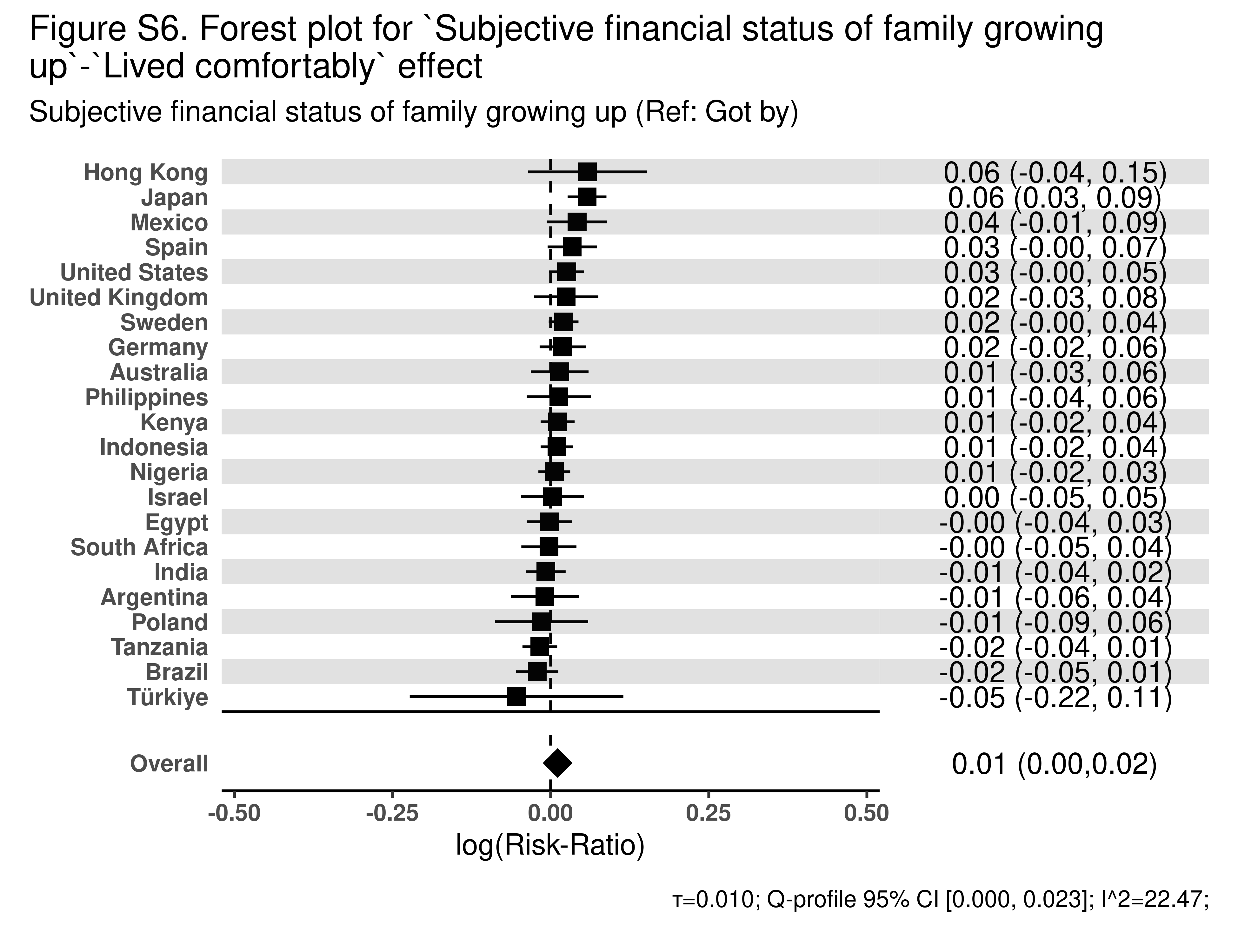


Figure S7. Forest plot for association of found it difficult financially around age 12 (reference: got by) with dispositional forgivingness in adulthood


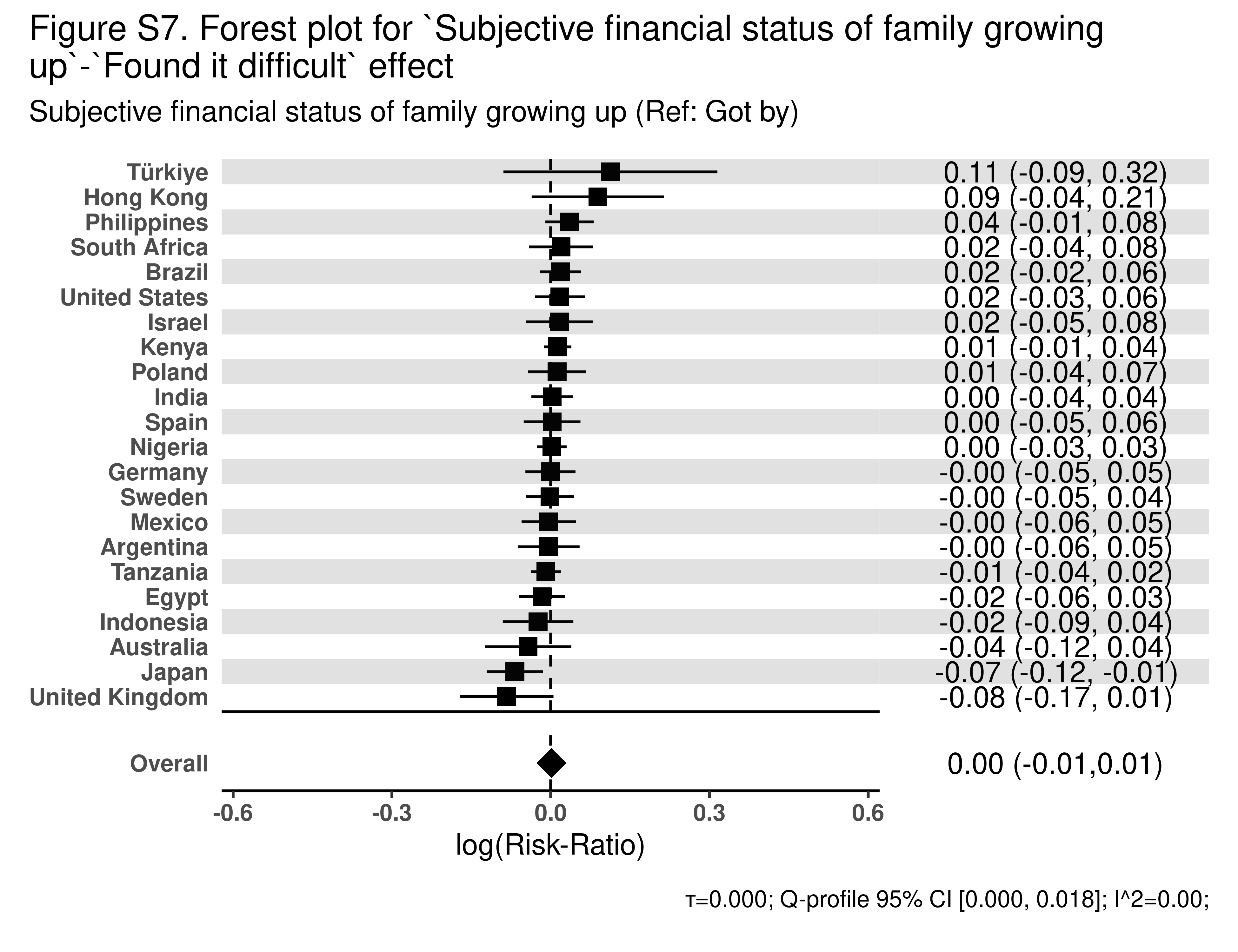


Figure S8. Forest plot for association of found it very difficult financially around age 12 (reference: got by) with dispositional forgivingness in adulthood


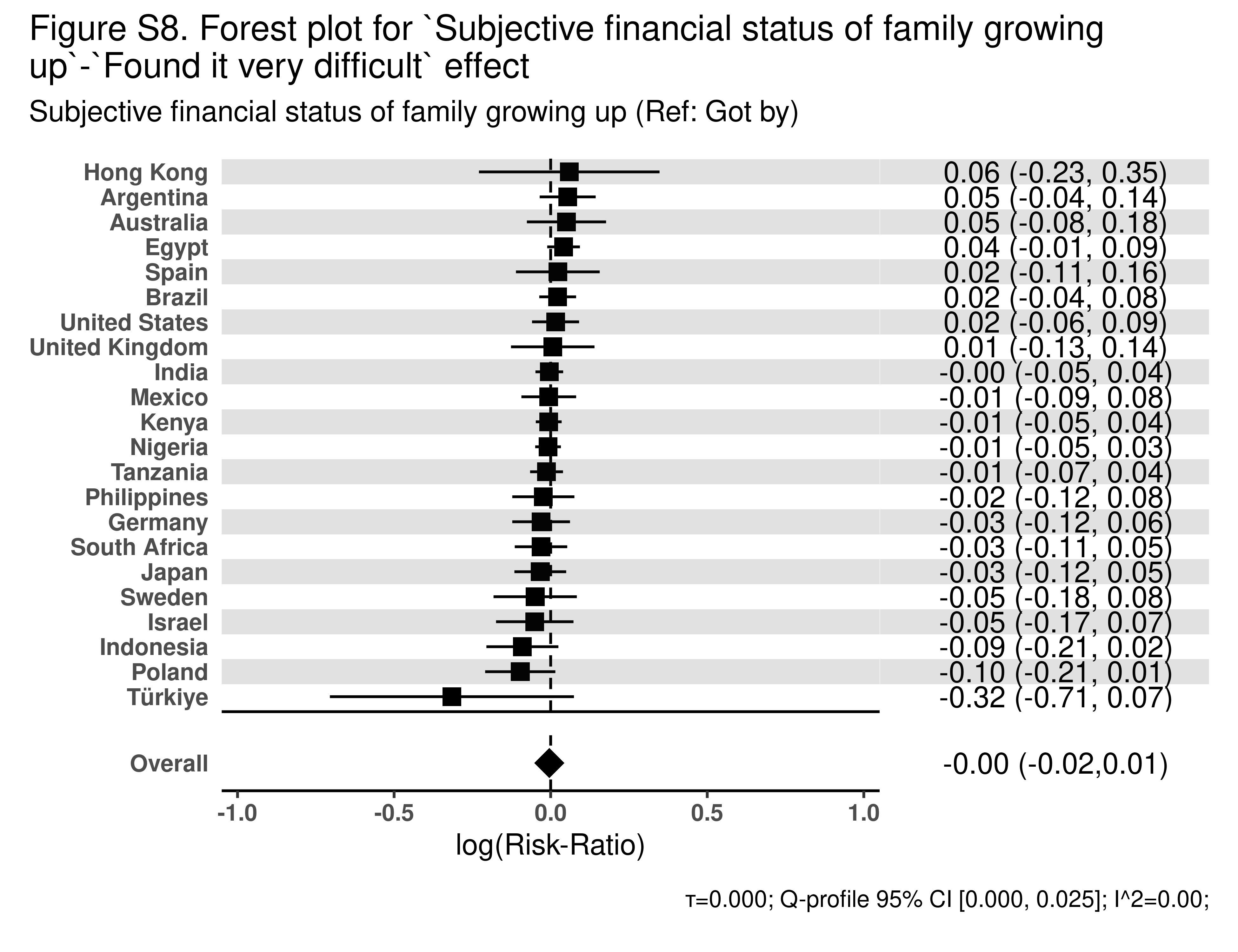


Figure S9. Forest plot for association of experienced abuse when growing up (reference: no) with dispositional forgivingness in adulthood


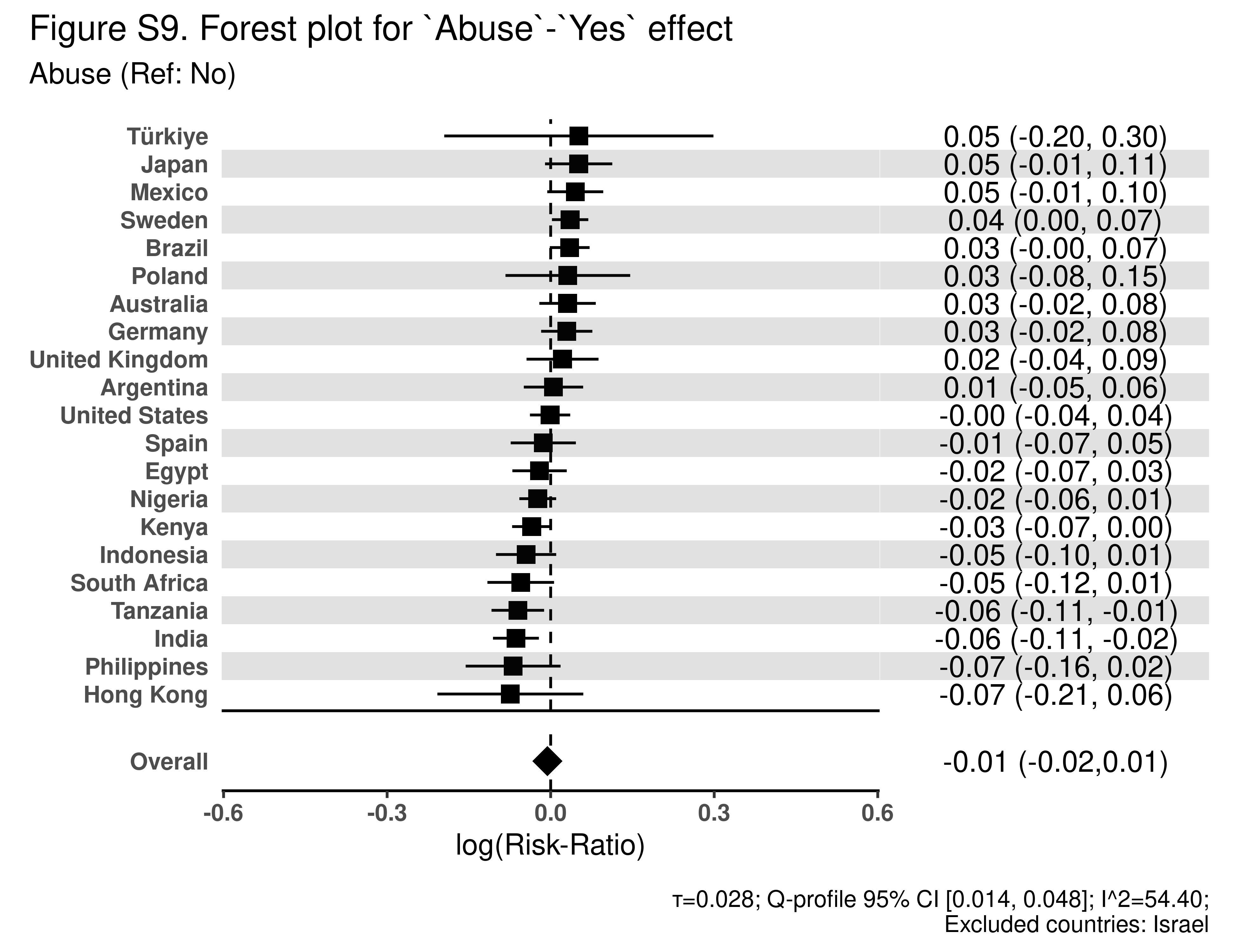


Figure S10. Forest plot for association of felt like an outsider in the family when growing up (reference: no) with dispositional forgivingness in adulthood


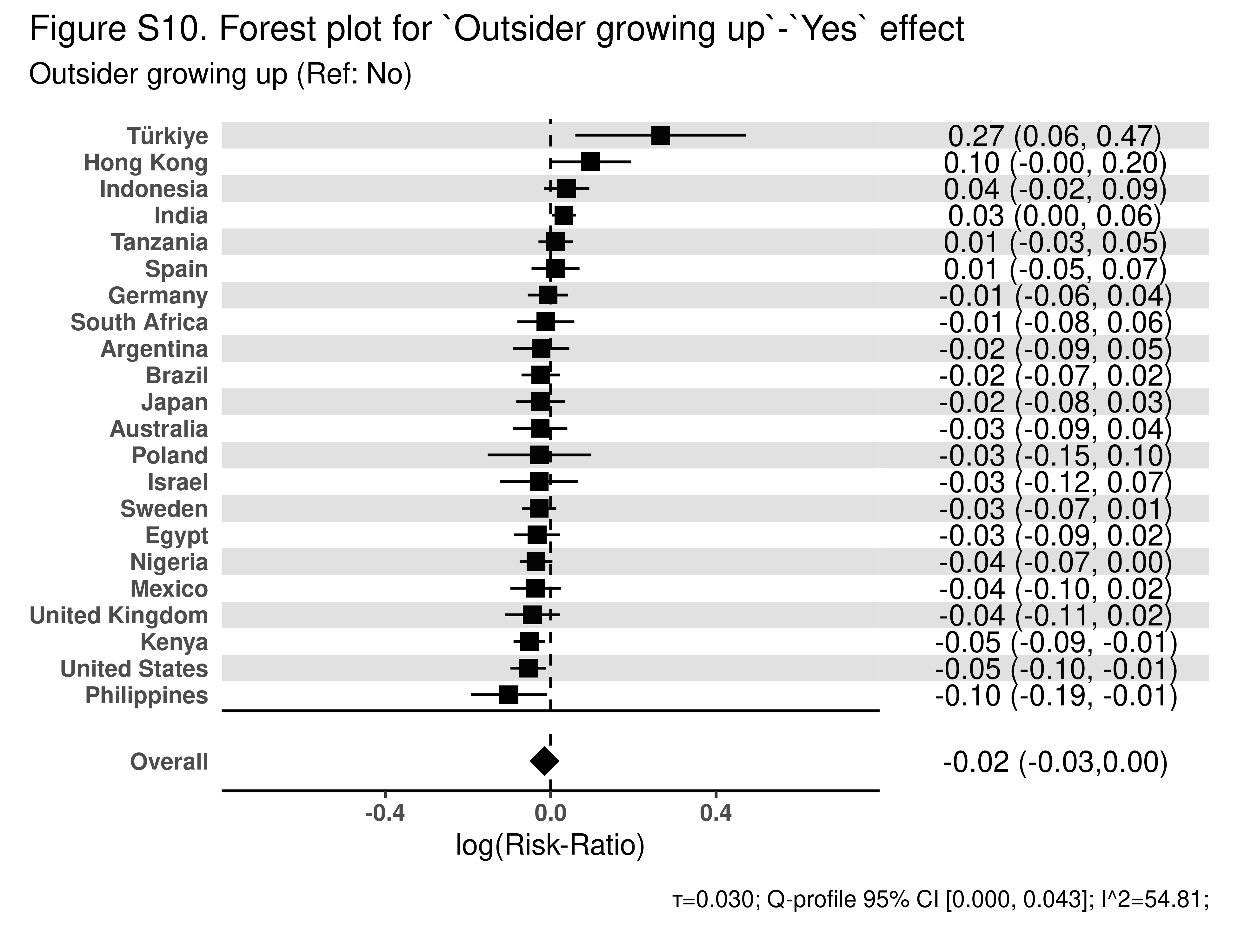


Figure S11. Forest plot for association of excellent health when growing up (reference: good) with dispositional forgivingness in adulthood


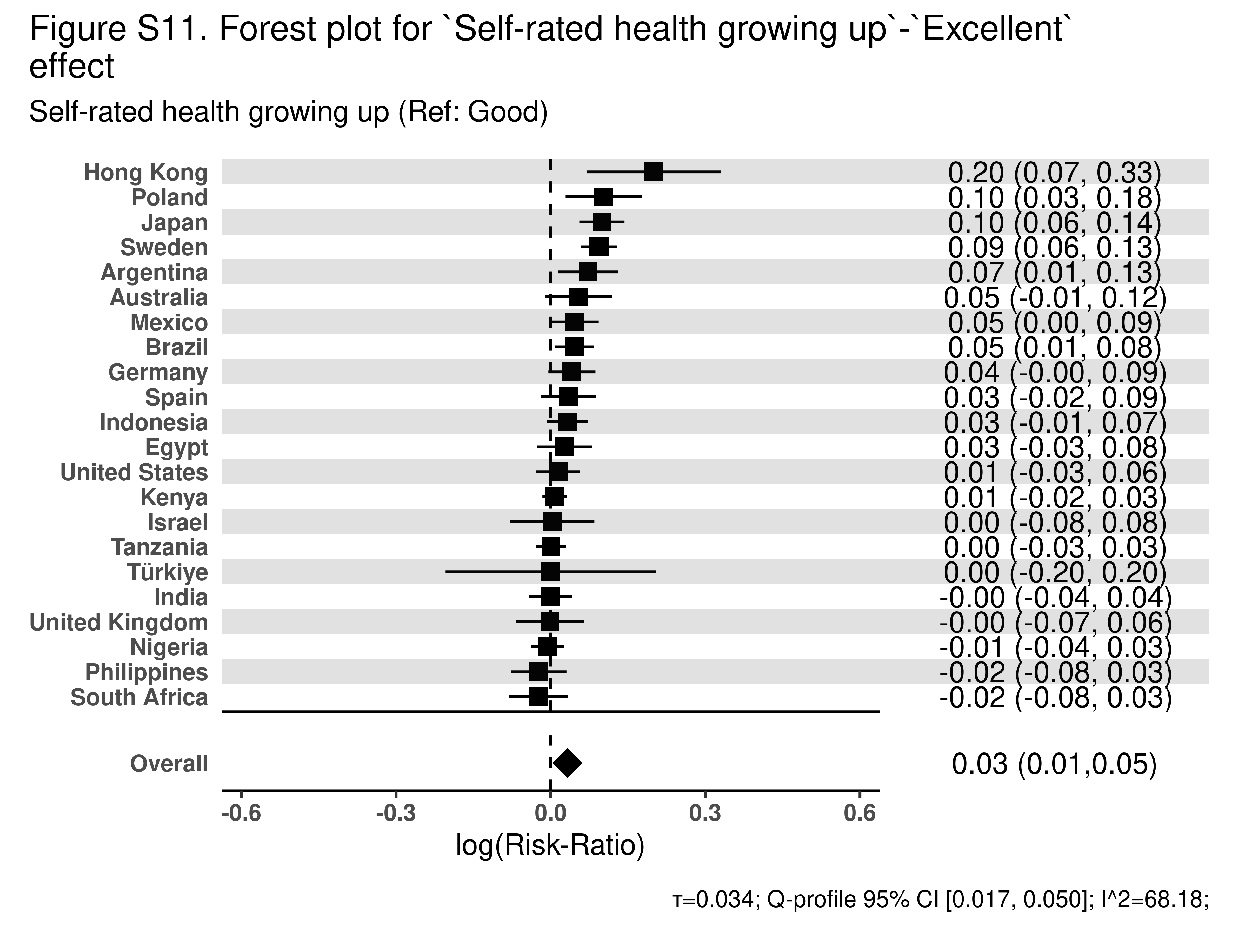


Figure S12. Forest plot for association of very good health when growing up (reference: good) with dispositional forgivingness in adulthood


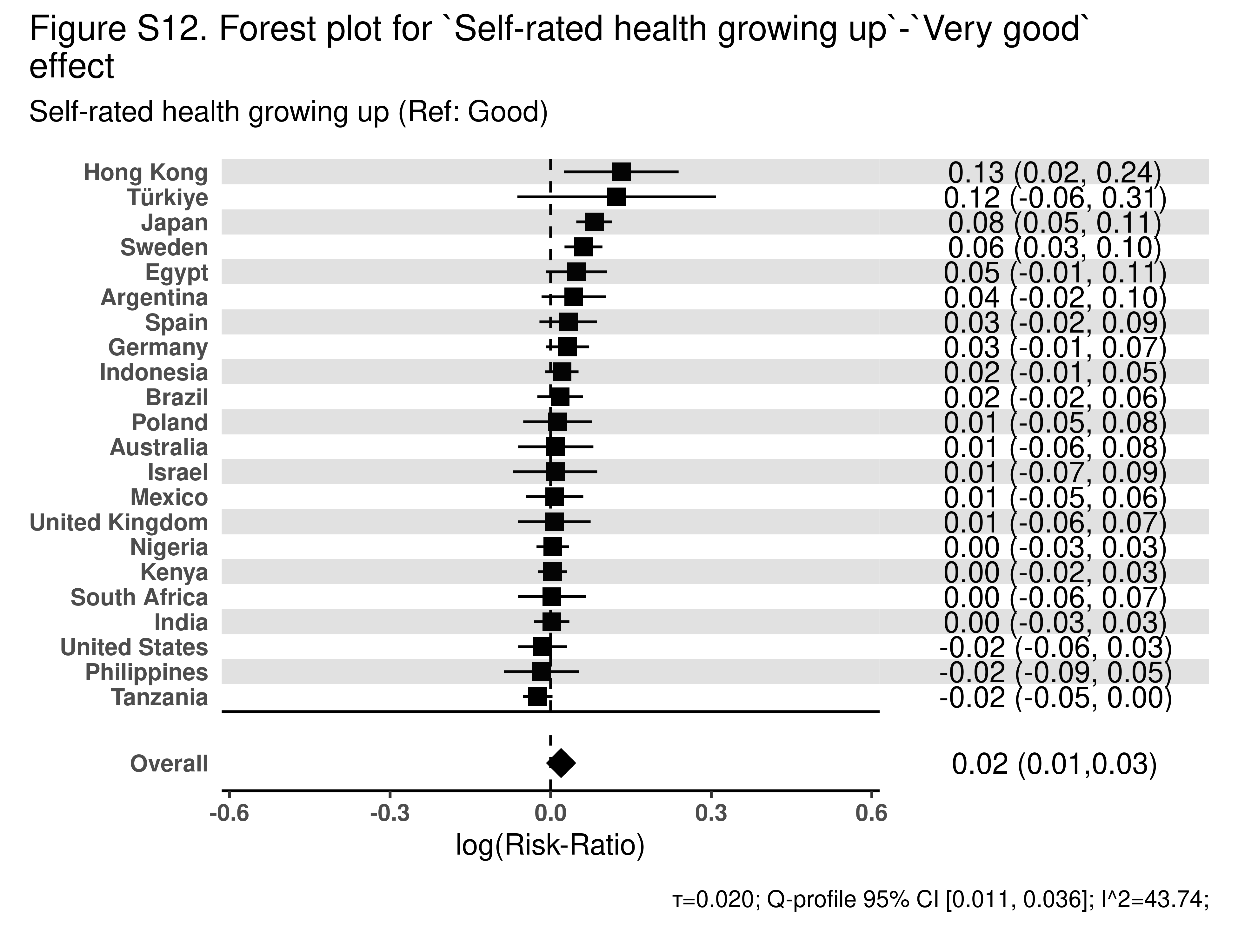


Figure S13. Forest plot for association of fair health when growing up (reference: good) with dispositional forgivingness in adulthood


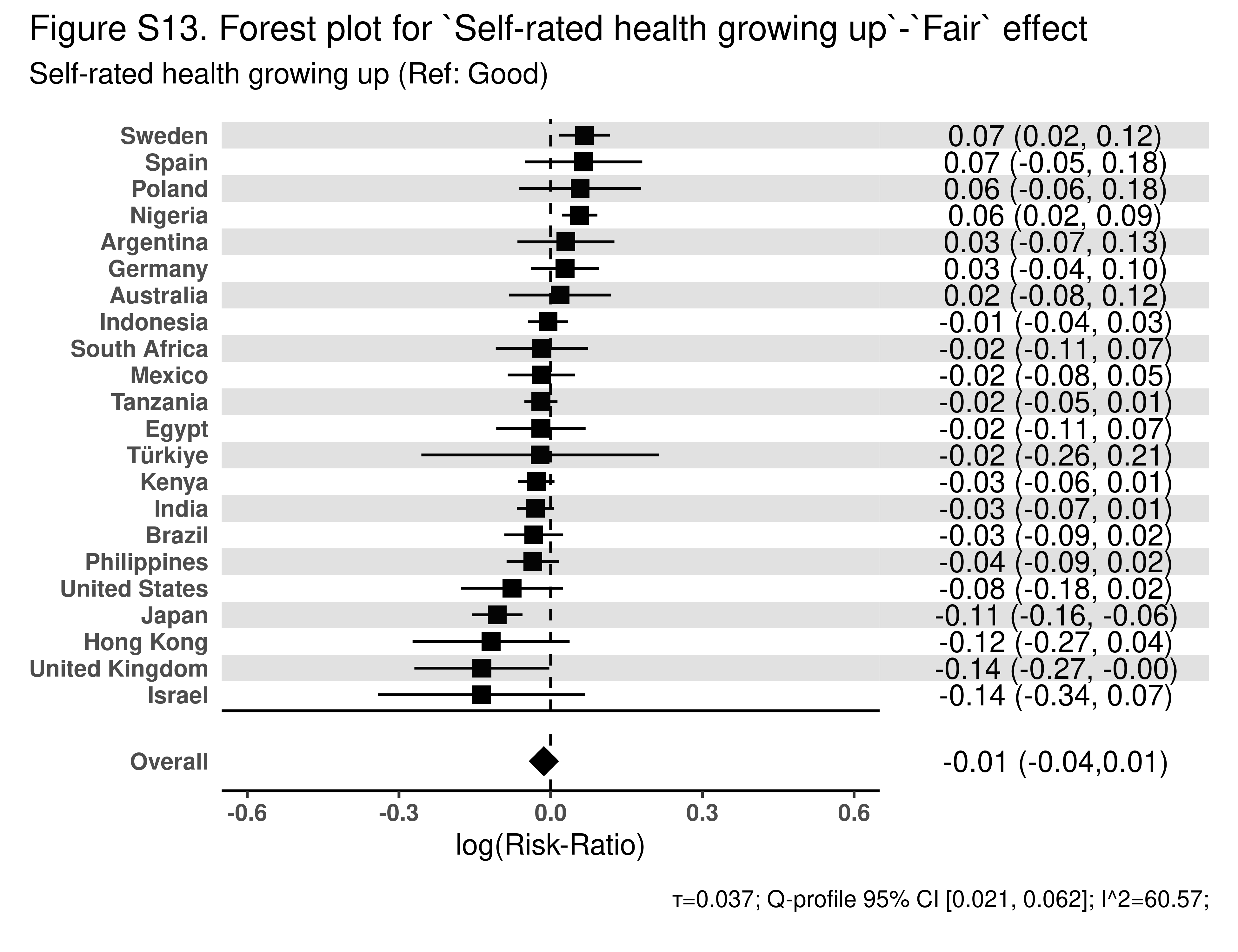


Figure S14. Forest plot for association of poor health when growing up (reference: good) with dispositional forgivingness in adulthood


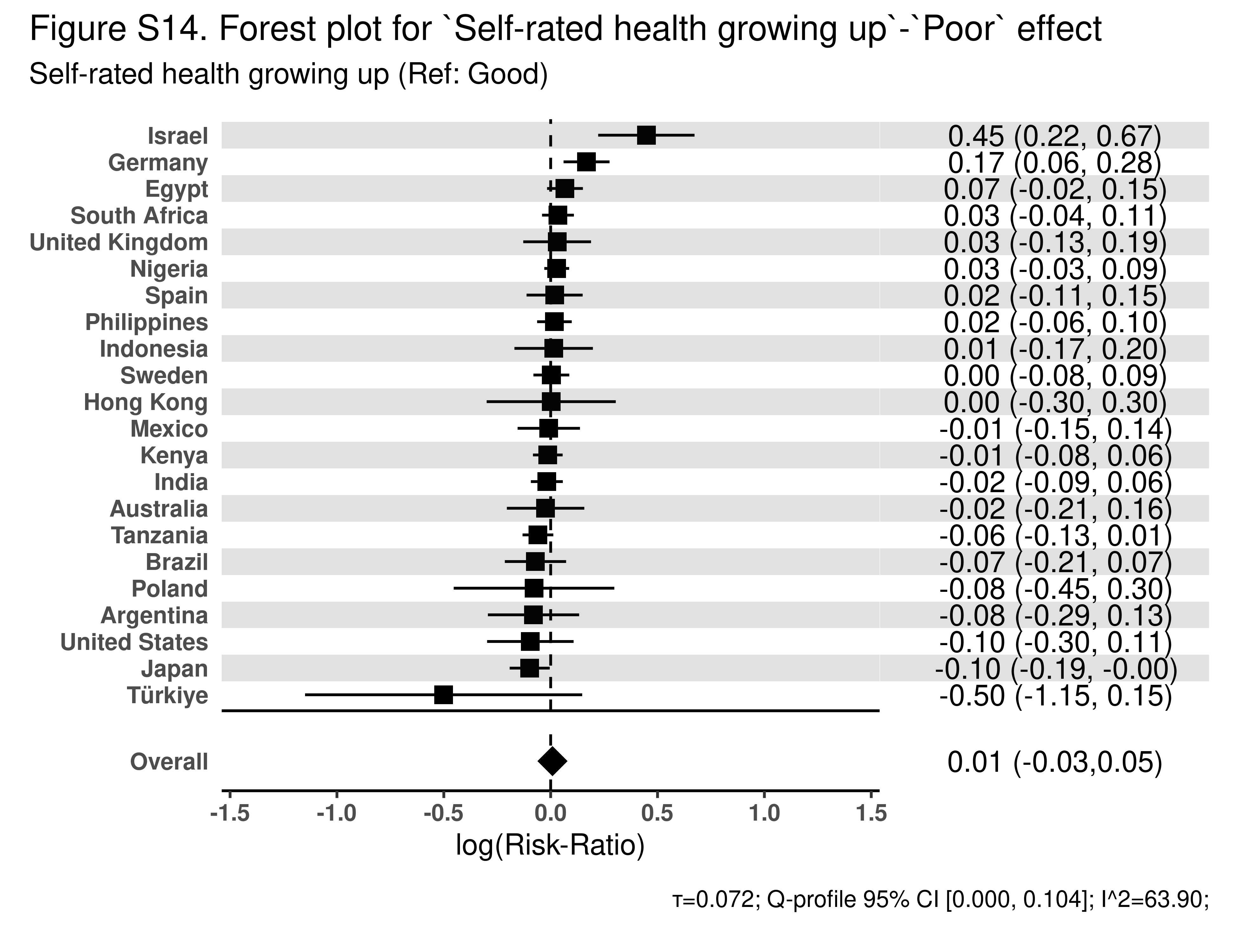


Figure S15. Forest plot for association of born in another country (reference: born in this country) with dispositional forgivingness in adulthood


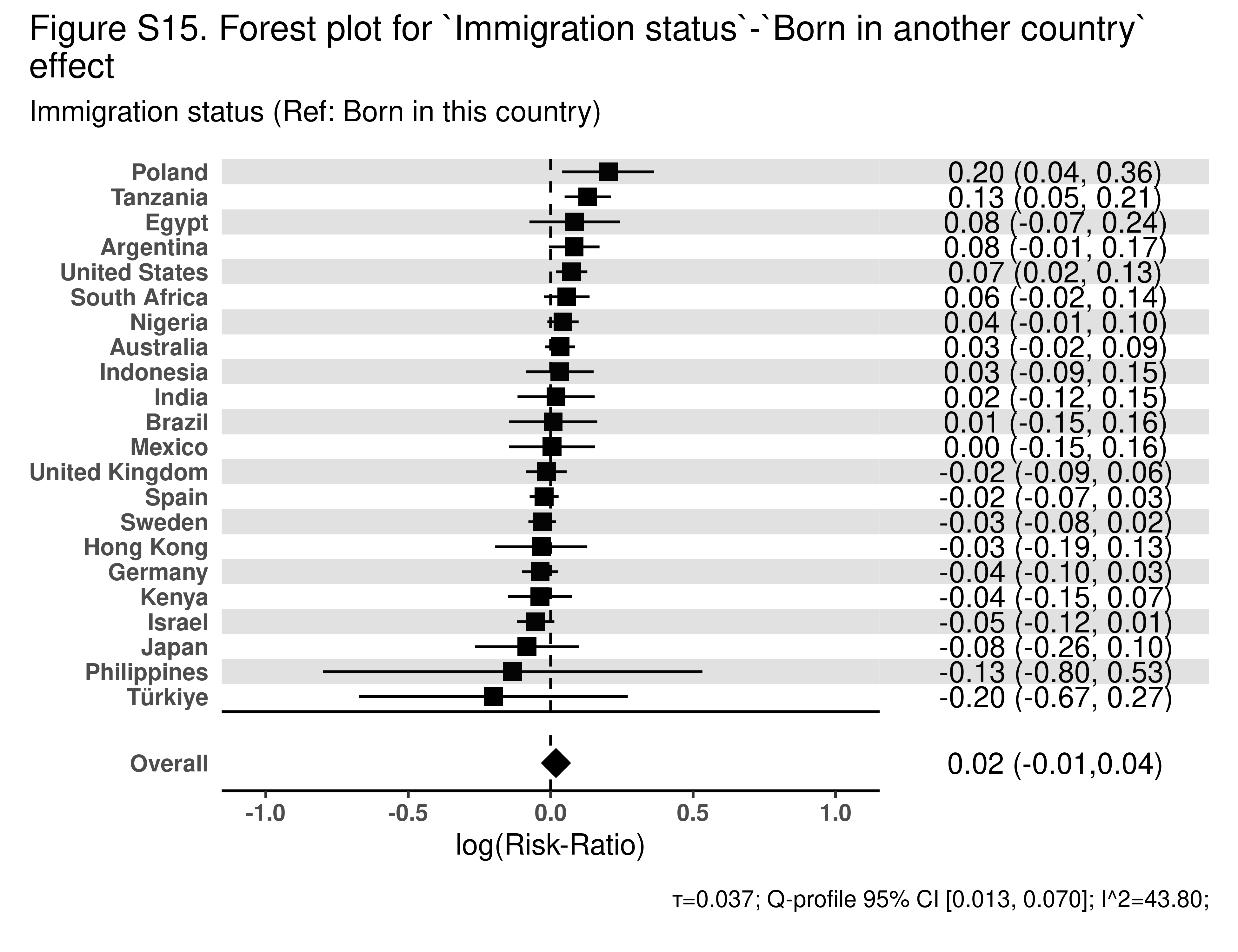


Figure S16. Forest plot for association of attending religious services ≥ 1/week around age 12 (reference: never) with dispositional forgivingness in adulthood


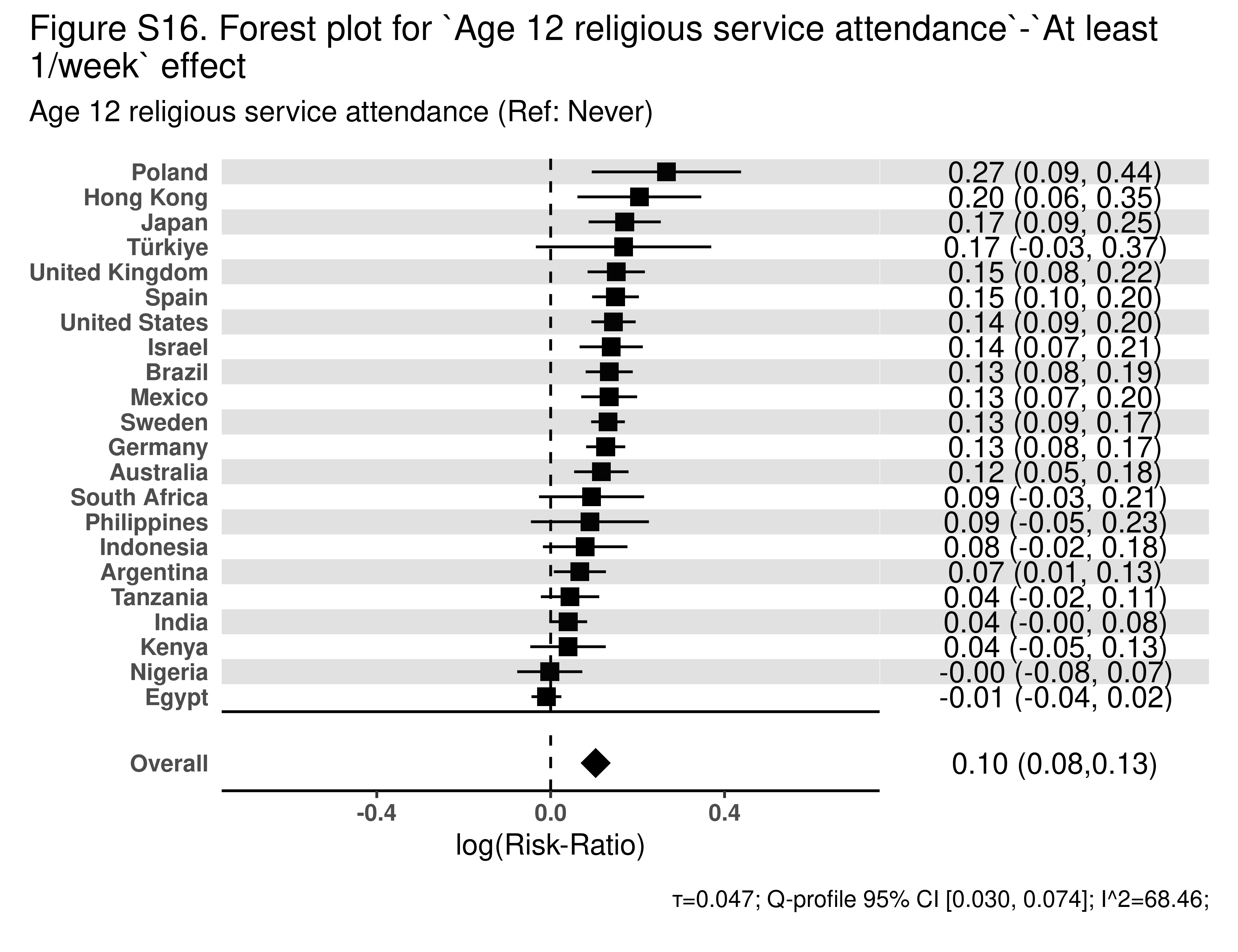


Figure S17. Forest plot for association of attending religious services 1-3/month around age 12 (reference: never) with dispositional forgivingness in adulthood


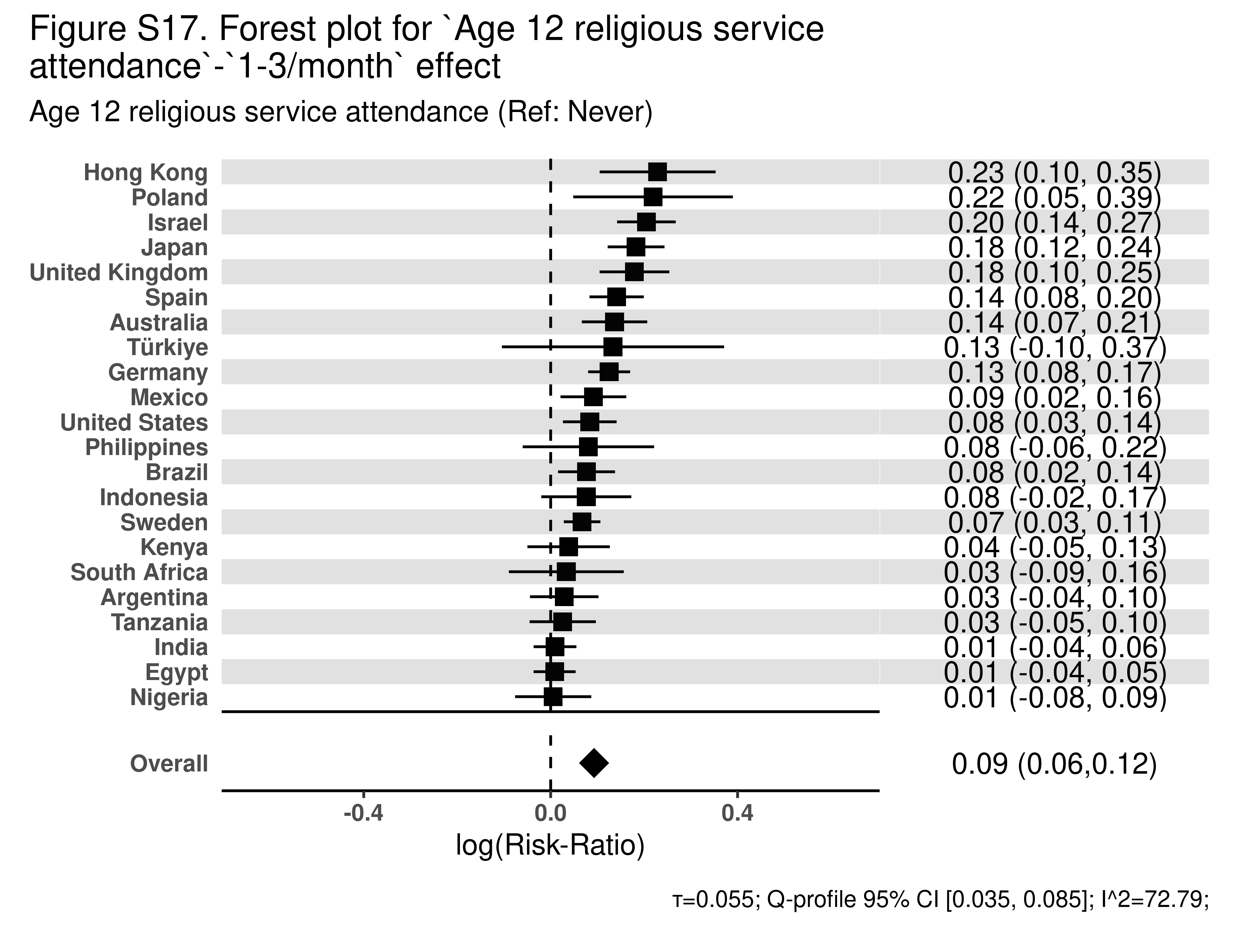


Figure S18. Forest plot for association of attending religious services < 1/month around age 12 (reference: never) with dispositional forgivingness in adulthood


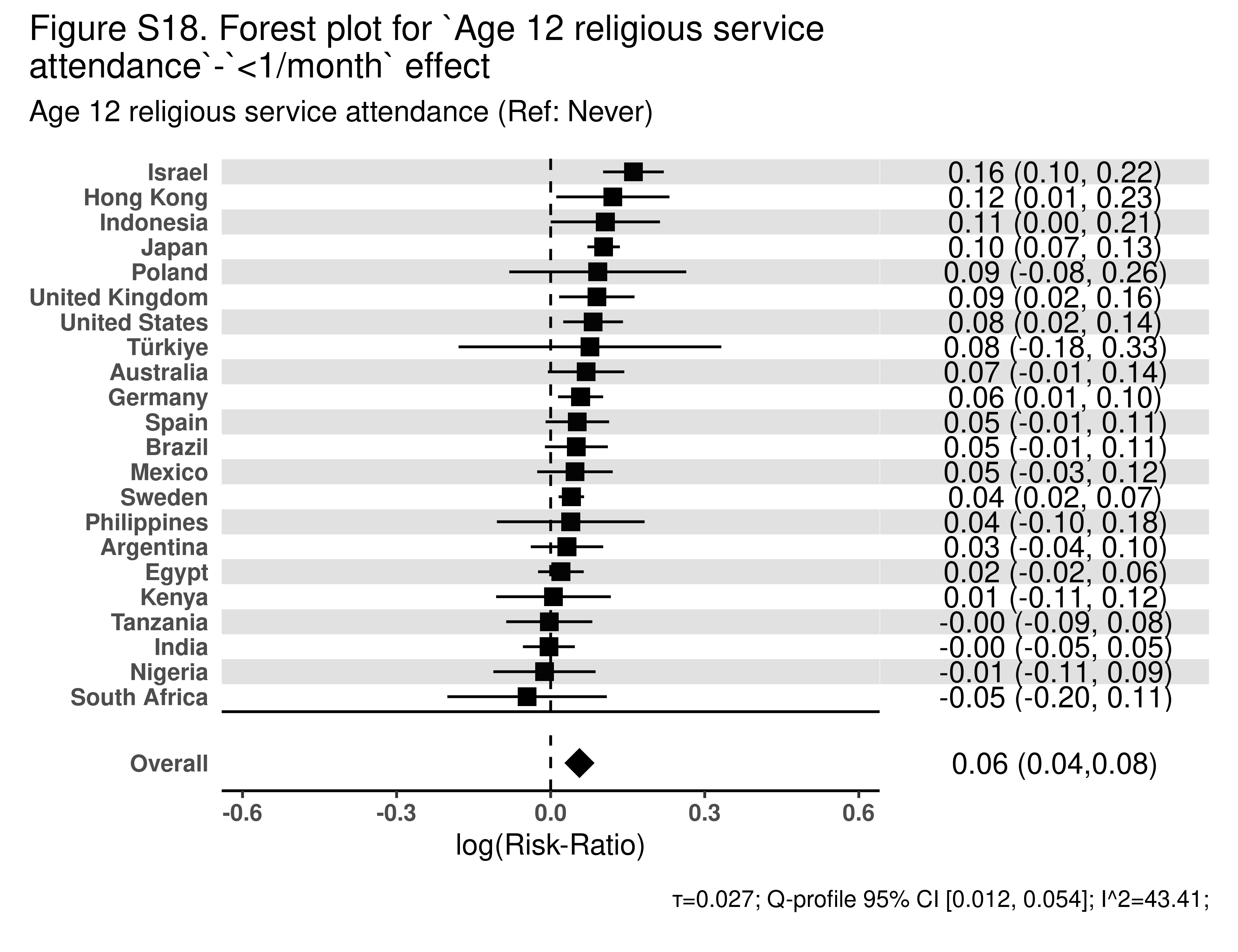


Figure S19. Forest plot for association of 1993-1998 year of birth (reference: 1998-2005 year of birth) with dispositional forgivingness in adulthood


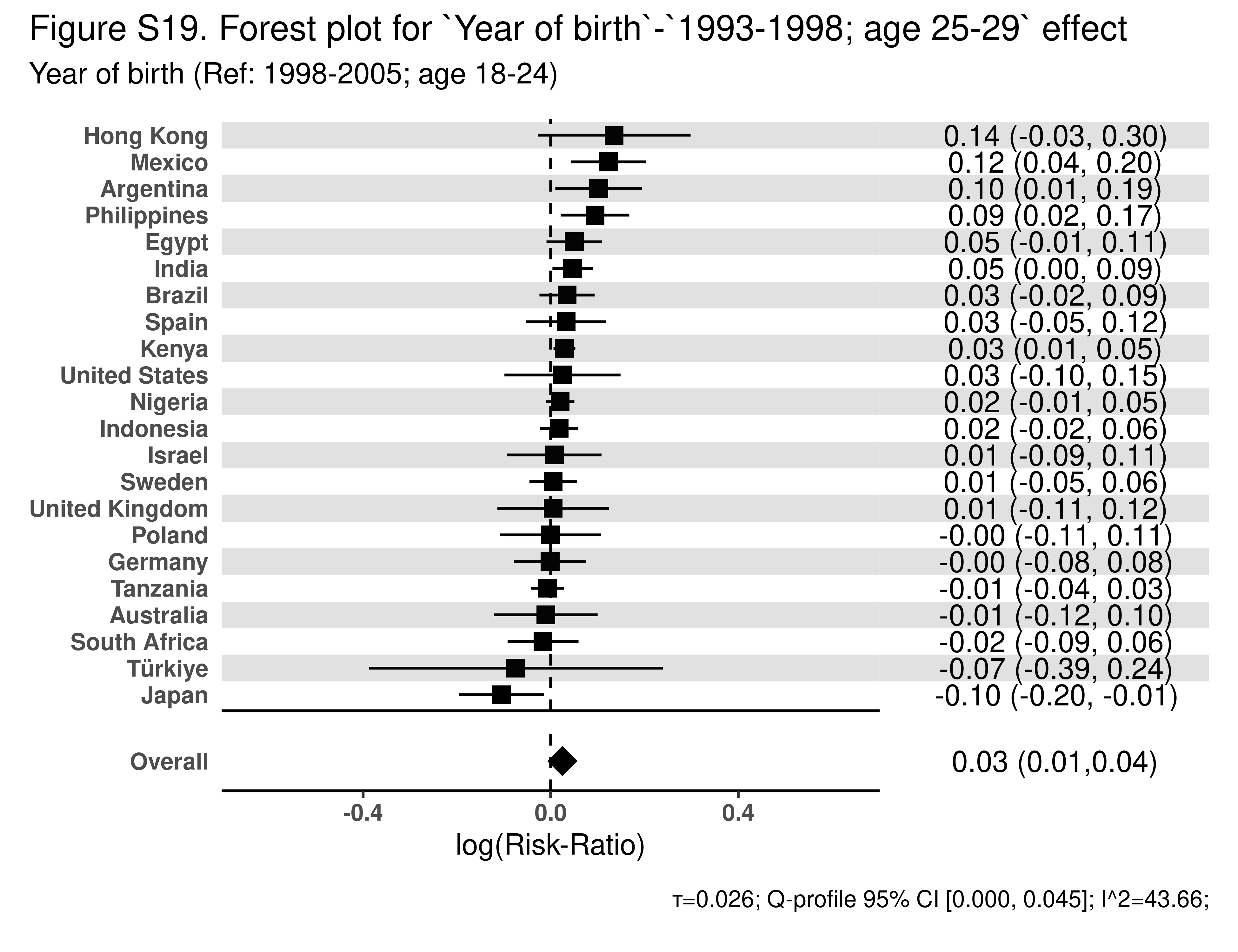


Figure S20. Forest plot for association of 1983-1993 year of birth (reference: 1998-2005 year of birth) with dispositional forgivingness in adulthood


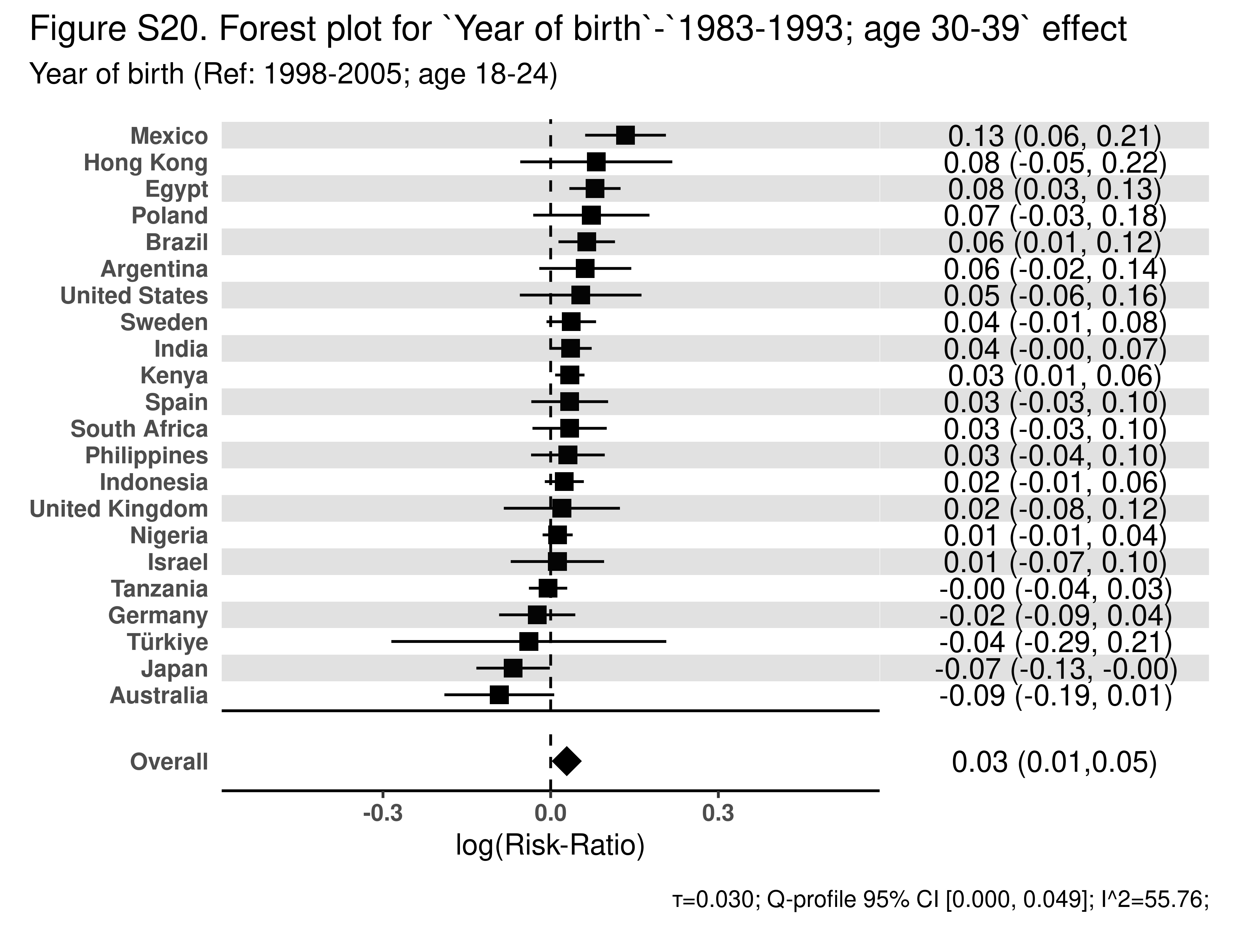


Figure S21. Forest plot for association of 1973-1983 year of birth (reference: 1998-2005 year of birth) with dispositional forgivingness in adulthood


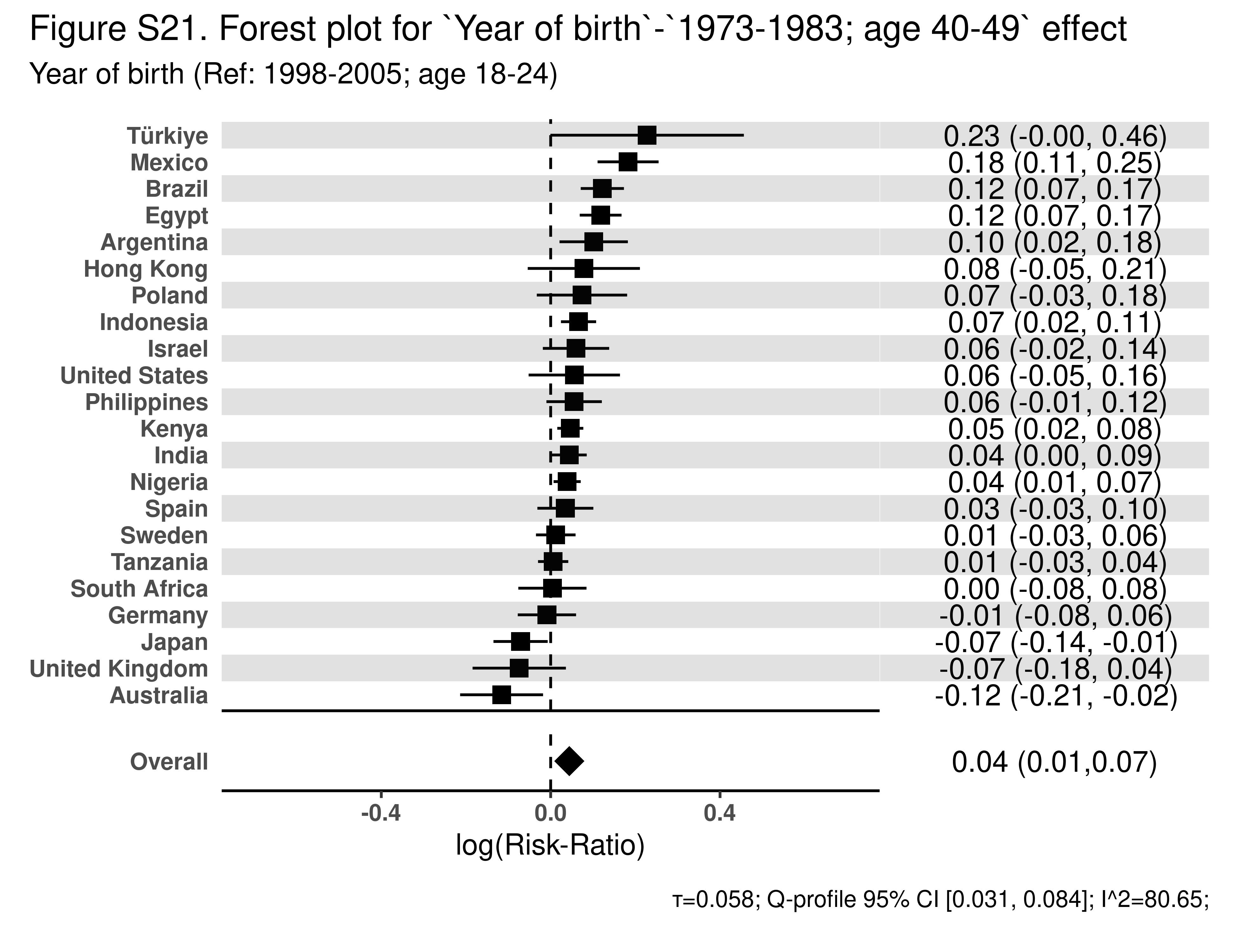


Figure S22. Forest plot for association of 1963-1973 year of birth (reference: 1998-2005 year of birth) with dispositional forgivingness in adulthood


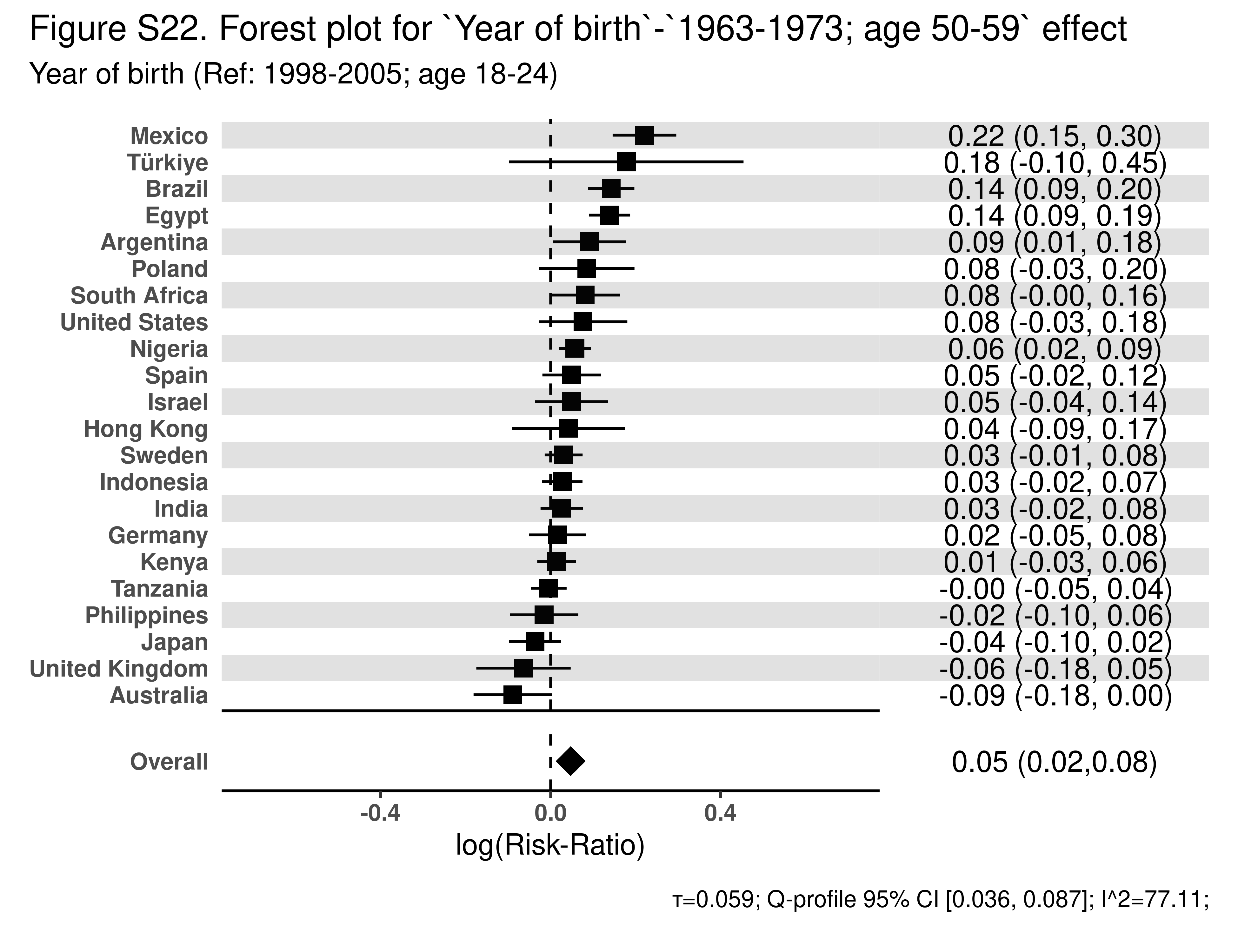


Figure S23. Forest plot for association of 1953-1963 year of birth (reference: 1998-2005 year of birth) with dispositional forgivingness in adulthood


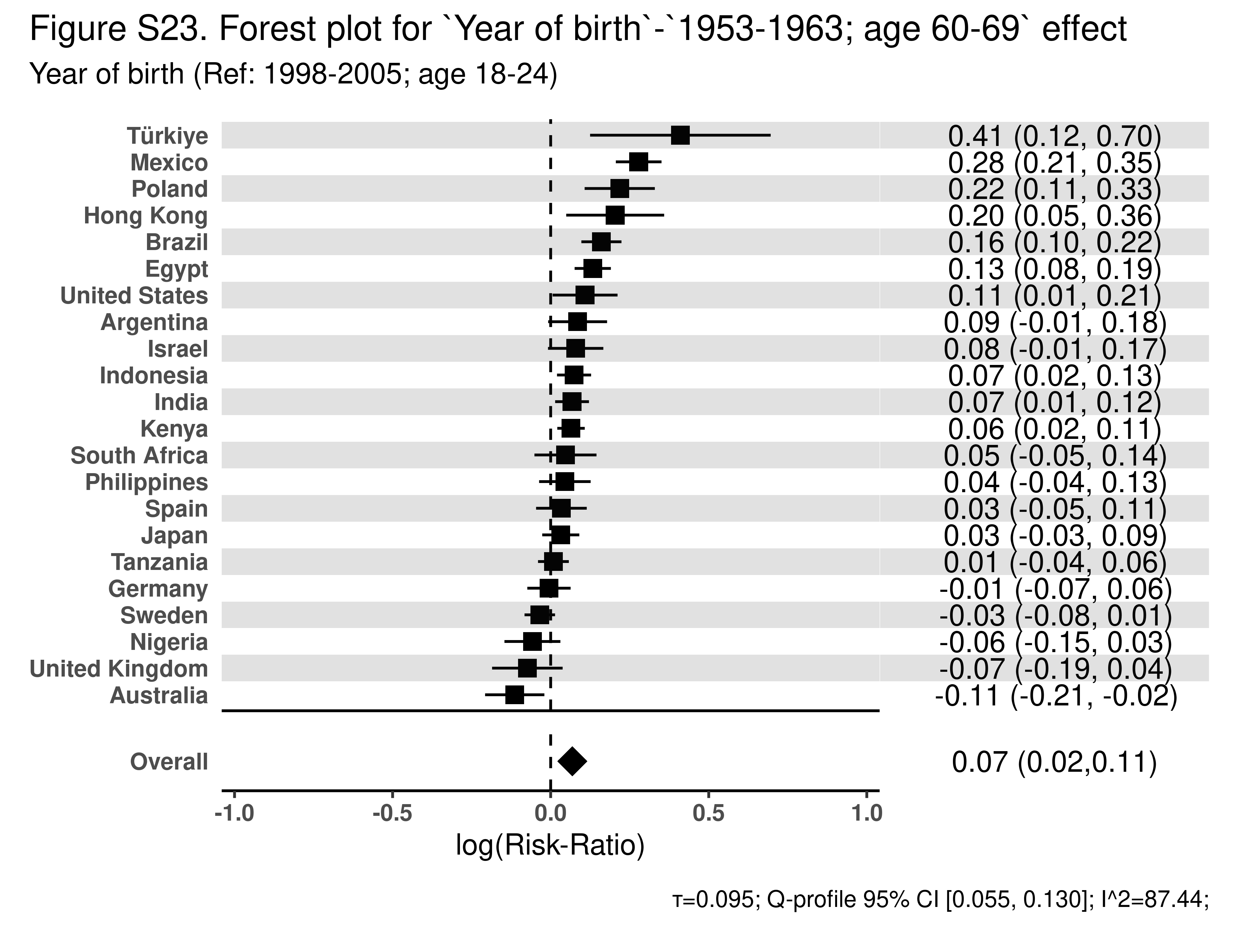


Figure S24. Forest plot for association of 1943-1953 year of birth (reference: 1998-2005 year of birth) with dispositional forgivingness in adulthood


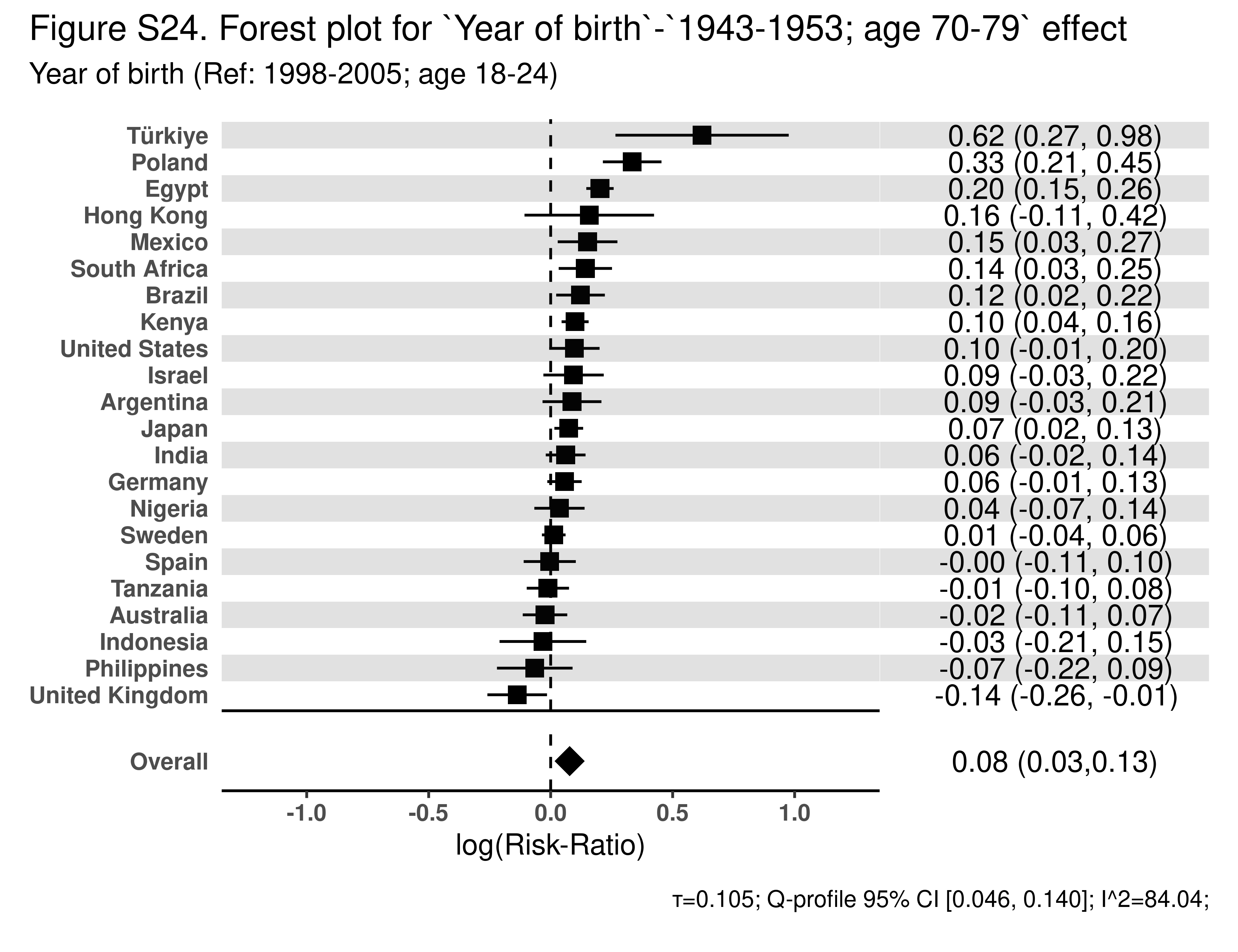


Figure S25. Forest plot for association of 1943 or earlier year of birth (reference: 1998-2005 year of birth) with dispositional forgivingness in adulthood


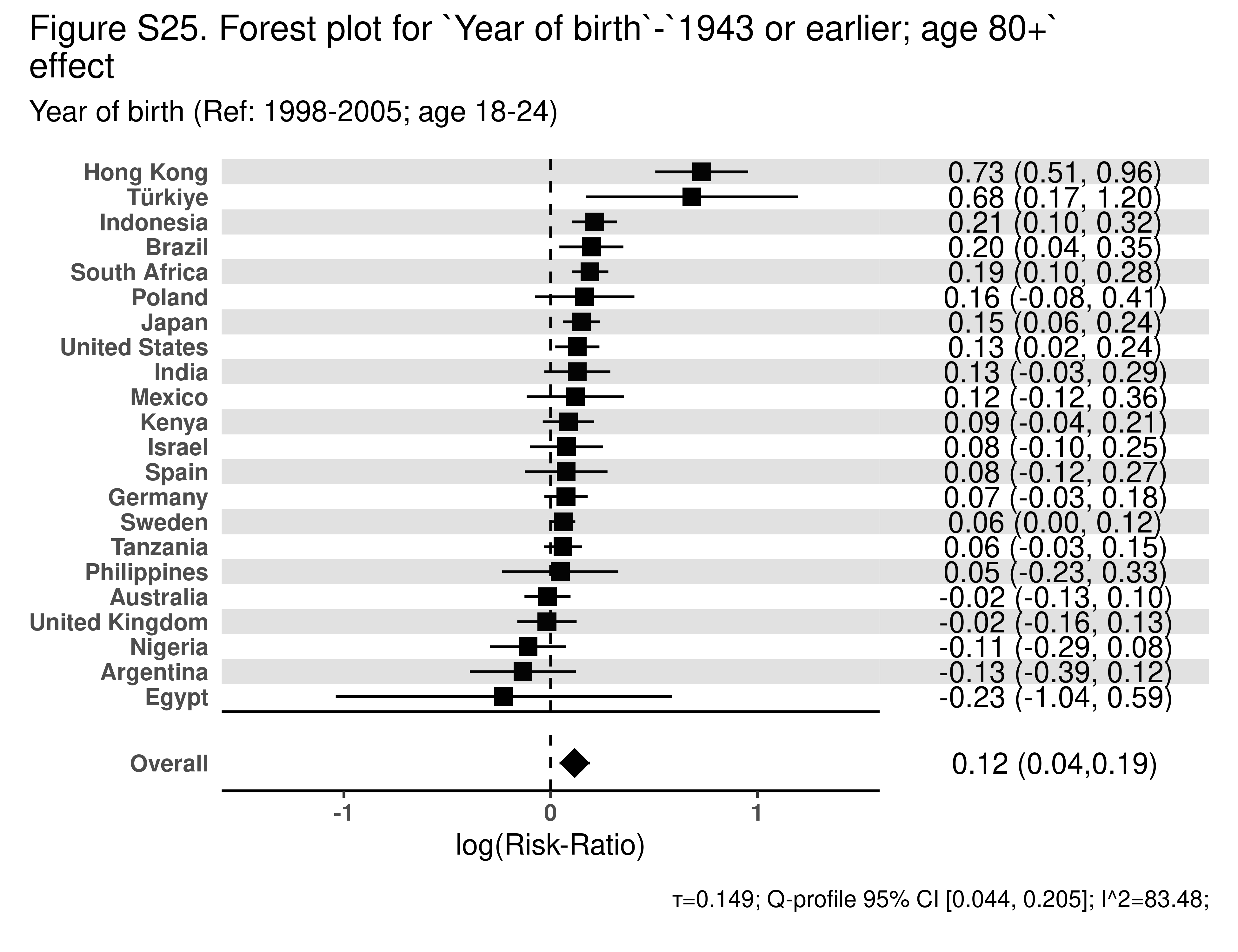


Figure S26. Forest plot for association of female (reference: male) with dispositional forgivingness in adulthood


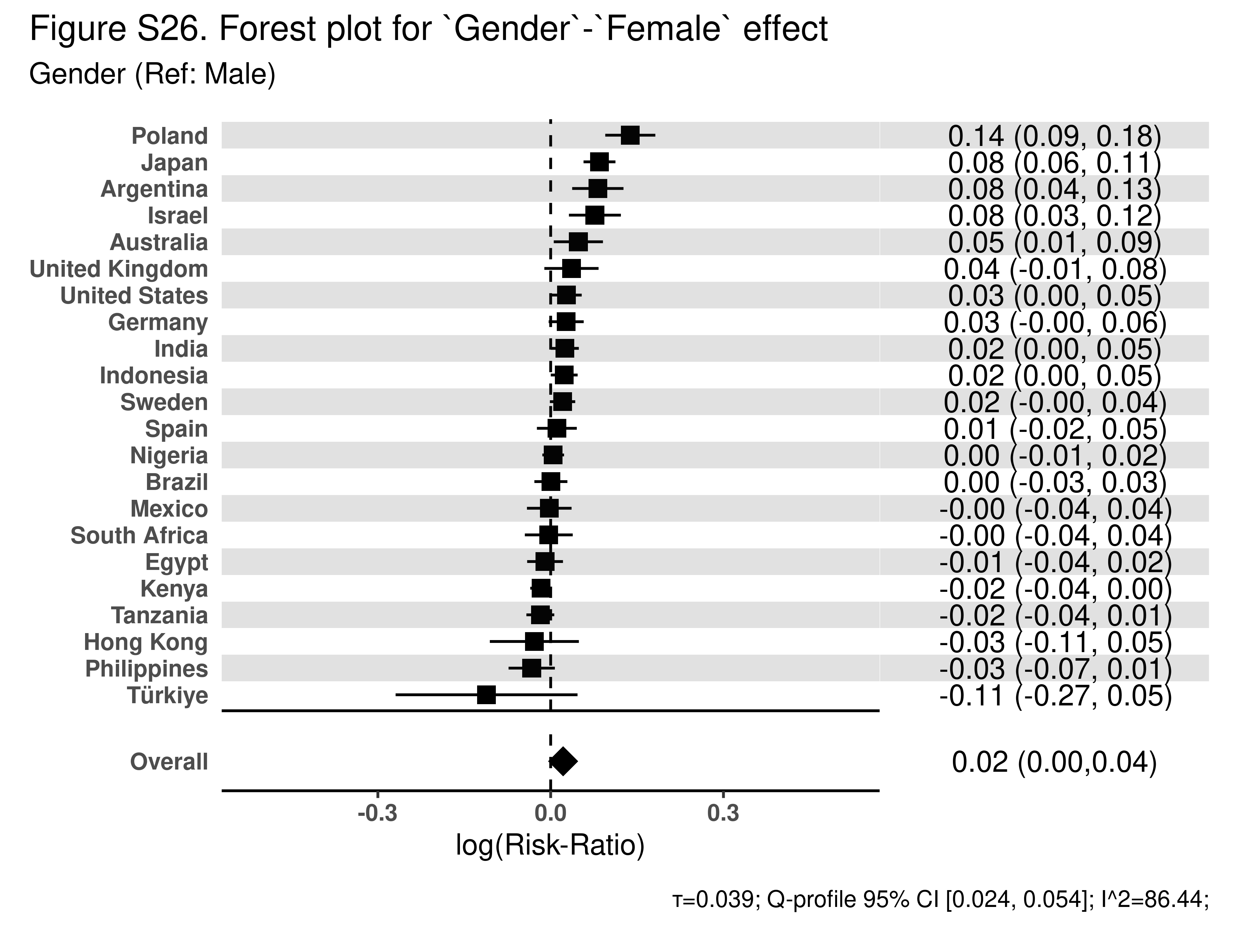


Figure S27. Forest plot for association of ‘other’ gender (reference: male) with dispositional forgivingness in adulthood


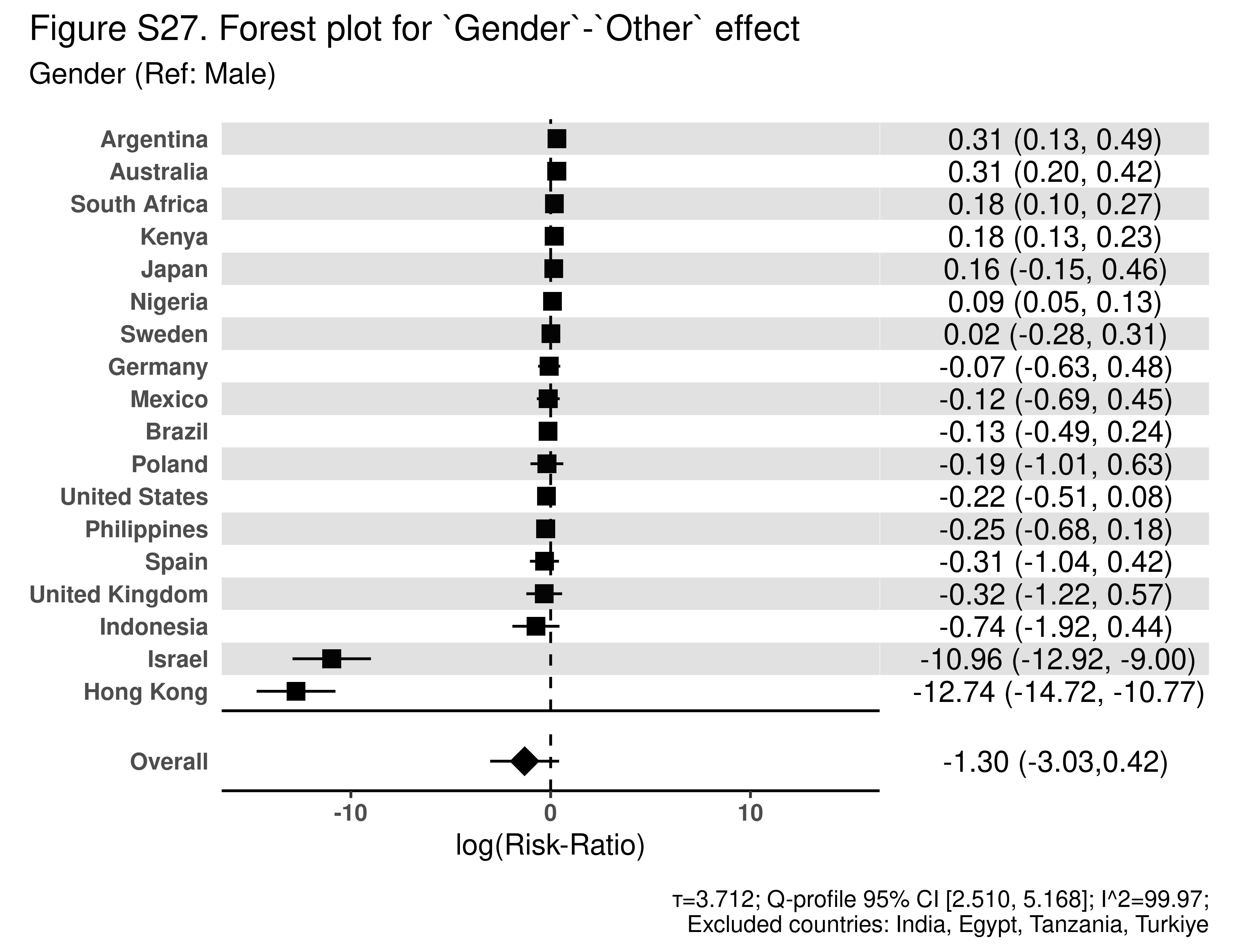

Supplement: Supplementary file 1 — Supplementary Material 1 [file 11482_2025_10451_MOESM1_ESM.docx]
